# Performance of an Xpert-based diagnostic algorithm for the rapid detection of drug-resistant tuberculosis among high-risk populations in a low-incidence setting

**DOI:** 10.1371/journal.pone.0200755

**Published:** 2018-07-16

**Authors:** Ting-Yi Chiang, Shin-Yuan Fan, Ruwen Jou

**Affiliations:** 1 Tuberculosis Research Center, Centers for Disease Control, Taipei, Taiwan, R.O.C.; 2 Research and Diagnostic Center, Centers for Disease Control, Taipei, Taiwan, R.O.C.; 3 Institute of Microbiology and Immunology, National Yang-Ming University, Taipei, Taiwan, R.O.C.; Indian Institute of Science, INDIA

## Abstract

Timely diagnosis of drug-resistant tuberculosis (DR-TB) is beneficial for case treatment and management. We implemented an algorithm to improve molecular diagnostic utilization to intensify DR-TB case findings. The GeneXpert MTB/RIF (Xpert) test was used for initial diagnosis. Samples with *Mycobacterium tuberculosis* complex (MTBC)-positive and rifampicin resistance (RR) results were subsequently and simultaneously tested using the GenoType MTBDR*plus* (DR*plus*) and MTBDR*sl* (DR*sl*) tests. This prospective cohort study enrolled 2957 high-risk DR-TB cases. We tested sputum specimens using conventional mycobacteriological and molecular tests. Gene sequencing was performed to resolve discordant results. According to the Xpert test, 33.6% of specimens were MTBC-positive and 5.1% were RR. RR specimens were further analyzed in the DR*plus* and DR*sl* tests. We identified 1 extensively drug-resistant (XDR), 8 pre-XDR, 18 simple multidrug-resistant (MDR), 22 mono-RR, and 2 RR cases with concurrent second-line injection DR-TB. Of these, 25 (49%) were relapses, 13 (25.5%) were treatment failures, 10 (19.6%) were from MDR-TB high-incidence areas/countries, 1 was from MDR-TB contact and 2 were unknown. Among culture-positive TB cases, the sensitivities, specificities, and positive predictive values (PPVs) of the Xpert test and RR cases were 73.6% and 100.0%, 85.7% and 98.6%, and 73.5% and 80.0%, respectively. Gene sequencing of discordant results revealed 7 disputed *rpoB* mutations and 2 silent mutations for RIF, 1 *ahpC* mutation for isoniazid and 1 *gyrA* mutation for fluoroquinolone. The algorithm effectively identified approximately 23% of annual MDR-/XDR-TB and 37.5% of RR-TB cases that were enrolled in our DR-TB treatment and management program within 3 days.

## Introduction

Tuberculosis (TB) is a major public health concern worldwide and a notable communicable disease in Taiwan. The World Health Organization (WHO) world TB report 2017 indicated that two-thirds (61%) of the 10.4 million new TB cases and nearly a quarter of the 490000 new cases of multidrug-resistant TB (MDR-TB), with 6.2% of those MDR-TB cases were extensively drug-resistant TB (XDR-TB), were detected and reported in 2016 [1]. However, there were gaps between the notified cases and the estimated incident cases, reflecting a mixture of underreporting of detected TB cases and under-diagnosis, representing a potential public health threat to communities.

In Taiwan, the TB incidence rate was 45.7 per 100000 in 2015 and 43 per 100000 in 2016. A drug resistance surveillance report from the Taiwan Centers for Disease Control (CDC) [2] provided the following first-line TB drug resistance ratios between new and retreated cases: isoniazid (INH) (9%, 18%), rifampin (RIF) (2%, 10%), ethambutol (EMB) (2%, 7%), streptomycin (SM) (8%, 12%) and MDR-TB (1%, 6%). Taiwan launched the “Halving TB in 10 Years Program in Taiwan 2006–2015” in 2006, and incidence and mortality rates are declining. To achieve the post-2015 End TB Strategy targets of a 50% reduction in TB incidence, 75% reduction in mortality from TB by 2025, and 90% reduction in TB incidence by 2035, more robust strategies and rapid, active intervention are needed.

Thus, the rapid diagnosis and identification of DR *Mycobacterium tuberculosis* complex (MTBC) are priorities for combatting TB. Conventional methods for mycobacterial culture and drug susceptibility testing (DST) are time-consuming and complicated, requiring prolonged procedures for diagnosis. During this period, patients may be improperly treated, and DR strains may continue to spread, resulting in the expansion of drug resistance.

Compared with conventional TB diagnostic methods, molecular techniques directly detect clinical specimens of MTBC and drug resistance-related mutations with high accuracy and efficiency and thus have become increasingly prominent in TB control strategies [3]. Commercially available nucleic acid amplification tests (NAATs) for RIF and/or INH resistance have been recommended by the WHO; these tests include the GenoType MTBDR*plus* (DR*plus*) (Hain Lifescience, Nehren, Germany) in 2008 [4] and the Xpert MTB/RIF (Xpert) (Cepheid, Sunnyvale, CA) in 2010 [5–6]. The Xpert test is a semi-nested real-time polymerase chain reaction (PCR)-based assay, which uses three primers to amplify the MTBC-specific sequence of the *rpoB* gene and five fluorescent wild-type probes to screen the 81-bp (codon 507–533) rifampicin resistance determining region (RRDR). The advantages of this test are its simplicity and its full automation, rendering it capable of simultaneously detecting MTBC and rifampicin resistance (RR) within 2 hours [5–6]. The assay has demonstrated a pooled sensitivity of 90% (98% and 67% among smear-positive and negative specimens, respectively) and specificity of 99% for the detection of MTBC. The sensitivity and specificity are 94% and 97%, respectively, for the detection of RR in respiratory samples [5, 7].

The DR*plus* test is a commercially available line-probe assay (LPA) that identifies MTBC and resistance to RIF and INH, including mutations in the 81-bp hotspot region of the *rpoB* and the *inhA* promoter region at codon 315 of the *katG* gene. This test is based on DNA-STRIP technology, comprising DNA extraction, multiplex polymerase chain reaction (PCR) with biotinylated primers, and reverse hybridization, with a turnaround time of less than 8 hours [8]. Another test, the GenoType MTBDR*sl* (DR*sl)* (v2) test, was developed for the molecular detection of resistance-conferring mutations in the *gyrA* and *gyrB* genes for fluoroquinolones (FLQs, ofloxacin, moxifloxacin, levofloxacin) and in the *rrs* and *eis* genes for second-line injectable drugs (SLIDs; amikacin, AMK; kanamycin, KAN; and capreomycin, CAP). The test demonstrates sensitivities and specificities of 84% and 100% for FLQ resistance, 86% and 90% for SLIDs and 80% and 96%, respectively, for XDR-TB when performed on isolates [9–10].

Adopting a molecular testing approach to determine TB drug resistance during the early clinical decision process is recommended in international TB control strategies. In Taiwan, a policy encouraging the rapid diagnosis of MDR-TB was adopted in the TB control program in 2006; however, this policy applies only to smear-positive high-risk populations. Subsequently, from 2010 to 2015, MDR-TB cases were confirmed by at least two smear-positive sputum samples showing RIF and INH resistance using the DR*plus* test. To further improve the utilization of molecular diagnostics for case management, we implemented and evaluated the diagnostic performance of an algorithm with the Xpert test and 2 LPAs to expand and to streamline the screening of both smear-positive and smear-negative high-risk, DR-TB populations.

## Materials and methods

According to Taiwan Communicable Disease Control Act, TB is one of the notifiable diseases and specimen collection for laboratory testing is mandatory. This study was reviewed and approval by the institutional review board of Taiwan CDC (Tw-CDC IRB-104120), and participant consent was not required.

### Study design

**Fig 1:**
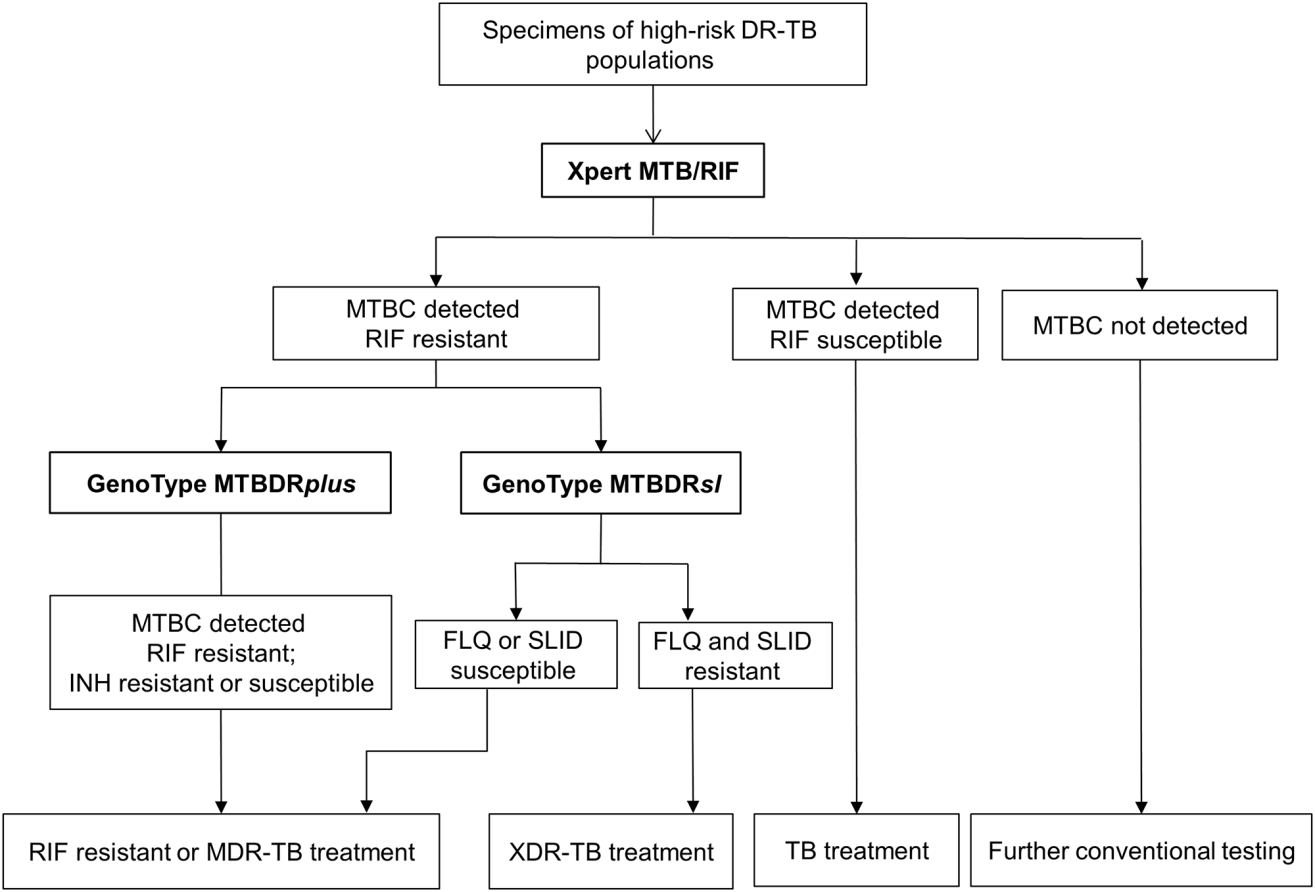
An Xpert-based algorithm for diagnosing DR-TB among high-risk populations.

From January to December 2016, we prospectively enrolled high-risk DR-TB individuals, including treatment default cases, treatment failure cases, relapse cases, presumptive cases from high-risk areas in Taiwan (Xiulin, Zhuoxi, Wanrong, and Ji’an villages in Hualien County; Dongsheng and Dunkou-Daren villages in Yunlin County; and Renai village in Nantou County), DR-TB contacts and presumptive cases who stayed in countries designated by the WHO as having a high TB or DR-TB burden for more than one month in the preceding year [1]. Specimens underwent routine acid-fast bacteria (AFB) smear microscopy, mycobacterial culture, and subsequent identification and drug susceptibility testing (DST) in authorized TB laboratories in Taiwan. Aliquots of sputum sediments after NALC-NaOH (N-acetyl L-cysteine sodium hydroxide) decontamination were sent to the reference laboratory of the Taiwan CDC for molecular testing. The diagnostic algorithm is shown in Fig 1. The Xpert test (Cepheid, Sunnyvale, CA) was used for initial diagnosis. Samples with *Mycobacterium tuberculosis* complex (MTBC)-positive and RR results were subsequently and simultaneously tested using the GenoType MTBDR*plus* (DR*plus*) and MTBDR*sl* (DR*sl*) tests. If two or more sputum specimens from one case were collected on the same day, the higher smear-grade specimens were tested. The results of conventional bacteriological tests were uploaded by authorized TB laboratories to the Infectious Disease Notification System of the Taiwan CDC.

### AFB smear microscopy

Concentrated sputum smears were prepared using the NALC-NaOH method. Microscopy with the centrifuged sediments of concentrated samples was performed using auramine O (AO) fluorescent staining and confirmed using Ziehl-Neelsen staining. Smear results were interpreted according to the guidelines issued by the American Thoracic Society [11].

### Mycobacterial culture, identification and drug susceptibility testing

Decontaminated specimens were inoculated in solid and liquid culture. Negative culture results were reported after incubation for 42 days without the isolation of any mycobacteria. The MPB64 antigen and DST of MTBC were detected as previously described [12–14]. Briefly, *M*. *tuberculosis* isolates were subjected to DST using the proportion method with 7H10 medium (Becton, Dickinson and Company, Spark, MD, USA). Drug resistance was defined as the growth of 1% of colonies in the presence of drug-containing medium. The critical concentrations of the tested drugs were: INH, 0.2 μg/ml; RIF, 1 μg/ml; SM, 2 μg/ml; EMB, 5 μg/ml; AMK, 6 μg/ml; KAN, 6 μg/ml; CAP, 10 μg/ml; ofloxacin (OFX), 2 μg/ml; moxifloxacin (MOX), 0.5 μg/ml; and levofloxacin (LVX), 1.0 μg/ml. MDR-TB is defined as an MTBC isolate that is resistant to at least INH and RIF. XDR-TB is defined as an MDR MTBC isolate that is resistant to at least one FLQ and one injectable drug, while pre-XDR-TB is defined as an MDR *M*. *tuberculosis* isolate that is resistant to either FLQ or at least one SLID.

### The Xpert MTB/RIF test

The Xpert test was performed according to the manufacturer’s instructions [15]. Briefly, sample reagent was added to a 500-μl sputum sample at a 3:1 ratio in a 15-ml centrifuge tube and incubated at room temperature for 15 minutes. During the incubation period, the samples were mixed by inverting the tubes gently 2 times every 5 minutes. Then, 2.0 ml of liquefied sample was transferred to an Xpert cartridge (G4 version) and loaded into the GeneXpert machine. Results were available within two hours of sample loading.

### The GenoType MTBDR*plus* and GenoType MTBDR*sl* tests

The DR*plus* (v2) and DR*sl* (v2) (Hain Lifesciences GmbH, Nehren, Germany) tests were performed with the RR sputum samples detected by the Xpert test to detect mutations involved in resistance to RIF, INH, FLQs and SLIDs, according to the manufacturer’s instructions [16–17].

### Discordant analysis

DNA sequencing of drug resistance-conferring genes was performed for discordant analyses between tests. The *rpoB* gene was amplified with the primers *rpoB*-F (5’-TCG GCG AGC CCA TCACGT CG-3’) and *rpoB*-R (5’-GCG TAC ACC GAC AGC GAG CC-3’), which yielded a 541-bp fragment containing the hotspot region. To detect INH resistance, *katG* and the *inhA* locus (*inhA* regulatory region) were amplified with the following primers: *katG*-F (5’-GTC ACA CTT TCG GTA AGA C-3’) and *katG*-R (5’-TTG TCG CTA CCACGG AAC G-3’); and *inhA* locus-F (5’-AAT TGC GCG GTC AGT TCC AC-3’), *inhA* locus-R (5’-GTC GGT GAC GTC ACA TTC GA-3’), *ahpC*-F (5’-GCT TGA TGT CGG AGA GCA TCG-3’), and *ahpC*-R (5’-GGT CGC GTA GGC AGT GCC CC-3’). To detect FLQ resistance and AG/CP resistance, the *gyrA* gene and the *rrs* gene were analyzed with the following primers: *gyrA*-F (5’-GAT GAC AGA CAC GAC GTT GC-3’) and *gyrA*-R (5’-AGC ATC TCC ATC GCC AAC G-3’); and TBrrs1250-F (5’-TTA AAA GCC GGT CTC AGT TC-3’) and TBrrivs38-R (5’-TAC GCC CCA CCA GTT GGG GC-3’). PCR was performed as follows: 35 cycles at 95°C for 1 min; annealing for 1 min at 64°C for *rpoB*, 55°C for *katG*, 60°C for *ahpC* and 65°C for *gyrA* and *rrs*; and elongation at 72°C for 1 min. Then, the PCR products were analyzed with an ABI 3730 automated sequencer (Applied Biosystems), and the sequence data were assembled and edited using sequencing analysis (version 5.2.0) software (Applied Biosystems) [18].

### Statistical analysis

Statistical analysis was carried out using the SPSS 24.0 software package (IBM, Armonk, New York, USA). Multiple comparisons of the percentages between pair-wise groups were performed using the two-proportional-Z-test with the Bonferroni correction. Sensitivity, specificity, positive predictive value (PPV), negative predictive value (NPV), and accuracy were calculated for the detection of MTBC and drug resistance using culture and phenotypic DST as the reference standards, respectively, with exact Clopper-Pearson 95% confidence intervals. All hypothesis tests were two-sided with a significance level of 0.05.

## Results

### Study populations

We enrolled 2957 high-risk DR-TB cases in 2016. Table 1 lists the specimens collected from high-risk populations, including countries with a high TB and MDR-TB burden (32.9%, 973/2957), relapse (29.6%, 876/2957) and treatment failure (18.4%, 544/2957) populations. The median age of cases at the time of specimen collection was 55 years (interquartile range: 35.8 to 70.0 years). Most cases were male (65.6%, 1939/2957). Of these, 97% (2867/2957) of cases had AFB smear results, including 57.9% smear-negative cases and 6.6%, 18.6%, 6.6%, 3.7%, and 3.7% cases that were scanty, 1+, 2+, 3+, and 4+, respectively. Of the 2957 specimens tested, we identified 958 (32.4%) specimens that were MTBC culture-positive, 1532 (51.8%) that were culture-negative for mycobacteria, 240 (8.1%) that were nontuberculous mycobacteria (NTM) and 227 (7.7%) that had contaminated or no culture results (Table 2). Phenotypic DST results were available for MTBC culture-positive specimens with the following resistance ratios: 4.7% for RIF, 12.4% for INH, 9.4% for SM, and 3.7% for EMB. Additionally, 3.2% was MDR, 0.7% was pre-XDR and 0.1% was XDR.

**Table 1:** Xpert and LPA results for high-risk DR populations.

Group	MTBC, % (positive/total case no.)	Drug resistance						
		RIF-R*	RIF-R & SLID-R	Simple MDR	Pre-XDR (FLQ-R)	Pre-XDR (SLID-R)	XDR	Total
Treatment default	28.8 (17/59)	0	0	0	0	0	0	0
Treatment failure	58.5 (318/544)	5	2	3	0	3	0	13
Relapse	31.7 (278/876)	10	0	13	1	1	0	25
Close contacts with DR-TB patients	34.4 (11/32)	0	0	1	0	0	0	1
Presumptive TB from high-risk area in Taiwan	37.1 (118/318)	2	0	1	0	0	0	3
Presumptive TB from TB or DR-TB high-burden countries	20.3 (198/973)	3	0	0	1	2	1	7
Unknown	34.2 (53/155)	2	0	0	0	0	0	2
Total	33.6% (993/2957)	22	2	18	2	6	1	51

Abbreviations: DR, drug resistance; MTBC, *Mycobacterium tuberculosis* complex; RIF-R, rifampin resistant; FLQ-R, fluoroquinolone resistant; SLID-R, second-line injectable drug rifampin resistant; MDR, multidrug-resistant tuberculosis; XDR, extensively drug-resistant tuberculosis

* Due to the low sample volumes, these four cases, which included the treatment failure, relapse, high-burden countries and unknown groups, were identified by the Xpert test as RIF-resistant but were not further tested using the LPAs.

**Table 2:** Performance of the Xpert test for the detection of MTBC, compared to smear and culture results.

		Culture results, no.				Performance %, (95% CI)			
		MTBC		NTM	Total	Sensitivity	Specificity	PPV	NPV
		Positive	Negative						
Xpert results, n = 2726*									
MTBC detected		704	211	43	958	73.6	85.7	73.5	85.8
MTBC not detected		252	1319	197	1768	(70.7–76.4)	(83.9–87.3)	(71.1–75.8)	(84.4–87.0)
Total		956	1530	240	2726				
**AFB smear results, n = 2667**^#^									
Smear-positive		584	360	151	1095	63.0	70.6	55.3	78.2
Smear-negative		343	1154	75	1572	(59.8–66.1)	(68.4–72.8)	(51.1–55.5)	(76.6–79.7)
Total		927	1514	226	2667				
**Xpert results stratified by AFB smear results**									
Smear-positive	Xpert (+)	552	150	35	737	94.5	63.8	74.9	91.1
	Xpert (-)	32	210	116	358	(92.4–96.2)	(59.5–68.0)	(72.6–77.0)	(87.9–93.5)
	Total	584	360	151	1095				
Smear-negative	Xpert (+)	132	52	6	190	38.7	95.5	69.5	84.8
	Xpert (-)	209	1100	69	1378	(33.5–44.1)	(93.9–96.4)	(63.1–75.2)	(83.7–85.9)
	Total	341	1152	75	1568				

Abbreviations: MTBC, *Mycobacterium tuberculosis* complex; NTM, nontuberculous mycobacteria

* Of 2957 tested specimens, 231 were excluded from the analysis due to missing culture results and MTBC-indeterminate results.

^**#**^ Sixty-three cases were excluded from the analysis due to missing smear results.

### Detection of drug resistance using a designed algorithm

Of the 2957 specimens tested using the Xpert test, 33.6% (993/2957) were MTBC-positive and 5.1% (51/993) were RR. The group from countries with a high TB and MDR-TB burden had a significantly lower detection rate than those of the treatment failure group, relapse group, high-risk area group and MDR-TB contact group. The treatment failure group had the highest TB detection rate, 58.5% (318/544), among all groups (p < 0.05). Excluding 4 specimens with low sample volumes, 47 RR specimens were simultaneously further analyzed using the DR*plus* and DR*sl* tests. We identified 18 mono-RR, 2 RR concurrently resistant to SLID, 18 simple-MDR, 8 pre-XDR (2 resistant to FLQ and 6 resistant to SLIDs) and 1 XDR-TB. Of the 47 RR cases analyzed, 51.1% (24/47) and 25.5% (12/47) were relapse and treatment failure cases, respectively (Table 1). The reported test results had a median of 2 days (range: 1–4 days) and an average of 2.23 days.

### Performance of the Xpert test and LPAs using conventional results as references

Of the 2957 specimens, we excluded 4 specimens with indeterminate results, 14 contaminated specimens and 213 specimens with missing data in our analyses. We used conventional MTBC culture results as references. The sensitivity and specificity of AFB smear microscopy were 63.1% and 70.6%, respectively. Xpert test performance showed an overall sensitivity of 73.6%, including 94.5% sensitivity for AFB smear-positive and 38.7% sensitivity for AFB smear-negative specimens; the specificity was 85.7%. The Xpert test detected 38.7% (132/341) of MTBC as smear-negative culture-positive specimens and excluded 63.8% (326/511) of smear-positive culture-negative specimens. The Xpert test also correctly excluded 82.1% (197/240) of NTM specimens, including 76.8% (116/151) misdiagnosed as MTBC by smear microscopy alone (Table 2).

Of the 704 specimens with Xpert test results, we excluded 2 specimens with missing DST results and 5 specimens with RIF-indeterminate results. Of the remaining 697 Xpert-MTBC test results, the sensitivity of the Xpert test for RR detection was 100.0% (90.3–100.0%), and the specificity was 98.6% (97.4–99.4%), with a PPV of 80.0% (67.6–88.4%) and an NPV of 100.0% (99.4–100.0%) (Table 3).

**Table 3:** Xpert and LPA results for drug resistance detection, compared to conventional DST results.

		Conventional DST results, no.			Performance, %			
		Resistant	Susceptible	Total	Sensitivity	Specificity	PPV	NPV
Xpert results, n = 697*								
RIF	Resistant	36	9	45	100	98.6	80.0	100.0
	Susceptible	0	652	652	(90.3–100.0)	(97.4–99.4)	(67.6–88.4)	(99.4–100.0)
	Total	36	661	697				
**LPA results, n = 44**^#^								
RIF	Resistant	36	8	44				
	Susceptible	0	0	0				
	Total	36	8	44				
INH	Resistant	26	0	26	96.3	100.0	100.0	94.4
	Susceptible	1	17	18	(81.0–99.9)	(80.5–100.0)	(87.1–100.0)	(71.3–99.2)
	Total	27	17	44				
MDR	Yes	24	2	26	96.0	89.5	92.3	94.4
	No	1	17	18	(79.7–99.9)	(66.9–98.7)	(76.3–97.8)	(71.2–99.2)
	Total	25	19	44				
Pre-XDR/XDR	Yes	6	3	9	85.7	91.9	66.7	97.1
	No	1	34	35	(47.4–99.7)	(73.1–98.3)	(39.3–86.1)	(84.7–99.5)
	Total	7	37	44				

Abbreviations: LPA, line probe assay; DST, drug-susceptibility testing; MTBC, *Mycobacterium tuberculosis* complex; RIF, rifampin; INH, isoniazid; MDR, multidrug-resistant tuberculosis; XDR, extensively drug-resistant tuberculosis; PPV: positive predictive value; NPV, negative predictive value

* Of the 704 specimens identified as MTBC by the Xpert and culture methods, 7 were excluded from the analysis due to missing DST results and RIF-indeterminate results.

^#^ Of the 51 specimens identified as RIF-resistant by Xpert assay, four of which failed to detect line probe assays due to low sample volume, conventional DST results were available for 44 specimens in total for comparison.

Of the 44 specimens that had paired conventional DST and LPA results, 97.7% (43/44) had concordant INH-resistant results, whereas 1 specimen was detected as INH-susceptible by the DR*plus* test. There was 93.2% agreement between the DRs*l* test and phenotypic DST results for MDR-TB detection, with 3 discordant results, and 90.9% agreement for pre-XDR/XDR detection, with 5 discordant results (Table 3).

### Probe hybridization patterns of the Xpert test and LPAs

Excluding 4 specimens with low sample volumes for LPA analysis, the frequency of mutations associated with RR is summarized in Table 4. Of the 51 specimens detected as RR by the Xpert test, there were 7 with probe A failure, 7 with probe B failure, 1 with probe C failure, 11 with probe D failure, and 25 with probe E failure, resulting in probe failure rates of 13.7%, 13.7%, 2.0%, 21.6%, and 49.0%, respectively. In addition, 23 (48.9%) specimens were missing wild type (WT) 8 (530–533) and MUT3 binding (S531L) in the *rpoB* gene, which was a predominant mutation associated with RR (Table 4). There were no significant differences in the frequencies of Xpert probe mutations in different high-risk populations.

**Table 4:** Xpert and LPA probe hybridization patterns.

Xpert probe failure (mutation)	No. of cases (%)	DR*plus* probe WT absent, MUT binding (mutation)	No. of cases (%)
A (507–511)	7 (13.7)	*rpoB* WT 1/WT 2 (505–513)	5 (10.6)
B (511–518)	7 (13.7)	*rpoB* WT 3/4, MUT 1 (513–519, D516V)	7 (14.9)
C (518–523)	1 (2.0)	*rpoB* WT 5/6 (518–525)	1 (2.1)
D (522–528)	11 (21.6)	*rpoB* WT 7, MUT 2A or 2B (526–529, H526Y or H526D)	11 (23.4)
E (528–533)	25 (49.0)	*rpoB* WT 8, MUT3 (530–533, S531L)	23 (48.9)
Total	51 (100)	Total	47 *(100)
		*katG* WT, MUT1 (S315T)	17 (63.0)
		*inhA* WT 1, MUT1 (C15T)	6 (22.2)
		*inhA* WT 2, MUT 3A (T8C)	1 (3.7)
		*katG* WT, MUT 1 and *inhA* WT 1, MUT 1 (S315T and C15T)	3 (11.1)
		Total	27 (100)
		DR*sl* probe WT absent, MUT binding (mutation)	No. of cases (%)
		*gyrA* WT 2, MUT 1 (A90V)	1 (9.0)
		*gyrA* WT 3, MUT 3C (D94G)	1 (9.0)
		*rrs* WT 1, MUT 1 (A1401G)	3 (27.3)
		*rrs* MUT 2 (G1484T)	3 (27.3)
		*rrs* WT1, WT2, MUT 2 (G1484T)	2 (18.2)
		*gyrA* WT 2, MUT 1 and *rrs* WT 1, MUT 1 (A90V and A1401G)	1 (9.0)
		Total	11 (100)

Abbreviations: LPA, line probe assay; DR*plus*, GenoType MTBDR*plus*; DR*sl*, GenoType MTBDR*sl*

* Four samples with low volumes were excluded for the LPAs.

The DR*plus* test detected 27 of 47 specimens as INH-resistant. The mutation patterns conferring INH resistance included *katG* S315T1 (17/27), *inhA* MUT1 (C15T, 6/27), MUT3A (T8C, 1/27), and *katG* S315T1 and *inhA* C15T double mutations (3/27) (Table 4). The *katG* S315T mutation, conferring high-level INH resistance, was the predominant mutation (17/27, 63.0%) among the tested specimens but was not associated with screening groups.

The DR*sl* test identified 11 of 47 specimens with mutations conferring FLQ resistance and/or SLID resistance. The following mutation patterns were identified: *gyrA* MUT1 (A90V, 1/11); *gyrA* MUT3C (D94G, 1/11); *rrs* MUT1 (A1401G, 3/11); *rrs* WT present and MUT2 (G1484T, 3/11); *rrs* MUT2 (G1484T, 2/11); both mutations of *gyrA* MUT1; and *rrs* MUT1 (A90V and A1401G, 1/11) (Table 4). One case was classified as XDR with the *katG* S315T, *inhA* C15T, *gyrA* A90V and *rrs* A1401G mutations.

### Analysis of discordant results

Nine specimens were identified as RR by the Xpert test but susceptible by phenotypic DST. We sequenced the RRDR for 9 specimens, and mutations were found in codons G507G (1/10), L511P (3/10), L521L (1/10), D516Y (1/10), H526N (1/10) and L533P (2/10), as well as two silent mutations.

One case was INH-susceptible according to the DR*plus* test and gene sequencing but resistant according to phenotypic DST; sequencing results revealed there was a mutation in the *ahpC* gene. For MDR detection, 3 cases showed inconsistent results due to 2 RIF (*rpoB* L533P, L511P) and 1 INH discrepancy between molecular and phenotypic DST results.

Of the 5 cases with inconsistent pre-XDR detection results, one case was susceptible to SLIDs according to both the DR*sl* test and gene sequencing but resistant to CAP according to phenotypic DST. One phenotypic FLQ-susceptible case harboring the *gyrA* A90V mutation was resistant according to both DR*sl* (*gyrA* MUT1) and gene sequencing. Nevertheless, 3 cases resistant to SLIDs (*rrs* WT present and MUT2) in the DR*sl* test were susceptible according to the phenotypic DST and *rrs* gene sequencing results.

## Discussion

Adopting and implementing WHO TB policies and guidelines is fundamental and essential to ending TB by 2030. Of approximately 120 annual registered MDR-TB cases, 70–80 (60%) were new MDR cases in Taiwan. The Taiwan CDC implemented a directly observed treatment, short-course (DOTS-Plus) care program for the management of MDR-TB in 2007 [19–21] and RR-TB in 2011; however, the effectiveness of the program may be hampered by laboratory diagnosis because conventional TB diagnostics are time-consuming and resource-intensive. Screening high-risk populations with new molecular tests would significantly reduce the cost of the program. In this study, we implemented a designed algorithm with the Xpert test and 2 LPAs in the TB diagnostic system to strengthen its capacity. We identified approximately 23% of MDR-TB and 37.5% of RR-TB cases enrolled in our DR-TB treatment and management program within 3 days. This study reinforced the new Taiwan CDC policy to confirm RR- and MDR-TB cases because one case with a sputum sample detected by the Xpert test as MTBC and RR and confirmed by the DR*plus* test to be RIF- and INH-resistant was promptly provided second-line drugs. In addition, this study strengthened TB laboratory capacity for timely detection of preXDR- and XDR-TB in 2016.

Several multicenter studies and meta-analyses have validated the clinical performance and excellent accuracy of the Xpert test for the diagnosis of pulmonary TB and RR, regardless of the TB incidence and resources of the implicated countries [5,7,22–24]. In the present study, we evaluated an algorithm for the Xpert test to use for countrywide screening of high-risk DR-TB populations. Of the 252 Xpert-negative MTBC culture-positive specimens, 220 (87.3%) were smear-negative or scanty, which may be due to the low bacillary load, given that the limit of detection (LOD) for sputum samples (131 cfu/ml) in the Xpert test is higher than the LOD for culture samples (100 cfu/ml) [24,25]. Alternatively, the Xpert test detected a substantial number of cases missed by culture, of which the majority were from previously treated patients (217/254) and potentially contained non-viable MTBC bacilli. Other possibilities include over-decontamination or mixing with NTM, leading to negative results or misclassification of the NTM results. Compared with AFB smear microscopy, the Xpert test had a higher sensitivity for MTBC detection in smear-positive cases than in smear-negative cases and showed a 40% increase in TB detection among culture-confirmed cases. Although smear-negative cases are less infectious, they may account for up to one-fifth of all secondary transmission [26–27]. Furthermore, the Xpert test correctly excluded 82.1% of cases with NTM isolation, which may reduce the burden of contact tracing and case management and avoid unnecessary latent TB infection diagnosis and treatment. Therefore, this test is valuable as an initial test replacing AFB smear microscopy for screening high-risk DR-TB cases in Taiwan (intermediate TB burden, high resource setting). For RR detection, the Xpert test provides accurate results and allows the rapid initiation of MDR-TB treatment within 1–3 days depending on logistics.

Both the Xpert and DR*plus* tests target the same *rpoB* RRDR region for the detection of RR-associated mutations. Our results did show concordant patterns of Xpert probe failure and WT probe absence or the presence of MUT probes in the DR*plus* test. The mutation at codon 531 covered by probe E in the Xpert test and MUT3 in the DR*plus* test was the most frequent (48.9%), followed by codon 526 covered by probe D and WT7/MUT2A/2B (23.4%) and codon 516 covered by probe B and MUT1 (14.9%). These findings are consistent with other studies [28–30].

Nine samples were identified as RR by the Xpert test and susceptible by the phenotypic DST. Two of these nine samples contained silent mutations according to gene sequencing, while the other 7 specimens were associated with disputed mutations (L511P, D516Y, H526N and L533P) contributing to low-level RR [31]. Recent studies reported that disputed mutations comprise 11–13% of all *rpoB* mutations in previously treated patients and account for 9% of isolates with genotypic RR but phenotypic susceptibility [32–33]. Therapies for patients with low-level RR are challenging because these cases are phenotypically susceptible to RIF; however, patients often relapse or experience treatment failure [34–35]. In our study, we found that 6 out of 7 disputed mutations were derived from cases with treatment failure or relapse. Therefore, for cases identified as RR using the Xpert test in our screened populations, in the absence of pending culture and phenotypic DST results, treatment options other than standard first-line anti-TB regimens should be considered, particularly in the presence of disputed mutations.

Additionally, phenotypic DST revealed one INH-resistant case and one CAP-resistant case that had no resistance-conferring mutation according to the LPAs and *katG* and *inhA* gene sequencing. Since mutations in genes other than the *katG* and *inhA* genes occur in 2–10% of all INH-resistant MTBC strains [36–37], we identified *aphC* C-15T in the discordant INH-resistant strain; approximately 7.4% of MDR isolates in Taiwan have a mutation in the *ahpC* gene [18]. Furthermore, the *rrs* A1401G mutation is the predominant mutation conferring resistance to KAN (60%), AMK (75%), and/or CAP (75%) [38]. Phenotypic resistance to SLIDs is associated with cumulative mutations in diversified genes lacking corresponding mutation probes, which were included in the DR*sl* test [38]. The LPAs target only a limited number of resistance variants and do not identify all gene mutations conferring resistance to anti-TB drugs [16–17].

One case was resistant to FLQs (moxifloxacin and levofloxacin) via the *gyrA* A90V mutation according to both the DR*sl* test and gene sequencing but was susceptible according to the phenotypic DST results. Minimal inhibitory concentration (MIC) testing suggests that phenotypic DST might underestimate the true rates of levofloxacin and moxifloxacin resistance because critical concentrations defined by the WHO are too high and must be reexamined to avoid systematic misclassification in isolates with low-level resistance [39].

The 3 other treatment failure cases were resistant to SLIDs in the DR*sl* test, showing mixed patterns, but susceptible according to phenotypic DST and gene sequencing. This discrepancy might be explained by the fact that gene sequencing uses cultured MTBC isolates while the DR*sl* test is performed using sputa samples, and relatively abundant heterogeneity of individual alleles persists during treatment [40]. Expansion and selection of the MTBC population during bacterial culture prior to sequencing is likely to influence the performance of molecular assays and phenotypic DST and must be considered when interpreting diagnostic results.

The turnaround time for the molecular detection of DR-TB (including MDR-/XDR-TB) had a median of 2 days (range: 1–4 days) and an average of 2.23 days, compared to 6 weeks for MDR-TB and 10 weeks for XDR-TB using conventional tests. The WHO conditionally recommended a standard short 9–12-month regimen for MDR-TB in 2016 [41]. Confirmed resistance or suspected ineffectiveness of a drug is one of the exclusion criteria for the shorter MDR-TB regimen. The initiation of appropriate treatment for DR-TB patients and interrupted transmission must rely on reliable and rapid DST. The introduction of rapid molecular diagnostic tests in Taiwan, notably the Xpert test combined with 2 LPAs, for the detection of DR-TB has markedly improved case detection and management with reduced diagnosis-to-treatment time.

In summary, to improve the utilization of molecular diagnostics for case management, we implemented an algorithm to intensify DR-TB detection and to exclude cases with NTM isolation. We proved that the use of Xpert as an initial test for the rapid detection of RR, followed by two LPAs simultaneously, was more effective than phenotypic culture-based DST to detect INH and second-line DR in confirmed RR and MDR cases. Few false-resistance (mutations not conferring resistance) and false-susceptibility (mutations different from common mechanisms mediating resistance designed in the molecular tests) cases were observed in our study; however, the algorithm prompted and accurately detected most DR-TB cases for optimal treatment within 3 days.

## Supporting information

S1 File Minimal dataset. (PDF)
