# Supplementary material for: Performance of an Xpert-based diagnostic algorithm for the rapid detection of drug-resistant tuberculosis among high-risk populations in a low-incidence setting
Source: PLoS One. 2018 Jul 16;13(7):e0200755. doi: 10.1371/journal.pone.0200755 (PMC6047812; doi:10.1371/journal.pone.0200755)
Supplement: S1 File — (PDF) [file pone.0200755.s001.pdf]

Pt#	Group	AFS	Xpert			DR*plus* (WT absent, MUT binding)		DR*sl* (WT absent, MUT binding)		Sequencing (Discordant)				Convetional results						
			MTBC	RIF	Probe Failure	RIF	INH	FLQ	SLID	RIF	INH	FLQ	SLID	Cul.	INH	RIF	FLQ	KAN	AMK	CAP
1	failure	S	Pos	S										Neg						
2	high-risk	1+	Neg											NTM						
3	high-risk	1+	Neg											NTM						
4	failure	1+	Neg											Neg						
5	relapse	1+	Pos	S										NTM						
6	high-burden	4+	Pos	S										Pos	S	S				
7	failure	1+	Neg											Neg						
8	failure	S	Pos	S										Pos	R	S				
9	relapse	S	Neg											Neg						
10	high-risk	1+	Pos	S										Pos	S	S				
11	failure	S	Neg											Neg						
12	high-burden	1+	Neg											Pos	S	S				
13	relapse	1+	Pos	S										Pos	S	S				
14	relapse	Neg	Neg											Neg						
15	high-burden	S	Pos	S										Pos	S	S				
16	failure	1+	Pos	S										Neg						
17	failure	S	Pos	S										Pos	S	S				
18	failure	2+	Pos	S										Pos	S	S				
19	relapse	S	Pos	S										Pos	S	S				
20	relapse	Neg	Neg											Neg						
21	failure	Neg	Neg											Neg						
22	high-burden	Neg	Neg											Neg						
23	failure	S	Pos	S										Pos	S	S				
24	failure	1+	Pos	S										Neg						
25	failure	1+	Pos	S										Neg						
26	relapse	Neg	Neg											Neg						
27	high-burden	Neg	Neg											Neg						

Pt#	Group	AFS	Xpert			DR*plus* (WT absent, MUT binding)		DR*sl* (WT absent, MUT binding)		Sequencing (Discordant)				Convetional results						
			MTBC	RIF	Probe Failure	RIF	INH	FLQ	SLID	RIF	INH	FLQ	SLID	Cul.	INH	RIF	FLQ	KAN	AMK	CAP
28	relapse	Neg	Neg											Neg						
29	failure	1+	Pos	S										Pos	S	S				
30	relapse	1+	Neg											Neg						
31	default	S	Pos	S										Pos	S	S				
32	high-burden	S	Neg											Neg						
33	relapse	Neg	Pos	S										Pos	S	S				
34	failure	Neg	Pos	S										Neg						
35	high-burden	Neg	Neg											Neg						
36	relapse	Neg	Neg											Neg						
37	high-risk	Neg	Neg											Neg						
38	failure	2+	Neg											Neg						
39	failure	S	Pos	S										Neg						
40	relapse	Neg	Neg											Pos	S	S				
41	high-burden	Neg	Pos	S										Pos	S	S				
42	relapse	1+	Pos	S										Pos	S	S				
43	relapse	S	Neg											Neg						
44	high-burden	1+	Pos	S										Neg						
45	relapse	Neg	Pos	S										Neg						
46	relapse	2+	Pos	S										Pos	S	S				
47	high-burden	Neg	Neg											Neg						
48	high-risk	S	Pos	S										Pos	S	S				
49	high-burden	S	Neg											Neg						
50	failure	1+	Pos	S										Pos	S	S				
51	relapse	Neg	Neg											Neg						
52	high-burden	Neg	Neg											Neg						
53	high-risk	1+	Neg											Pos	S	S				
54	high-burden	Neg	Neg											Neg						
55	relapse	1+	Pos	S										Neg						

Pt#	Group	AFS	Xpert			DR*plus* (WT absent, MUT binding)		DR*sl* (WT absent, MUT binding)		Sequencing (Discordant)				Convetional results						
			MTBC	RIF	Probe Failure	RIF	INH	FLQ	SLID	RIF	INH	FLQ	SLID	Cul.	INH	RIF	FLQ	KAN	AMK	CAP
56	relapse	Neg	Pos	S										Pos	S	S				
57	high-risk	4+	Pos	S										Pos	S	S				
58	high-risk	Neg	Neg											Neg						
59	relapse	1+	Pos	S										Neg						
60	high-risk	Neg	Neg											Neg						
61	relapse	Neg	Neg											Neg						
62	high-burden	3+	Pos	S										Pos	S	S				
63	failure	1+	Pos	S										Neg						
64	failure	Neg	Neg											Neg						
65	failure	Neg	Pos	S										Neg						
66	relapse	Neg	Neg											Pos	R	R	S	S	S	S
67	failure	2+	Pos	S										Pos	S	S				
68	failure	Neg	Neg											Neg						
69	high-risk	Neg	Neg											Neg						
70	high-burden	1+	Neg											Pos	S	S				
71	failure	Neg	Pos	S										Pos	S	S				
72	high-risk	Neg	Neg											Neg						
73	relapse	Neg	Neg											Neg						
74	failure	1+	Pos	S										Pos	S	S				
75	relapse	Neg	Neg											NTM						
76	failure	4+	Neg											NTM						
77	failure	1+	Pos	S										Pos	S	S				
78	relapse	Neg	Pos	S										Neg						
79	default	Neg	Neg											Neg						
80	failure	1+	Neg											Neg						
81	failure	1+	Neg											Neg						
82	relapse	Neg	Neg											Neg						
83	failure	Neg	Neg											NTM						

Pt#	Group	AFS	Xpert			DR*plus* (WT absent, MUT binding)		DR*sl* (WT absent, MUT binding)		Sequencing (Discordant)				Convetional results						
			MTBC	RIF	Probe Failure	RIF	INH	FLQ	SLID	RIF	INH	FLQ	SLID	Cul.	INH	RIF	FLQ	KAN	AMK	CAP
84	relapse	Neg	Neg											Neg						
85	high-risk	Neg	Neg											Neg						
86	high-risk	Neg	Neg											Neg						
87	close contacts	Neg	Neg											Neg						
88	relapse	Neg	Neg											Neg						
89	relapse	Neg	Neg											Neg						
90	failure	Neg	Pos	S										Neg						
91	failure	2+	Pos	S										Pos	S	S				
92	failure	1+	Pos	S										Neg						
93	failure	1+	Neg											Neg						
94	failure	1+	Pos	S										Neg						
95	high-risk	4+	Pos	S										Pos	S	S				
96	high-risk	2+	Pos	S										Pos	R	S				
97	high-risk	4+	Neg											Neg						
98	high-risk	4+	Pos	S										Pos	S	S				
99	relapse	Neg	Neg											Neg						
100	failure	1+	Pos	S										Pos	S	S				
101	high-risk	Neg	Neg											Neg						
102	high-risk	Neg	Neg											Pos	S	S				
103	high-burden	1+	Neg											Neg						
104	high-burden	Neg	Neg											Neg						
105	relapse	Neg	Pos	S										Pos	S	S				
106	failure	2+	Neg											Neg						
107	high-burden	Neg	Neg											Neg						
108	failure	1+	Pos	S										Neg						
109	failure	1+	Pos	S										Neg						
110	failure	S	Pos	R	E	WT8	S		*rrs* MUT2	L533P			*rrs* WT eisWT	Pos	S	S	S	S	-	S

Pt#	Group	AFS	Xpert			DR*plus* (WT absent, MUT binding)		DR*sl* (WT absent, MUT binding)		Sequencing (Discordant)				Convetional results						
			MTBC	RIF	Probe Failure	RIF	INH	FLQ	SLID	RIF	INH	FLQ	SLID	Cul.	INH	RIF	FLQ	KAN	AMK	CAP
111	relapse	1+	Neg											Neg						
112	high-risk	Neg	Neg											Neg						
113	high-risk	2+	Neg											NTM						
114	failure	Neg	Pos	S										Neg						
115	high-risk	Neg	Neg											Neg						
116	high-risk	2+	Pos	S										Pos	S	S				
117	high-risk	1+	Neg											Neg						
118	high-burden	Neg	Neg											Neg						
119	high-burden	Neg	Neg											Neg						
120	default	Neg	Neg											Neg						
121	relapse	Neg	Pos	S										Neg						
122	failure	2+	Pos	S										Neg						
123	failure	Neg	Neg											NTM						
124	relapse	3+	Pos	S										Pos	S	S				
125	failure	S	Pos	S										Neg						
126	failure	4+	Pos	S										Pos	S	S				
127	relapse	Neg	Neg											Neg						
128	high-burden	Neg	Neg											Neg						
129	failure	1+	Neg											Neg						
130	high-burden	Neg	Neg											Neg						
131	high-burden	Neg	Neg											Neg						
132	high-risk	Neg	Neg											Neg						
133	relapse	Neg	Neg											Neg						
134	high-burden	Neg	Neg											Pos	-	-				
135	failure	S	Pos	S										Pos	S	S				
136	high-burden	Neg	Neg											Neg						
137	high-burden	Neg	Neg											Neg						
138	relapse	Neg	Neg											Neg						

Pt#	Group	AFS	Xpert			DR*plus* (WT absent, MUT binding)		DR*sl* (WT absent, MUT binding)		Sequencing (Discordant)				Convetional results						
			MTBC	RIF	Probe Failure	RIF	INH	FLQ	SLID	RIF	INH	FLQ	SLID	Cul.	INH	RIF	FLQ	KAN	AMK	CAP
139	relapse	Neg	Neg											Pos	S	S				
140	relapse	S	Pos	S										Pos	S	S				
141	high-risk	Neg	Neg											Neg						
142	high-risk	Neg	Neg											Neg						
143	high-risk	Neg	Neg											Neg						
144	high-risk	Neg	Neg											Neg						
145	high-risk	Neg	Neg											Neg						
146	high-risk	Neg	Neg											NTM						
147	relapse	Neg	Neg											Neg						
148	relapse	Neg	Neg											Neg						
149	relapse	Neg	Neg											NTM						
150	high-burden	Neg	Neg											Neg						
151	default	Neg	Pos	S										Neg						
152	relapse	Neg	Neg											NTM						
153	relapse	Neg	Neg											Neg						
154	relapse	Neg	Neg											Neg						
155	failure	1+	Pos	S										Pos	R	S				
156	relapse	Neg	Neg											Neg						
157	relapse	Neg	Neg											Neg						
158	failure	Neg	Pos	S										Pos	S	S				
159	high-burden	1+	Pos	R	E	WT8,MUT3	*katG* WT,MUT1	*gyrA* WT3,MUT3C						Pos	R	R	R	S	S	S
160	relapse	1+	Neg											Neg						
161	high-burden	Neg	Neg											Neg						
162	high-risk	Neg	Neg											Neg						
163	high-risk	2+	Pos	S										Pos	S	S				
164	default	Neg	Neg											Neg						
165	high-risk	2+	Neg											Neg						

Pt#	Group	AFS	Xpert			DR*plus* (WT absent, MUT binding)		DR*sl* (WT absent, MUT binding)		Sequencing (Discordant)				Convetional results						
			MTBC	RIF	Probe Failure	RIF	INH	FLQ	SLID	RIF	INH	FLQ	SLID	Cul.	INH	RIF	FLQ	KAN	AMK	CAP
166	high-burden	Neg	Neg											Neg						
167	high-burden	Neg	Neg											Neg						
168	relapse	Neg	Neg											Neg						
169	relapse	Neg	Neg											Neg						
170	high-risk	2+	Pos	S										Pos	S	S				
171	failure	1+	Pos	S										Pos	S	S				
172	relapse	Neg	Neg											NTM						
173	high-risk	Neg	Neg											Neg						
174	relapse	Neg	Neg											Neg						
175	high-burden	Neg	Neg											Neg						
176	failure	Neg	Pos	S										Neg						
177	high-burden	Neg	Neg											Neg						
178	relapse	Neg	Neg											Neg						
179	relapse	Neg	Neg											Neg						
180	failure	S	Pos	S										Neg						
181	high-burden	Neg	Neg											Neg						
182	high-burden	Neg	Neg											Neg						
183	high-burden	Neg	Neg											Neg						
184	relapse	Neg	Neg											Neg						
185	high-risk	Neg	Neg											Neg						
186	high-risk	Neg	Neg											Neg						
187	high-risk	Neg	Neg											Neg						
188	high-risk	Neg	Neg											Neg						
189	high-risk	Neg	Neg											Neg						
190	high-risk	4+	Neg											Neg						
191	default	Neg	Neg											Neg						
192	relapse	Neg	Neg											Pos	S	S				
193	failure	S	Neg											Neg						

Pt#	Group	AFS	Xpert			DR*plus* (WT absent, MUT binding)		DR*sl* (WT absent, MUT binding)		Sequencing (Discordant)				Convetional results						
			MTBC	RIF	Probe Failure	RIF	INH	FLQ	SLID	RIF	INH	FLQ	SLID	Cul.	INH	RIF	FLQ	KAN	AMK	CAP
194	relapse	Neg	Neg											Neg						
195	relapse	1+	Neg											NTM						
196	relapse	Neg	Neg											Neg						
197	high-risk	Neg	Neg											Neg						
198	failure	Neg	Neg											Neg						
199	high-risk	4+	Pos	S										Pos	S	S				
200	high-risk	S	Neg											Neg						
201	high-burden	Neg	Neg											Neg						
202	failure	1+	Pos	S										Neg						
203	relapse	Neg	Neg											Neg						
204	failure	2+	Pos	S										Pos	S	S				
205	relapse	Neg	Neg											Neg						
206	relapse	Neg	Neg											Neg						
207	high-burden	Neg	Neg											Pos	S	S				
208	high-burden	Neg	Neg											Neg						
209	default	Neg	Neg											Neg						
210	default	Neg	Neg											Neg						
211	relapse	S	Neg											Neg						
212	failure	2+	Pos	S										Pos	S	S				
213	relapse	1+	Pos	S										Pos	S	S				
214	relapse	Neg	Neg											NTM						
215	relapse	1+	Neg											NTM						
216	relapse	Neg	Neg											Neg						
217	high-burden	Neg	Neg											Neg						
218	failure	Neg	Pos	S										Pos	S	S				
219	high-risk	4+	Pos	S										Pos	S	S				
220	failure	1+	Pos	S										Neg						
221	failure	3+	Pos	S										Missing						

Pt#	Group	AFS	Xpert			DR*plus* (WT absent, MUT binding)		DR*sl* (WT absent, MUT binding)		Sequencing (Discordant)				Convetional results						
			MTBC	RIF	Probe Failure	RIF	INH	FLQ	SLID	RIF	INH	FLQ	SLID	Cul.	INH	RIF	FLQ	KAN	AMK	CAP
222	relapse	Neg	Neg											Neg						
223	high-burden	Neg	Neg											Neg						
224	failure	1+	Pos	S										Neg						
225	high-burden	Neg	Neg											Neg						
226	high-burden	Neg	Neg											Neg						
227	failure	S	Pos	S										Pos	S	S				
228	relapse	Neg	Neg											Neg						
229	high-risk	Neg	Neg											Neg						
230	high-burden	Neg	Neg											Neg						
231	relapse	Neg	Neg											Neg						
232	relapse	Neg	Pos	S										Pos	R	S				
233	high-risk	4+	Pos	S										Pos	S	S				
234	high-risk	Neg	Neg											Neg						
235	high-risk	Neg	Neg											Missing						
236	high-burden	2+	Pos	S										Pos	R	S				
237	failure	Neg	Neg											Neg						
238	high-risk	Neg	Neg											Pos	S	S				
239	failure	1+	Neg											Neg						
240	failure	1+	Pos	S										Pos	S	S				
241	failure	Neg	Neg											Neg						
242	high-burden	Neg	Neg											Pos	S	S				
243	high-burden	Neg	Neg											Neg						
244	relapse	Neg	Pos	S										Pos	S	S				
245	relapse	Neg	Pos	S										Pos	R	S				
246	high-burden	Neg	Neg											Neg						
247	failure	1+	Pos	S										Neg						
248	failure	1+	Neg											Neg						
249	failure	1+	Pos	S										NTM						

Pt#	Group	AFS	Xpert			DR*plus* (WT absent, MUT binding)		DR*sl* (WT absent, MUT binding)		Sequencing (Discordant)				Convetional results						
			MTBC	RIF	Probe Failure	RIF	INH	FLQ	SLID	RIF	INH	FLQ	SLID	Cul.	INH	RIF	FLQ	KAN	AMK	CAP
250	high-risk	Neg	Neg											Neg						
251	failure	S	Pos	S										Neg						
252	relapse	Neg	Neg											Neg						
253	failure	Neg	Pos	S										Neg						
254	failure	Neg	Neg											Neg						
255	high-burden	Neg	Neg											Neg						
256	relapse	Neg	Neg											NTM						
257	failure	Neg	Neg											Neg						
258	relapse	1+	Pos	R	E	WT8,MUT3	*katG* WT,MUT1<br>*inhA* WT1,MUT1	*gyrA* WT2,MUT1				*gyrA* A90V		Pos	R	R	S	S	S	S
259	failure	Neg	Neg											Neg						
260	high-burden	Neg	Neg											Neg						
261	high-risk	Neg	Pos	S										Pos	S	S				
262	failure	Neg	Pos	S										Pos	R	S				
263	high-risk	Neg	Neg											Neg						
264	high-risk	Neg	Neg											Neg						
265	high-risk	Neg	Neg											Neg						
266	high-burden	Neg	Neg											Neg						
267	high-risk	Neg	Neg											Neg						
268	high-risk	Neg	Neg											Neg						
269	relapse	S	Pos	S										Pos	S	S				
270	failure	Neg	Pos	S										Neg						
271	relapse	Neg	Neg											Neg						
272	relapse	Neg	Neg											Neg						
273	relapse	Neg	Neg											Neg						
274	failure	Neg	Pos	S										Pos	S	S				
275	default	Neg	Neg											Neg						
276	failure	Neg	Pos	S										Neg						
277	high-burden	Neg	Neg											Pos	S	S				

Pt#	Group	AFS	Xpert			DR*plus* (WT absent, MUT binding)		DR*sl* (WT absent, MUT binding)		Sequencing (Discordant)				Convetional results						
			MTBC	RIF	Probe Failure	RIF	INH	FLQ	SLID	RIF	INH	FLQ	SLID	Cul.	INH	RIF	FLQ	KAN	AMK	CAP
278	high-risk	Neg	Neg											Neg						
279	relapse	S	Neg											Neg						
280	relapse	Neg	Neg											Neg						
281	high-risk	4+	Pos	S										Pos	S	S				
282	relapse	Neg	Neg											Neg						
283	high-burden	Neg	Neg											Neg						
284	failure	2+	Neg											Neg						
285	high-burden	Neg	Neg											Neg						
286	default	Neg	Neg											Neg						
287	high-burden	Neg	Neg											Neg						
288	high-burden	Neg	Neg											Neg						
289	relapse	2+	Neg											NTM						
290	relapse	Neg	Neg											Neg						
291	high-burden	Neg	Neg											Neg						
292	relapse	1+	Pos	S										Pos	S	S				
293	failure	S	Pos	S										Neg						
294	high-burden	Neg	Neg											Neg						
295	high-burden	Neg	Neg											Neg						
296	high-burden	Neg	Neg											Neg						
297	relapse	Neg	Neg											Neg						
298	failure	S	Pos	S										Pos	S	S				
299	high-burden	2+	Pos	S										Pos	S	S				
300	relapse	Neg	Pos	R	E	WT8,MUT3	*katG* WT,MUT1							Missing						
301	high-burden	Neg	Pos	S										Pos	S	S				
302	relapse	Neg	Neg											Neg						
303	relapse	Neg	Neg											Neg						
304	relapse	Neg	Neg											Neg						
305	high-risk	1+	Pos	S										Pos	S	S				

Pt#	Group	AFS	Xpert			DR*plus* (WT absent, MUT binding)		DR*sl* (WT absent, MUT binding)		Sequencing (Discordant)				Convetional results						
			MTBC	RIF	Probe Failure	RIF	INH	FLQ	SLID	RIF	INH	FLQ	SLID	Cul.	INH	RIF	FLQ	KAN	AMK	CAP
306	high-risk	S	Neg											Neg						
307	high-burden	Neg	Neg											Neg						
308	relapse	Neg	Neg											Neg						
309	failure	1+	Neg											NTM						
310	relapse	Neg	Neg											NTM						
311	high-burden	Neg	Neg											Neg						
312	high-burden	Neg	Neg											Neg						
313	high-burden	Neg	Neg											Neg						
314	high-risk	Neg	Neg											Neg						
315	high-risk	Neg	Neg											Neg						
316	relapse	Neg	Neg											Neg						
317	high-risk	Neg	Neg											NTM						
318	high-burden	Neg	Neg											Neg						
319	high-burden	Neg	Neg											Neg						
320	relapse	Neg	Neg											Neg						
321	relapse	Neg	Neg											Neg						
322	failure	S	Pos	S										Neg						
323	high-burden	4+	Pos	S										Pos	R	S				
324	failure	Neg	Neg											Neg						
325	relapse	Neg	Neg											Neg						
326	failure	Neg	Pos	S										Neg						
327	failure	1+	Pos	S										Neg						
328	failure	1+	Pos	S										Pos	S	S				
329	relapse	Neg	Neg											Pos	S	S				
330	relapse	1+	Pos	R	E	WT8,MUT3	*inhA* WT1,MUT1							Pos	R	R	S	S	S	S
331	relapse	Neg	Neg											Neg						
332	high-burden	Neg	Neg											Neg						
333	failure	Neg	Neg											Neg						

Pt#	Group	AFS	Xpert			DR*plus* (WT absent, MUT binding)		DR*sl* (WT absent, MUT binding)		Sequencing (Discordant)				Convetional results						
			MTBC	RIF	Probe Failure	RIF	INH	FLQ	SLID	RIF	INH	FLQ	SLID	Cul.	INH	RIF	FLQ	KAN	AMK	CAP
334	default	Neg	Neg											Neg						
335	relapse	Neg	Neg											Neg						
336	high-burden	Neg	Pos	S										Neg						
337	relapse	S	Pos	S										Neg						
338	high-risk	Neg	Neg											Neg						
339	relapse	Neg	Neg											Neg						
340	high-burden	Neg	Neg											Neg						
341	failure	Neg	Neg											Neg						
342	high-burden	Neg	Neg											Pos	S	S				
343	high-burden	1+	Pos	S										Pos	R	S				
344	relapse	2+	Pos	S										Pos	S	S				
345	failure	S	Pos	S										Pos	S	S				
346	relapse	Neg	Neg											Neg						
347	high-burden	Neg	Neg											Neg						
348	relapse	Neg	Neg											Neg						
349	high-risk	Neg	Pos	S										Pos	S	S				
350	high-risk	S	Pos	S										Pos	S	S				
351	failure	2+	Neg											Neg						
352	relapse	Neg	Neg											Neg						
353	relapse	Neg	Pos	S										Pos	S	S				
354	high-burden	Neg	Neg											Neg						
355	relapse	Neg	Neg											Neg						
356	high-burden	Neg	Neg											Neg						
357	relapse	Neg	Neg											Neg						
358	relapse	2+	Neg											Neg						
359	high-risk	4+	Pos	S										Pos	S	S				
360	high-burden	Neg	Neg											Neg						
361	relapse	Neg	Neg											Neg						

Pt#	Group	AFS	Xpert			DR*plus* (WT absent, MUT binding)		DR*sl* (WT absent, MUT binding)		Sequencing (Discordant)				Convetional results						
			MTBC	RIF	Probe Failure	RIF	INH	FLQ	SLID	RIF	INH	FLQ	SLID	Cul.	INH	RIF	FLQ	KAN	AMK	CAP
362	high-burden	Neg	Neg											Neg						
363	high-burden	Neg	Neg											Neg						
364	high-burden	Neg	Neg											Neg						
365	failure	Neg	Pos	S										Neg						
366	relapse	Neg	Neg											Neg						
367	high-burden	Neg	Neg											Neg						
368	failure	S	Pos	S										Neg						
369	relapse	2+	Pos	S										Pos	S	S				
370	failure	Neg	Neg											Neg						
371	high-burden	Neg	Neg											Neg						
372	relapse	Neg	Neg											Neg						
373	failure	Neg	Neg											Neg						
374	failure	Neg	Neg											Neg						
375	failure	1+	Pos	S										Neg						
376	relapse	Neg	Neg											NTM						
377	failure	Neg	Neg											Neg						
378	high-risk	Neg	Neg											Pos	S	S				
379	relapse	1+	Neg											Neg						
380	high-risk	2+	Pos	S										Pos	S	S				
381	relapse	Neg	Neg											Neg						
382	high-risk	4+	Pos	S										Pos	S	S				
383	high-burden	1+	Neg											Neg						
384	failure	1+	Neg											Neg						
385	relapse	Neg	Neg											Neg						
386	relapse	Neg	Neg											Neg						
387	high-burden	Neg	Pos	S										Pos	S	S				
388	relapse	Neg	Neg											Neg						
389	failure	1+	Pos	S										Pos	S	S				

Pt#	Group	AFS	Xpert			DR*plus* (WT absent, MUT binding)		DR*sl* (WT absent, MUT binding)		Sequencing (Discordant)				Convetional results						
			MTBC	RIF	Probe Failure	RIF	INH	FLQ	SLID	RIF	INH	FLQ	SLID	Cul.	INH	RIF	FLQ	KAN	AMK	CAP
390	failure	Neg	Neg											Neg						
391	default	Neg	Neg											Neg						
392	high-burden	Neg	Neg											Neg						
393	high-burden	S	Pos	S										Pos	S	S				
394	relapse	Neg	Pos	R	A	WT2	S							Missing						
395	high-burden	Neg	Neg											Neg						
396	failure	1+	Pos	S										Neg						
397	high-burden	Neg	Neg											Neg						
398	high-risk	Neg	Pos	S										Pos	S	S				
399	high-risk	4+	Pos	S										Pos	S	S				
400	relapse	Neg	Neg											Neg						
401	relapse	1+	Pos	S										Pos	S	S				
402	high-burden	Neg	Neg											Pos	S	S				
403	high-burden	Neg	Neg											Neg						
404	high-risk	Neg	Neg											Neg						
405	relapse	2+	Neg											Neg						
406	high-risk	Neg	Neg											Neg						
407	high-burden	Neg	Neg											Neg						
408	failure	1+	Pos	S										Pos	S	S				
409	failure	1+	Neg											NTM						
410	failure	1+	Pos	S										Neg						
411	failure	S	Neg											Neg						
412	failure	S	Neg											Neg						
413	relapse	Neg	Neg											NTM						
414	failure	2+	Pos	S										Neg						
415	failure	1+	Pos	S										Pos	S	S				
416	relapse	4+	Pos	S										Pos	S	S				
417	relapse	Neg	Neg											Neg						

Pt#	Group	AFS	Xpert			DR*plus* (WT absent, MUT binding)		DR*sl* (WT absent, MUT binding)		Sequencing (Discordant)				Convetional results						
			MTBC	RIF	Probe Failure	RIF	INH	FLQ	SLID	RIF	INH	FLQ	SLID	Cul.	INH	RIF	FLQ	KAN	AMK	CAP
418	high-burden	Neg	Neg											Neg						
419	default	Neg	Neg											Neg						
420	high-burden	Neg	Neg											Neg						
421	unknown	Neg	Neg											Neg						
422	failure	1+	Pos	S										Pos	S	S				
423	relapse	S	Pos	S										Pos	R	S				
424	high-burden	Neg	Neg											Neg						
425	failure	Neg	Neg											NTM						
426	default	Neg	Neg											Neg						
427	failure	Neg	Pos	S										Neg						
428	failure	1+	Pos	S										Neg						
429	relapse	Neg	Neg											Neg						
430	failure	1+	Pos	S										Neg						
431	high-burden	Neg	Neg											Neg						
432	relapse	Neg	Neg											Neg						
433	high-burden	Neg	Neg											Neg						
434	failure	Neg	Neg											Neg						
435	failure	Neg	Neg											Pos	S	S				
436	high-burden	Neg	Neg											Neg						
437	relapse	Neg	Neg											Neg						
438	failure	S	Pos	S										Pos	S	S				
439	relapse	Neg	Neg											NTM						
440	relapse	Neg	Neg											Pos	S	S				
441	relapse	Neg	Neg											Neg						
442	failure	1+	Pos	S										Neg						
443	high-burden	2+	Pos	S										Pos	R	S				
444	failure	1+	Neg											Neg						
445	failure	Neg	Neg											Neg						

Pt#	Group	AFS	Xpert			DR*plus* (WT absent, MUT binding)		DR*sl* (WT absent, MUT binding)		Sequencing (Discordant)				Convetional results						
			MTBC	RIF	Probe Failure	RIF	INH	FLQ	SLID	RIF	INH	FLQ	SLID	Cul.	INH	RIF	FLQ	KAN	AMK	CAP
446	failure	Neg	Neg											Neg						
447	failure	Neg	Neg											Neg						
448	failure	S	Neg											Neg						
449	failure	1+	Neg											Neg						
450	high-risk	Neg	Neg											Neg						
451	high-burden	Neg	Neg											Neg						
452	relapse	4+	Pos	S										Pos	S	S				
453	relapse	Neg	Neg											Neg						
454	relapse	Neg	Neg											Neg						
455	failure	Neg	Pos	S										Pos	S	S				
456	high-risk	Neg	Neg											Neg						
457	failure	2+	Pos	S										Pos	S	S				
458	high-burden	Neg	Neg											Neg						
459	failure	1+	Pos	S										Pos	S	S				
460	high-burden	Neg	Neg											NTM						
461	relapse	Neg	Neg											Neg						
462	failure	Neg	Neg											NTM						
463	high-burden	Neg	Neg											Neg						
464	high-burden	Neg	Neg											Neg						
465	relapse	Neg	Neg											Neg						
466	high-burden	Neg	Neg											Neg						
467	high-risk	Neg	Neg											Neg						
468	relapse	Neg	Neg											Neg						
469	high-risk	2+	Pos	S										Pos	S	S				
470	relapse	Neg	Neg											Neg						
471	relapse	Neg	Neg											Neg						
472	high-burden	Neg	Neg											Neg						
473	failure	1+	Neg											Neg						

Pt#	Group	AFS	Xpert			DR*plus* (WT absent, MUT binding)		DR*sl* (WT absent, MUT binding)		Sequencing (Discordant)				Convetional results						
			MTBC	RIF	Probe Failure	RIF	INH	FLQ	SLID	RIF	INH	FLQ	SLID	Cul.	INH	RIF	FLQ	KAN	AMK	CAP
474	high-risk	Neg	Neg											Neg						
475	high-risk	Neg	Pos	S										Pos	S	S				
476	high-risk	Neg	Neg											Neg						
477	high-risk	Neg	Neg											Neg						
478	relapse	Neg	Neg											Neg						
479	failure	2+	Pos	S										Pos	S	S				
480	relapse	Neg	Pos	S										Pos	S	S				
481	high-burden	Neg	Neg											Neg						
482	high-risk	1+	Pos	S										Pos	S	S				
483	high-risk	Neg	Neg											Neg						
484	high-risk	Neg	Neg											Neg						
485	high-burden	Neg	Neg											Neg						
486	failure	Neg	Neg											Neg						
487	relapse	Neg	Neg											Neg						
488	high-burden	1+	Neg											Neg						
489	high-burden	Neg	Neg											Neg						
490	high-burden	4+	Pos	S										Pos	S	S				
491	failure	1+	Pos	S										Neg						
492	relapse	Neg	Neg											Neg						
493	relapse	Neg	Neg											Neg						
494	high-risk	Neg	Neg											Neg						
495	failure	1+	Pos	S										Neg						
496	high-burden	Neg	Neg											Neg						
497	failure	S	Pos	S										Pos	S	S				
498	relapse	Neg	Neg											Neg						
499	failure	Neg	Neg											Neg						
500	failure	2+	Neg											Neg						
501	relapse	Neg	Neg											Pos	S	S				

Pt#	Group	AFS	Xpert			DR*plus* (WT absent, MUT binding)		DR*sl* (WT absent, MUT binding)		Sequencing (Discordant)				Convetional results						
			MTBC	RIF	Probe Failure	RIF	INH	FLQ	SLID	RIF	INH	FLQ	SLID	Cul.	INH	RIF	FLQ	KAN	AMK	CAP
502	high-burden	Neg	Neg											Neg						
503	relapse	Neg	Neg											Neg						
504	relapse	Neg	Neg											Neg						
505	relapse	Neg	Neg											Neg						
506	failure	3+	Pos	R	E	WT8,MUT3	S							Pos	-	-	-	-	-	-
507	failure	2+	Neg											Missing						
508	default	Neg	Neg											Neg						
509	relapse	2+	Pos	S										Pos	S	S				
510	high-burden	Neg	Neg											Neg						
511	high-burden	S	Neg											Neg						
512	high-burden	Neg	Neg											Neg						
513	high-burden	Neg	Neg											Neg						
514	relapse	2+	Pos	S										Neg						
515	high-burden	Neg	Neg											Neg						
516	relapse	4+	Neg											Neg						
517	high-risk	Neg	Neg											Neg						
518	relapse	Neg	Neg											Neg						
519	relapse	Neg	Neg											Neg						
520	relapse	4+	Pos	S										Pos	S	S				
521	high-burden	Neg	Neg											Neg						
522	high-burden	Neg	Pos	S										Pos	S	S				
523	high-burden	Neg	Pos	S										Pos	S	S				
524	relapse	Neg	Neg											Neg						
525	high-risk	1+	Neg											NTM						
526	relapse	Neg	Neg											Neg						
527	relapse	Neg	Neg											Neg						
528	high-burden	2+	Pos	S										Pos	S	S				
529	relapse	Neg	Pos	R	D	WT7,MUT2A	S							Pos	S	R	S	S	S	S

Pt#	Group	AFS	Xpert			DR*plus* (WT absent, MUT binding)		DR*sl* (WT absent, MUT binding)		Sequencing (Discordant)				Convetional results						
			MTBC	RIF	Probe Failure	RIF	INH	FLQ	SLID	RIF	INH	FLQ	SLID	Cul.	INH	RIF	FLQ	KAN	AMK	CAP
530	relapse	Neg	Neg											NTM						
531	default	Neg	Pos	S										Pos	R	S				
532	relapse	Neg	Neg											NTM						
533	high-risk	Neg	Neg											Neg						
534	failure	3+	Neg											NTM						
535	high-burden	Neg	Neg											Neg						
536	failure	1+	Pos	S										Neg						
537	relapse	Neg	Neg											Neg						
538	high-burden	Neg	Neg											Neg						
539	high-burden	Neg	Neg											Neg						
540	relapse	Neg	Pos	S										Pos	S	S				
541	high-risk	Neg	Neg											Pos	S	S				
542	high-risk	Neg	Neg											Pos	S	S				
543	high-risk	4+	Pos	R	A	WT1	S			G507G				Pos	S	S	S	S	-	S
544	high-burden	Neg	Neg											Neg						
545	high-risk	4+	Neg											Pos	S	S				
546	relapse	Neg	Neg											Neg						
547	high-risk	1+	Pos	S										Pos	R	S				
548	relapse	Neg	Neg											Neg						
549	failure	Neg	Neg											Neg						
550	failure	2+	Pos	S										Pos	S	S				
551	relapse	Neg	Pos	S										Neg						
552	high-risk	Neg	Neg											Neg						
553	high-burden	Neg	Neg											Neg						
554	relapse	Neg	Neg											Neg						
555	high-burden	Neg	Neg											Neg						
556	high-burden	2+	Pos	S										Pos	S	S				

Pt#	Group	AFS	Xpert			DR*plus* (WT absent, MUT binding)		DR*sl* (WT absent, MUT binding)		Sequencing (Discordant)				Convetional results						
			MTBC	RIF	Probe Failure	RIF	INH	FLQ	SLID	RIF	INH	FLQ	SLID	Cul.	INH	RIF	FLQ	KAN	AMK	CAP
557	failure	Neg	Neg											Neg						
558	high-burden	Neg	Neg											Neg						
559	relapse	Neg	Neg											Neg						
560	relapse	S	Pos	S										Pos	S	S				
561	high-burden	Neg	Neg											Pos	R	S				
562	relapse	1+	Neg											Neg						
563	relapse	3+	Neg											Neg						
564	high-burden	Neg	Neg											Neg						
565	failure	Neg	Neg											Neg						
566	relapse	1+	Pos	S										Pos	S	S				
567	high-risk	Neg	Neg											Neg						
568	relapse	Neg	Neg											Neg						
569	high-risk	Neg	Neg											Neg						
570	relapse	Neg	Neg											Neg						
571	high-burden	Neg	Neg											Neg						
572	relapse	Neg	Pos	I										Pos	S	S				
573	relapse	Neg	Neg											NTM						
574	high-burden	Neg	Neg											NTM						
575	failure	Neg	Neg											Neg						
576	high-risk	2+	Pos	S										Pos	R	S				
577	failure	S	Neg											Neg						
578	relapse	Neg	Neg											Neg						
579	high-burden	S	Neg											Neg						
580	relapse	4+	Pos	S										Pos	S	S	S	S	-	S
581	high-burden	Neg	Neg											Neg						
582	relapse	S	Neg											Neg						
583	high-risk	Neg	Pos	S										Pos	S	S				
584	close contacts	Neg	Neg											Neg						

Pt#	Group	AFS	Xpert			DR*plus* (WT absent, MUT binding)		DR*sl* (WT absent, MUT binding)		Sequencing (Discordant)				Convetional results						
			MTBC	RIF	Probe Failure	RIF	INH	FLQ	SLID	RIF	INH	FLQ	SLID	Cul.	INH	RIF	FLQ	KAN	AMK	CAP
585	relapse	Neg	Neg											Neg						
586	relapse	Neg	Pos	S										Pos	S	S				
587	high-burden	Neg	Neg											Neg						
588	relapse	4+	Pos	S										Pos	R	S				
589	high-risk	Neg	Neg											Neg						
590	high-risk	4+	Pos	S										Pos	S	S				
591	relapse	Neg	Neg											Neg						
592	high-burden	S	Neg											Pos	S	S				
593	relapse	Neg	Neg											Neg						
594	high-burden	3+	Neg											Neg						
595	failure	4+	Pos	S										Neg						
596	high-burden	Neg	Neg											Neg						
597	relapse	S	Neg											Neg						
598	relapse	Neg	Neg											Neg						
599	high-burden	Neg	Neg											Neg						
600	relapse	Neg	Neg											Neg						
601	high-burden	Neg	Neg											Neg						
602	high-burden	Neg	Neg											Neg						
603	high-burden	Neg	Neg											Neg						
604	relapse	Neg	Neg											Neg						
605	failure	1+	Neg											Neg						
606	failure	2+	Pos	S										Neg						
607	close contacts	Neg	Neg											Neg						
608	high-risk	1+	Pos	S										Pos	S	S				
609	high-risk	Neg	Neg											Neg						
610	failure	Neg	Neg											Neg						
611	failure	Neg	Neg											Neg						
612	failure	1+	Pos	S										Pos	S	S				

Pt#	Group	AFS	Xpert			DR*plus* (WT absent, MUT binding)		DR*sl* (WT absent, MUT binding)		Sequencing (Discordant)				Convetional results						
			MTBC	RIF	Probe Failure	RIF	INH	FLQ	SLID	RIF	INH	FLQ	SLID	Cul.	INH	RIF	FLQ	KAN	AMK	CAP
613	relapse	Neg	Neg											Neg						
614	high-risk	1+	Pos	S										Neg						
615	failure	Neg	Neg											Pos	S	S				
616	failure	S	Neg											Neg						
617	relapse	Neg	Neg											Neg						
618	relapse	Neg	Neg											NTM						
619	failure	Neg	Pos	S										Pos	S	S				
620	high-burden	Neg	Neg											Neg						
621	relapse	Neg	Neg											Neg						
622	high-burden	3+	Pos	S										Pos	S	S				
623	high-burden	S	Neg											Pos	S	S				
624	close contacts	3+	Pos	S										Pos	S	S				
625	failure	Neg	Neg											Neg						
626	relapse	Neg	Neg											Neg						
627	relapse	4+	Pos	S										Pos	R	S	S	S	-	S
628	high-burden	Neg	Neg											Neg						
629	relapse	Neg	Neg											NTM						
630	high-burden	Neg	Neg											Pos	S	S				
631	high-burden	Neg	Neg											Neg						
632	high-burden	Neg	Pos	S										Pos	R	S				
633	relapse	Neg	Neg											Neg						
634	high-burden	Neg	Neg											Neg						
635	failure	2+	Pos	S										Pos	S	S				
636	relapse	Neg	Neg											Neg						
637	relapse	Neg	Neg											Neg						
638	relapse	Neg	Pos	S										Neg						
639	high-burden	Neg	Neg											Neg						
640	relapse	Neg	Neg											Neg						

Pt#	Group	AFS	Xpert			DR*plus* (WT absent, MUT binding)		DR*sl* (WT absent, MUT binding)		Sequencing (Discordant)				Convetional results						
			MTBC	RIF	Probe Failure	RIF	INH	FLQ	SLID	RIF	INH	FLQ	SLID	Cul.	INH	RIF	FLQ	KAN	AMK	CAP
641	relapse	Neg	Neg											Neg						
642	high-burden	Neg	Neg											Pos	S	S				
643	high-burden	Neg	Neg											Neg						
644	high-burden	Neg	Pos	S										Pos	S	S				
645	relapse	Neg	Neg											Neg						
646	relapse	Neg	Neg											NTM						
647	relapse	Neg	Neg											Neg						
648	high-risk	Neg	Neg											Neg						
649	relapse	Neg	Neg											NTM						
650	high-burden	Neg	Neg											Neg						
651	relapse	Neg	Neg											Neg						
652	failure	Neg	Pos	S										Pos	S	S				
653	high-burden	Neg	Neg											Neg						
654	failure	Neg	Neg											Neg						
655	relapse	S	Neg											Neg						
656	high-burden	Neg	Neg											Neg						
657	relapse	Neg	Neg											Neg						
658	high-burden	Neg	Neg											Neg						
659	high-burden	Neg	Neg											Neg						
660	failure	Neg	Neg											Neg						
661	high-burden	Neg	Neg											Neg						
662	high-burden	Neg	Neg											Neg						
663	relapse	2+	Neg											NTM						
664	failure	1+	Pos	S										Neg						
665	high-risk	Neg	Neg											Neg						
666	high-burden	3+	Pos	S										Pos	S	S				
667	high-burden	Neg	Neg											Pos	S	S				
668	relapse	1+	Pos	S										Pos	S	S				

Pt#	Group	AFS	Xpert			DR*plus* (WT absent, MUT binding)		DR*sl* (WT absent, MUT binding)		Sequencing (Discordant)				Convetional results						
			MTBC	RIF	Probe Failure	RIF	INH	FLQ	SLID	RIF	INH	FLQ	SLID	Cul.	INH	RIF	FLQ	KAN	AMK	CAP
669	high-burden	Neg	Neg											Neg						
670	failure	2+	Pos	S										Pos	S	S				
671	high-burden	Neg	Neg											Neg						
672	high-burden	Neg	Neg											Neg						
673	relapse	Neg	Neg											Neg						
674	relapse	4+	Pos	S										Pos	S	S				
675	relapse	Neg	Neg											Missing						
676	high-burden	Neg	Neg											Neg						
677	high-burden	Neg	Neg											Neg						
678	high-burden	Neg	Neg											Neg						
679	high-burden	Neg	Pos	S										Pos	S	S				
680	relapse	Neg	Neg											Neg						
681	high-burden	Neg	Neg											Neg						
682	high-burden	Neg	Neg											Neg						
683	relapse	Neg	Neg											Neg						
684	high-burden	Neg	Neg											Neg						
685	relapse	Neg	Neg											Neg						
686	high-burden	Neg	Neg											Pos	S	S				
687	relapse	1+	Pos	S										Neg						
688	default	Neg	Neg											Neg						
689	high-burden	Neg	Neg											Missing						
690	high-burden	Neg	Neg											Neg						
691	high-burden	Neg	Neg											Neg						
692	failure	Neg	Pos	S										Pos	S	S				
693	failure	1+	Pos	S										Pos	S	S	S	S	-	S
694	failure	4+	Neg											Neg						
695	relapse	Neg	Neg											Neg						
696	high-risk	4+	Pos	S										Pos	R	S				

Pt#	Group	AFS	Xpert			DR*plus* (WT absent, MUT binding)		DR*sl* (WT absent, MUT binding)		Sequencing (Discordant)				Convetional results						
			MTBC	RIF	Probe Failure	RIF	INH	FLQ	SLID	RIF	INH	FLQ	SLID	Cul.	INH	RIF	FLQ	KAN	AMK	CAP
697	high-risk	1+	Pos	S										Pos	S	S				
698	relapse	Neg	Neg											Neg						
699	relapse	1+	Pos	S										Pos	S	S				
700	relapse	S	Neg											Neg						
701	relapse	Neg	Neg											NTM						
702	high-burden	Neg	Neg											Pos	S	S				
703	high-burden	Neg	Neg											Neg						
704	failure	1+	Neg											Neg						
705	relapse	Neg	Neg											Neg						
706	failure	Neg	Neg											Neg						
707	relapse	Neg	Neg											Neg						
708	relapse	Neg	Neg											Neg						
709	failure	Neg	Pos	S										Neg						
710	high-burden	Neg	Pos	S										Neg						
711	relapse	1+	Pos	S										Pos	S	S				
712	high-burden	Neg	Neg											Neg						
713	high-burden	Neg	Pos	S										Pos	S	S				
714	high-burden	1+	Pos	S										Pos	S	S				
715	high-burden	Neg	Neg											Neg						
716	relapse	2+	Neg											Neg						
717	high-burden	Neg	Neg											Neg						
718	high-burden	Neg	Neg											Neg						
719	relapse	1+	Pos	S										Pos	S	S				
720	failure	1+	Pos	S										Pos	S	S				
721	relapse	Neg	Neg											Neg						
722	high-burden	Neg	Neg											Neg						
723	high-risk	1+	Pos	S										Pos	S	S				
724	failure	Neg	Neg											Neg						

Pt#	Group	AFS	Xpert			DR*plus* (WT absent, MUT binding)		DR*sl* (WT absent, MUT binding)		Sequencing (Discordant)				Convetional results						
			MTBC	RIF	Probe Failure	RIF	INH	FLQ	SLID	RIF	INH	FLQ	SLID	Cul.	INH	RIF	FLQ	KAN	AMK	CAP
725	failure	1+	Pos	S										Pos	S	S				
726	high-risk	Neg	Neg											Neg						
727	high-burden	Neg	Neg											Neg						
728	high-risk	Neg	Neg											Neg						
729	high-risk	Neg	Pos	S										Pos	R	S				
730	relapse	1+	Pos	S										Neg						
731	relapse	2+	Pos	S										Pos	S	S				
732	relapse	Neg	Neg											Neg						
733	relapse	Neg	Neg											NTM						
734	high-burden	Neg	Pos	S										Neg						
735	high-burden	Neg	Neg											Neg						
736	high-burden	Neg	Neg											Neg						
737	relapse	Neg	Neg											Neg						
738	relapse	Neg	Neg											Neg						
739	relapse	1+	Neg											Pos	R	S				
740	failure	S	Neg											Neg						
741	high-burden	4+	Pos	S										Pos	S	S				
742	relapse	Neg	Neg											Neg						
743	relapse	Neg	Neg											Neg						
744	relapse	1+	Pos	S										Pos	S	S				
745	failure	S	Pos	S										Pos	S	S				
746	high-burden	S	Neg											Neg						
747	failure	Neg	Neg											Neg						
748	failure	1+	Pos	S										Neg						
749	failure	1+	Neg											Neg						
750	failure	1+	Neg											Neg						
751	high-risk	1+	Neg											Neg						
752	relapse	Neg	Neg											Neg						

Pt#	Group	AFS	Xpert			DR*plus* (WT absent, MUT binding)		DR*sl* (WT absent, MUT binding)		Sequencing (Discordant)				Convetional results						
			MTBC	RIF	Probe Failure	RIF	INH	FLQ	SLID	RIF	INH	FLQ	SLID	Cul.	INH	RIF	FLQ	KAN	AMK	CAP
753	high-burden	Neg	Neg											Neg						
754	high-burden	1+	Neg											Neg						
755	failure	1+	Neg											Neg						
756	high-burden	Neg	Neg											Neg						
757	high-burden	Neg	Neg											Neg						
758	high-burden	Neg	Neg											Pos	S	S				
759	high-burden	Neg	Neg											Pos	S	S				
760	relapse	4+	Pos	S										Pos	S	S				
761	failure	Neg	Neg											Neg						
762	failure	Neg	Neg											Neg						
763	failure	Neg	Neg											Neg						
764	high-burden	Neg	Neg											Pos	S	S				
765	relapse	Neg	Neg											Neg						
766	relapse	Neg	Neg											Neg						
767	relapse	Neg	Neg											Neg						
768	high-risk	Neg	Neg											Neg						
769	relapse	Neg	Neg											Neg						
770	unknown	Neg	Pos	S										Pos	S	S				
771	unknown	Neg	Pos	S										Pos	S	S				
772	unknown	Neg	Pos	S										Pos	S	S				
773	unknown	Neg	Neg											Pos	S	S				
774	unknown	Neg	Neg											Neg						
775	relapse	S	Neg											Neg						
776	high-burden	Neg	Neg											Neg						
777	high-burden	Neg	Neg											Pos	S	S				
778	relapse	Neg	Pos	S										Pos	S	S				
779	relapse	3+	Neg											Neg						
780	high-burden	Neg	Neg											Neg						

Pt#	Group	AFS	Xpert			DR*plus* (WT absent, MUT binding)		DR*sl* (WT absent, MUT binding)		Sequencing (Discordant)				Convetional results						
			MTBC	RIF	Probe Failure	RIF	INH	FLQ	SLID	RIF	INH	FLQ	SLID	Cul.	INH	RIF	FLQ	KAN	AMK	CAP
781	high-burden	Neg	Neg											Neg						
782	high-burden	Neg	Neg											Neg						
783	failure	3+	Pos	S										Neg						
784	high-risk	1+	Pos	S										Pos	S	S				
785	high-risk	Neg	Neg											Neg						
786	failure	2+	Pos	S										Pos	S	S				
787	failure	1+	Neg											Neg						
788	failure	Neg	I											Neg						
789	failure	1+	Pos	S										Pos	S	S				
790	high-burden	Neg	Neg											Neg						
791	failure	Neg	Pos	S										Neg						
792	high-burden	Neg	Neg											Neg						
793	high-burden	Neg	Neg											Pos	S	S				
794	high-burden	1+	Neg											Neg						
795	failure	Neg	Pos	S										Neg						
796	high-burden	Neg	Neg											Neg						
797	high-burden	Neg	Neg											Neg						
798	relapse	Neg	Neg											Neg						
799	relapse	Neg	Neg											Neg						
800	failure	1+	Neg											Neg						
801	relapse	Neg	Neg											NTM						
802	relapse	Neg	Neg											Neg						
803	relapse	Neg	Neg											Neg						
804	relapse	Neg	Neg											Neg						
805	relapse	Neg	Pos	S										Pos	R	S				
806	failure	1+	Pos	S										Pos	S	S				
807	high-burden	S	Neg											Neg						
808	high-burden	Neg	Neg											Neg						

Pt#	Group	AFS	Xpert			DR*plus* (WT absent, MUT binding)		DR*sl* (WT absent, MUT binding)		Sequencing (Discordant)				Convetional results						
			MTBC	RIF	Probe Failure	RIF	INH	FLQ	SLID	RIF	INH	FLQ	SLID	Cul.	INH	RIF	FLQ	KAN	AMK	CAP
809	high-burden	Neg	Neg											Neg						
810	high-burden	Neg	Neg											Neg						
811	high-risk	Neg	Neg											Pos	S	S				
812	high-risk	1+	Pos	S										Pos	S	S				
813	high-risk	1+	Pos	R	D	WT7	S			H526N				Pos	S	S	S	S	-	S
814	high-risk	Neg	Neg											NTM						
815	relapse	Neg	Neg											Neg						
816	relapse	Neg	Neg											NTM						
817	close contacts	2+	Pos	S										Pos	S	S				
818	relapse	Neg	Neg											Neg						
819	relapse	Neg	Neg											Neg						
820	high-burden	Neg	Neg											Neg						
821	high-burden	S	Neg											Neg						
822	default	Neg	Neg											Neg						
823	failure	Neg	Pos	S										Pos	S	S				
824	relapse	Neg	Neg											Neg						
825	high-burden	Neg	Neg											Neg						
826	high-burden	Neg	Neg											Neg						
827	relapse	Neg	Neg											Neg						
828	failure	Neg	Neg											Neg						
829	relapse	Neg	Neg											Neg						
830	high-burden	Neg	Neg											Neg						
831	failure	3+	Pos	S										Neg						
832	failure	2+	Neg											Neg						
833	failure	Neg	Pos	S										Neg						
834	relapse	S	Neg											Neg						
835	high-burden	Neg	Neg											Neg						

Pt#	Group	AFS	Xpert			DR*plus* (WT absent, MUT binding)		DR*sl* (WT absent, MUT binding)		Sequencing (Discordant)				Convetional results						
			MTBC	RIF	Probe Failure	RIF	INH	FLQ	SLID	RIF	INH	FLQ	SLID	Cul.	INH	RIF	FLQ	KAN	AMK	CAP
836	relapse	Neg	Pos	S										Pos	S	S				
837	failure	1+	Neg											NTM						
838	failure	1+	Neg											Neg						
839	failure	1+	Pos	I										Neg						
840	failure	1+	Neg											Neg						
841	high-burden	Neg	Neg											Neg						
842	high-burden	Neg	Neg											Pos	S	S				
843	failure	S	Neg											Neg						
844	high-burden	Neg	Neg											Neg						
845	failure	1+	Pos	S										Neg						
846	high-burden	Neg	Neg											Neg						
847	failure	4+	Neg											NTM						
848	failure	Neg	Neg											Neg						
849	high-risk	Neg	Neg											Pos	S	S				
850	high-risk	Neg	Neg											Neg						
851	failure	Neg	Neg											Neg						
852	default	Neg	Neg											Neg						
853	failure	1+	Neg											Neg						
854	failure	1+	Pos	S										Neg						
855	high-risk	Neg	Neg											Pos	S	S				
856	high-burden	Neg	Neg											Neg						
857	high-burden	Neg	Neg											Neg						
858	high-burden	Neg	Neg											Neg						
859	relapse	Neg	Neg											Neg						
860	relapse	Neg	Neg											Neg						
861	failure	2+	Pos	S										Pos	S	S				
862	default	Neg	Neg											Neg						
863	relapse	S	Neg											Neg						

Pt#	Group	AFS	Xpert			DR*plus* (WT absent, MUT binding)		DR*sl* (WT absent, MUT binding)		Sequencing (Discordant)				Convetional results						
			MTBC	RIF	Probe Failure	RIF	INH	FLQ	SLID	RIF	INH	FLQ	SLID	Cul.	INH	RIF	FLQ	KAN	AMK	CAP
864	high-risk	Neg	Neg											Pos	S	S				
865	high-risk	4+	Pos	S										Pos	S	S				
866	high-burden	Neg	Pos	S										Pos	S	S				
867	relapse	Neg	Neg											Neg						
868	high-burden	2+	Pos	S										Pos	S	S				
869	relapse	Neg	Neg											Neg						
870	high-burden	Neg	Pos	S										Pos	S	S				
871	high-burden	Neg	Neg											Neg						
872	relapse	Neg	Neg											Neg						
873	high-burden	S	Neg											Pos	S	S				
874	failure	Neg	Pos	S										Pos	S	S				
875	high-risk	Neg	Neg											Neg						
876	relapse	Neg	Neg											Neg						
877	high-risk	Neg	Pos	S										Pos	S	S				
878	high-risk	4+	Pos	S										Pos	S	S				
879	failure	1+	Pos	S										Pos	S	S				
880	unknown	Neg	Neg											Neg						
881	relapse	1+	Neg											Neg						
882	high-risk	1+	Pos	S										Pos	S	S				
883	unknown	Neg	Neg											Neg						
884	unknown	Neg	Neg											Neg						
885	high-burden	Neg	Neg											Neg						
886	failure	2+	Pos	R	C	WT5,W 6	S			L521L				Pos	S	S	S	S	S	S
887	unknown	1+	Pos	S										Neg						
888	failure	1+	Pos	S										NTM						
889	failure	1+	Neg											NTM						
890	high-risk	2+	Pos	S										Pos	S	S				
891	high-risk	Neg	Neg											Neg						

Pt#	Group	AFS	Xpert			DR*plus* (WT absent, MUT binding)		DR*sl* (WT absent, MUT binding)		Sequencing (Discordant)				Convetional results						
			MTBC	RIF	Probe Failure	RIF	INH	FLQ	SLID	RIF	INH	FLQ	SLID	Cul.	INH	RIF	FLQ	KAN	AMK	CAP
892	relapse	1+	Neg											Neg						
893	failure	1+	Neg											Neg						
894	unknown	2+	Pos	S										Pos	R	S				
895	high-burden	Neg	Neg											Neg						
896	high-risk	S	Neg											Neg						
897	relapse	Neg	Neg											Neg						
898	unknown	Neg	Neg											Neg						
899	high-burden	Neg	Neg											Pos	S	S				
900	high-burden	Neg	Pos	R	E	WT8,MUT3	S							Pos	S	R	S	S	-	S
901	unknown	Neg	Neg											Neg						
902	unknown	2+	Pos	R	D	WT7,MUT2A	S							Pos	S	R	S	S	-	S
903	high-burden	Neg	Neg											Neg						
904	unknown	1+	Neg											Neg						
905	unknown	3+	Pos	S										Pos	S	S				
906	unknown	2+	Pos	S										Pos	S	S				
907	unknown	Neg	Neg											Neg						
908	high-burden	Neg	Neg											Pos	S	S				
909	failure	1+	Pos	S										Pos	S	S				
910	high-burden	Neg	Neg											Pos	S	S				
911	failure	Neg	Neg											Neg						
912	high-burden	Neg	Neg											Neg						
913	relapse	Neg	Neg											Neg						
914	default	Neg	Neg											Neg						
915	relapse	Neg	Neg											Neg						
916	relapse	Neg	Neg											Neg						
917	unknown	3+	Pos	S										Pos	S	S				
918	high-burden	1+	Neg											Neg						

Pt#	Group	AFS	Xpert			DR*plus* (WT absent, MUT binding)		DR*sl* (WT absent, MUT binding)		Sequencing (Discordant)				Convetional results						
			MTBC	RIF	Probe Failure	RIF	INH	FLQ	SLID	RIF	INH	FLQ	SLID	Cul.	INH	RIF	FLQ	KAN	AMK	CAP
919	relapse	1+	Pos	S										Pos	S	S				
920	unknown	1+	Pos	S										Pos	S	S				
921	unknown	1+	Neg											Neg						
922	high-risk	Neg	Neg											Neg						
923	relapse	Neg	Neg											Neg						
924	close contacts	3+	Pos	S										Pos	S	S				
925	failure	1+	Pos	S										Neg						
926	unknown	1+	Pos	S										Neg						
927	unknown	1+	Pos	I										NTM						
928	high-burden	Neg	Neg											Neg						
929	high-burden	Neg	Neg											Neg						
930	high-burden	Neg	Neg											Neg						
931	relapse	Neg	Neg											Neg						
932	relapse	Neg	Neg											NTM						
933	failure	1+	Neg											Neg						
934	unknown	Neg	Pos	S										Pos	S	S				
935	high-burden	Neg	Neg											Neg						
936	relapse	Neg	Neg											Neg						
937	close contacts	Neg	Neg											Neg						
938	high-risk	Neg	Neg											Neg						
939	high-burden	Neg	Neg											Neg						
940	failure	Neg	Neg											Neg						
941	relapse	Neg	Pos	S										Pos	S	S				
942	relapse	Neg	Neg											Neg						
943	relapse	S	Neg											Neg						
944	unknown	1+	Pos	S										Pos	S	S				
945	high-burden	Neg	Neg											Neg						
946	high-burden	Neg	Neg											Neg						

Pt#	Group	AFS	Xpert			DR*plus* (WT absent, MUT binding)		DR*sl* (WT absent, MUT binding)		Sequencing (Discordant)				Convetional results						
			MTBC	RIF	Probe Failure	RIF	INH	FLQ	SLID	RIF	INH	FLQ	SLID	Cul.	INH	RIF	FLQ	KAN	AMK	CAP
947	high-burden	Neg	Neg											Neg						
948	high-burden	S	Neg											Neg						
949	high-burden	Neg	Neg											Neg						
950	high-burden	Neg	Neg											Neg						
951	unknown	Neg	Neg											Neg						
952	failure	S	Pos	R	D	WT7,MUT2A	S							Pos	S	R	S	S	-	S
953	failure	1+	Pos	S										Neg						
954	high-risk	1+	Neg											Neg						
955	high-burden	Neg	Neg											Pos	S	S				
956	failure	Neg	Pos	S										Neg						
957	unknown	2+	Pos	S										Pos	S	S				
958	high-risk	Neg	Neg											NTM						
959	high-risk	Neg	Pos	S										Pos	S	S				
960	high-risk	1+	Pos	S										Pos	R	S				
961	high-risk	2+	Neg											Pos	S	S				
962	high-risk	Neg	Neg											Pos	S	S				
963	high-burden	1+	Neg											Neg						
964	high-risk	1+	Neg											NTM						
965	high-risk	Neg	Neg											Neg						
966	relapse	Neg	Neg											Neg						
967	relapse	Neg	Pos	S										Pos	S	S				
968	unknown	Neg	Neg											Neg						
969	high-burden	Neg	Neg											Neg						
970	unknown	1+	Pos	S										Neg						
971	failure	Neg	Neg											Neg						
972	high-burden	1+	Pos	S										Pos	S	S				
973	unknown	Neg	Neg											Neg						
974	unknown	Neg	Neg											Neg						

Pt#	Group	AFS	Xpert			DR*plus* (WT absent, MUT binding)		DR*sl* (WT absent, MUT binding)		Sequencing (Discordant)				Convetional results						
			MTBC	RIF	Probe Failure	RIF	INH	FLQ	SLID	RIF	INH	FLQ	SLID	Cul.	INH	RIF	FLQ	KAN	AMK	CAP
975	unknown	2+	Pos	S										Neg						
976	unknown	Neg	Pos	S										Neg						
977	unknown	Neg	Neg											NTM						
978	relapse	Neg	Neg											Neg						
979	relapse	Neg	Neg											Neg						
980	relapse	Neg	Neg											Neg						
981	high-burden	Neg	Neg											Neg						
982	failure	Neg	Neg											Neg						
983	unknown	Neg	Neg											Neg						
984	high-risk	Neg	Neg											Neg						
985	relapse	1+	Neg											Pos	S	S				
986	unknown	Neg	Neg											Neg						
987	high-risk	Neg	Neg											Neg						
988	relapse	Neg	Neg											Neg						
989	failure	1+	Neg											Neg						
990	unknown	1+	Neg											Neg						
991	relapse	Neg	Neg											Neg						
992	unknown	Neg	Pos	S										NTM						
993	failure	Neg	Pos	S										Pos	S	S				
994	high-burden	Neg	Neg											Pos	S	S				
995	failure	1+	Pos	S										Pos	S	S				
996	high-burden	Neg	Neg											Neg						
997	failure	1+	Neg											Neg						
998	relapse	Neg	Neg											Neg						
999	high-risk	4+	Pos	S										Pos	S	S				
1000	unknown	Neg	Neg											Neg						
1001	high-burden	Neg	Neg											Pos	S	S				
1002	high-burden	Neg	Neg											Pos	S	S				

Pt#	Group	AFS	Xpert			DR*plus* (WT absent, MUT binding)		DR*sl* (WT absent, MUT binding)		Sequencing (Discordant)				Convetional results						
			MTBC	RIF	Probe Failure	RIF	INH	FLQ	SLID	RIF	INH	FLQ	SLID	Cul.	INH	RIF	FLQ	KAN	AMK	CAP
1003	unknown	Neg	Neg											Pos	R	S				
1004	unknown	Neg	Neg											Neg						
1005	unknown	Neg	Neg											Neg						
1006	close contacts	Neg	Neg											Neg						
1007	failure	Neg	Neg											Neg						
1008	failure	Neg	Neg											Neg						
1009	failure	Neg	Neg											Neg						
1010	relapse	Neg	Neg											Missing						
1011	high-risk	Neg	Neg											Neg						
1012	high-risk	Neg	Neg											Neg						
1013	high-burden	1+	Neg											Neg						
1014	high-risk	4+	Pos	S										Pos	S	S				
1015	relapse	Neg	Neg											Neg						
1016	high-risk	Neg	Pos	S										Neg						
1017	unknown	Neg	Neg											Neg						
1018	unknown	1+	Pos	S										Neg						
1019	unknown	1+	Neg											Neg						
1020	unknown	S	Neg											Neg						
1021	unknown	-	Pos	S										Pos	S	S				
1022	unknown	Neg	Neg											Neg						
1023	relapse	Neg	Neg											Neg						
1024	relapse	Neg	Neg											Neg						
1025	failure	Neg	Pos	S										Neg						
1026	failure	Neg	Pos	S										NTM						
1027	default	Neg	Neg											Neg						
1028	relapse	S	Neg											Neg						
1029	unknown	Neg	Pos	S										Neg						
1030	unknown	Neg	Neg											Neg						

Pt#	Group	AFS	Xpert			DR*plus* (WT absent, MUT binding)		DR*sl* (WT absent, MUT binding)		Sequencing (Discordant)				Convetional results						
			MTBC	RIF	Probe Failure	RIF	INH	FLQ	SLID	RIF	INH	FLQ	SLID	Cul.	INH	RIF	FLQ	KAN	AMK	CAP
1031	unknown	1+	Neg											Neg						
1032	failure	S	Pos	S										Neg						
1033	high-burden	Neg	Pos	R	E	-	-							Missing						
1034	unknown	1+	Pos	S										Pos	S	S				
1035	high-burden	Neg	Neg											Neg						
1036	failure	1+	Neg											Neg						
1037	failure	2+	Pos	S										Pos	S	S				
1038	high-burden	Neg	Neg											Neg						
1039	high-burden	Neg	Neg											Neg						
1040	failure	1+	Pos	R	D	WT7	*katG* WT,MUT1							Pos	R	R	S	S	S	S
1041	high-risk	1+	Pos	S										Pos	S	S				
1042	high-risk	Neg	Neg											Neg						
1043	relapse	1+	Pos	S										Pos	S	S				
1044	failure	S	Pos	S										Pos	S	S				
1045	unknown	1+	Pos	S										Pos	S	S				
1046	failure	Neg	Neg											Neg						
1047	failure	Neg	Neg											NTM						
1048	relapse	Neg	Neg											NTM						
1049	unknown	Neg	Neg											Pos	S	S				
1050	high-burden	S	Pos	S										Pos	S	S				
1051	high-burden	1+	Pos	S										Pos	S	S				
1052	high-burden	Neg	Neg											Neg						
1053	failure	1+	Neg											Neg						
1054	unknown	Neg	Neg											Neg						
1055	failure	2+	Pos	S										NTM						
1056	high-risk	Neg	Neg											Neg						
1057	high-risk	Neg	Neg											Neg						

Pt#	Group	AFS	Xpert			DR*plus* (WT absent, MUT binding)		DR*sl* (WT absent, MUT binding)		Sequencing (Discordant)				Convetional results						
			MTBC	RIF	Probe Failure	RIF	INH	FLQ	SLID	RIF	INH	FLQ	SLID	Cul.	INH	RIF	FLQ	KAN	AMK	CAP
1058	high-burden	Neg	Neg											Neg						
1059	failure	Neg	Pos	S										Pos	S	S				
1060	relapse	Neg	Neg											Neg						
1061	relapse	Neg	Neg											Neg						
1062	failure	1+	Pos	S										Neg						
1063	relapse	Neg	Neg											Neg						
1064	high-risk	Neg	Neg											Pos	S	S				
1065	unknown	Neg	Neg											Neg						
1066	relapse	S	Neg											Neg						
1067	relapse	Neg	Neg											Neg						
1068	high-burden	Neg	Neg											Neg						
1069	relapse	Neg	Neg											Missing						
1070	high-risk	Neg	Neg											Neg						
1071	high-risk	Neg	Neg											NTM						
1072	unknown	Neg	Neg											Neg						
1073	unknown	1+	Pos	S										Pos	S	S				
1074	high-risk	Neg	Neg											Neg						
1075	unknown	Neg	Neg											Neg						
1076	failure	Neg	Neg											Neg						
1077	high-risk	Neg	Neg											Pos	S	S				
1078	relapse	Neg	Neg											Neg						
1079	high-burden	1+	Neg											Neg						
1080	unknown	1+	Pos	S										Neg						
1081	relapse	Neg	Neg											Neg						
1082	high-burden	S	Neg											Neg						
1083	high-burden	Neg	Neg											Neg						
1084	high-burden	Neg	Neg											Neg						
1085	high-burden	Neg	Neg											Pos	S	S				

Pt#	Group	AFS	Xpert			DR*plus* (WT absent, MUT binding)		DR*sl* (WT absent, MUT binding)		Sequencing (Discordant)				Convetional results						
			MTBC	RIF	Probe Failure	RIF	INH	FLQ	SLID	RIF	INH	FLQ	SLID	Cul.	INH	RIF	FLQ	KAN	AMK	CAP
1086	failure	1+	Pos	S										Pos	S	S				
1087	unknown	S	Neg											Neg						
1088	unknown	Neg	Neg											NTM						
1089	failure	Neg	Neg											Neg						
1090	failure	Neg	Neg											Neg						
1091	high-burden	Neg	Neg											Neg						
1092	high-burden	Neg	Neg											Neg						
1093	failure	Neg	Pos	S										Pos	S	S				
1094	relapse	Neg	Neg											Neg						
1095	high-burden	Neg	Pos	S										Neg						
1096	failure	Neg	Neg											Neg						
1097	high-burden	S	Neg											Pos	S	S				
1098	failure	2+	Pos	R	E	WT8,MUT3	S							Pos	S	R	S	S	-	S
1099	high-burden	3+	Pos	S										Pos	R	S				
1100	high-burden	Neg	Neg											Pos	S	S				
1101	failure	2+	Pos	S										Pos	S	S				
1102	failure	-	Pos	S										Neg						
1103	high-risk	Neg	Pos	S										Pos	R	S				
1104	failure	3+	Pos	S										Pos	S	S				
1105	unknown	Neg	Neg											Pos	S	S				
1106	high-risk	4+	Pos	S										Pos	S	S				
1107	failure	Neg	Pos	R	E	WT8,MUT3	*inhA* WT1,MUT1		*rrs* MUT2				*rrs* WT eisWT	Pos	R	R	S	S	S	S
1108	high-risk	2+	Neg											Pos	S	R	S	S	-	S
1109	failure	S	Pos	S										Neg						
1110	high-burden	Neg	Neg											Neg						
1111	high-burden	3+	Pos	S										Pos	S	S				
1112	unknown	1+	Pos	S										Neg						
1113	high-risk	Neg	Neg											Neg						

Pt#	Group	AFS	Xpert			DR*plus* (WT absent, MUT binding)		DR*sl* (WT absent, MUT binding)		Sequencing (Discordant)				Convetional results						
			MTBC	RIF	Probe Failure	RIF	INH	FLQ	SLID	RIF	INH	FLQ	SLID	Cul.	INH	RIF	FLQ	KAN	AMK	CAP
1114	failure	Neg	Neg											Neg						
1115	unknown	Neg	Neg											Neg						
1116	high-burden	Neg	Neg											Pos	S	S				
1117	high-burden	Neg	Neg											Neg						
1118	unknown	1+	Pos	S										Pos	S	S				
1119	relapse	2+	Pos	S										Pos	S	S				
1120	unknown	1+	Pos	S										Pos	S	S				
1121	relapse	Neg	Neg											Neg						
1122	unknown	S	Pos	S										Neg						
1123	relapse	Neg	Neg											Neg						
1124	high-burden	Neg	Neg											Neg						
1125	relapse	Neg	Neg											Neg						
1126	unknown	Neg	Pos	S										Neg						
1127	failure	Neg	Neg											Neg						
1128	unknown	Neg	Neg											Neg						
1129	high-burden	Neg	Neg											Pos	S	S				
1130	relapse	Neg	Neg											NTM						
1131	unknown	Neg	Neg											Neg						
1132	high-burden	Neg	Neg											Neg						
1133	failure	1+	Neg											Neg						
1134	unknown	Neg	Pos	S										Neg						
1135	high-risk	S	Neg											Neg						
1136	relapse	S	Pos	S										Neg						
1137	relapse	Neg	Neg											Neg						
1138	high-risk	Neg	Neg											Neg						
1139	relapse	Neg	Pos	S										NTM						
1140	high-burden	Neg	Neg											Pos	S	S				
1141	unknown	Neg	Neg											Neg						

Pt#	Group	AFS	Xpert			DR*plus* (WT absent, MUT binding)		DR*sl* (WT absent, MUT binding)		Sequencing (Discordant)				Convetional results						
			MTBC	RIF	Probe Failure	RIF	INH	FLQ	SLID	RIF	INH	FLQ	SLID	Cul.	INH	RIF	FLQ	KAN	AMK	CAP
1142	unknown	1+	Pos	S										Pos	S	S				
1143	unknown	1+	Pos	S										Pos	S	S				
1144	unknown	S	Pos	S										Neg						
1145	high-burden	1+	Neg											Neg						
1146	high-burden	Neg	Neg											Neg						
1147	high-burden	Neg	Neg											Neg						
1148	high-risk	4+	Pos	S										Pos	S	S				
1149	relapse	Neg	Pos	S										Neg						
1150	failure	Neg	Neg											Neg						
1151	default	Neg	Neg											Neg						
1152	high-risk	Neg	Neg											Neg						
1153	high-risk	Neg	Neg											Neg						
1154	high-burden	Neg	Neg											Neg						
1155	failure	1+	Neg											Neg						
1156	relapse	Neg	Neg											Neg						
1157	high-risk	S	Neg											Neg						
1158	failure	1+	Pos	S										Pos	S	S				
1159	high-risk	Neg	Neg											Neg						
1160	relapse	Neg	Pos	S										Neg						
1161	failure	2+	Pos	S										Pos	S	S				
1162	failure	1+	Pos	S										Neg						
1163	high-burden	Neg	Neg											Pos	R	R	S	S	S	S
1164	relapse	Neg	Neg											Neg						
1165	unknown	-	Neg											NTM						
1166	unknown	2+	Neg											Neg						
1167	failure	1+	Pos	S										Neg						
1168	high-burden	Neg	Pos	S										Pos	S	S				
1169	failure	S	Neg											Neg						

Pt#	Group	AFS	Xpert			DR*plus* (WT absent, MUT binding)		DR*sl* (WT absent, MUT binding)		Sequencing (Discordant)				Convetional results						
			MTBC	RIF	Probe Failure	RIF	INH	FLQ	SLID	RIF	INH	FLQ	SLID	Cul.	INH	RIF	FLQ	KAN	AMK	CAP
1170	high-burden	Neg	Neg											Neg						
1171	high-burden	Neg	Neg											Neg						
1172	high-burden	Neg	Neg											Neg						
1173	high-risk	Neg	Neg											Neg						
1174	relapse	1+	Neg											NTM						
1175	unknown	S	Neg											Neg						
1176	unknown	Neg	Neg											Neg						
1177	relapse	Neg	Neg											Neg						
1178	relapse	Neg	Neg											Neg						
1179	high-burden	1+	Pos	S										Pos	S	S				
1180	high-burden	1+	Pos	S										Pos	S	S				
1181	high-risk	Neg	Neg											Pos	S	S				
1182	high-burden	Neg	Neg											Neg						
1183	failure	1+	Pos	S										Neg						
1184	high-burden	Neg	Neg											Neg						
1185	high-burden	Neg	Pos	S										Pos	S	S				
1186	high-risk	2+	Pos	S										Pos	S	S				
1187	relapse	Neg	I											Pos	S	S				
1188	relapse	Neg	Neg											Neg						
1189	relapse	Neg	Neg											Neg						
1190	high-risk	3+	Pos	S										Pos	R	S				
1191	unknown	-	Neg											NTM						
1192	relapse	Neg	Neg											Neg						
1193	high-burden	Neg	Neg											Neg						
1194	high-burden	Neg	Neg											Neg						
1195	high-burden	Neg	Neg											Neg						
1196	high-burden	Neg	Neg											Neg						
1197	high-burden	Neg	Neg											Neg						

Pt#	Group	AFS	Xpert			DR*plus* (WT absent, MUT binding)		DR*sl* (WT absent, MUT binding)		Sequencing (Discordant)				Convetional results						
			MTBC	RIF	Probe Failure	RIF	INH	FLQ	SLID	RIF	INH	FLQ	SLID	Cul.	INH	RIF	FLQ	KAN	AMK	CAP
1198	failure	2+	Pos	S										Pos	S	S				
1199	relapse	Neg	Neg											Neg						
1200	high-burden	Neg	Neg											Pos	S	S				
1201	unknown	Neg	Neg											Neg						
1202	high-burden	Neg	Neg											Neg						
1203	high-burden	Neg	Neg											Neg						
1204	relapse	2+	Neg											Neg						
1205	high-burden	Neg	Pos	S										Neg						
1206	high-burden	Neg	Neg											Neg						
1207	relapse	Neg	Neg											Neg						
1208	high-burden	3+	Pos	S										Pos	S	S				
1209	close contacts	Neg	Neg											Neg						
1210	high-burden	Neg	Neg											Neg						
1211	unknown	Neg	Neg											Pos	S	S				
1212	high-burden	Neg	Neg											Neg						
1213	high-burden	Neg	Neg											Neg						
1214	high-burden	Neg	Neg											Neg						
1215	high-risk	2+	Neg											Neg						
1216	relapse	Neg	Neg											Neg						
1217	high-burden	Neg	Neg											Neg						
1218	unknown	1+	Pos	R	E	-	-							Neg						
1219	failure	2+	Pos	S										Pos	S	S				
1220	failure	Neg	Pos	S										Neg						
1221	relapse	Neg	Neg											Neg						
1222	high-burden	Neg	Pos	S										Pos	R	S				
1223	unknown	Neg	Neg											Pos	S	S				
1224	failure	2+	Neg											Neg						
1225	unknown	Neg	Neg											Neg						

Pt#	Group	AFS	Xpert			DR*plus* (WT absent, MUT binding)		DR*sl* (WT absent, MUT binding)		Sequencing (Discordant)				Convetional results						
			MTBC	RIF	Probe Failure	RIF	INH	FLQ	SLID	RIF	INH	FLQ	SLID	Cul.	INH	RIF	FLQ	KAN	AMK	CAP
1226	unknown	1+	Pos	S										Pos	S	S				
1227	high-burden	Neg	Neg											Neg						
1228	high-risk	Neg	Neg											Pos	S	S				
1229	high-risk	4+	Pos	S										Pos	S	S				
1230	high-burden	S	Neg											NTM						
1231	unknown	Neg	Pos	S										Neg						
1232	failure	1+	Pos	S										Neg						
1233	relapse	Neg	Neg											Neg						
1234	relapse	Neg	Neg											Neg						
1235	high-burden	Neg	Neg											Neg						
1236	high-burden	Neg	Neg											Neg						
1237	high-burden	Neg	Neg											Neg						
1238	high-risk	Neg	Neg											Neg						
1239	high-burden	Neg	Neg											Pos	S	S				
1240	unknown	1+	Neg											Neg						
1241	default	Neg	Neg											Neg						
1242	high-burden	2+	Pos	S										Pos	S	S				
1243	default	3+	Pos	S										Pos	R	S				
1244	high-burden	Neg	Neg											Neg						
1245	high-burden	Neg	Neg											Pos	S	S				
1246	high-burden	Neg	Neg											Neg						
1247	high-burden	Neg	Pos	S										Pos	R	S				
1248	high-burden	Neg	Neg											Neg						
1249	high-burden	Neg	Neg											Neg						
1250	high-burden	S	Pos	S										Pos	S	S				
1251	high-burden	-	Neg											Neg						
1252	unknown	1+	Neg											Pos	R	S				
1253	high-burden	Neg	Neg											Neg						

Pt#	Group	AFS	Xpert			DR*plus* (WT absent, MUT binding)		DR*sl* (WT absent, MUT binding)		Sequencing (Discordant)				Convetional results						
			MTBC	RIF	Probe Failure	RIF	INH	FLQ	SLID	RIF	INH	FLQ	SLID	Cul.	INH	RIF	FLQ	KAN	AMK	CAP
1254	relapse	Neg	Neg											Neg						
1255	high-burden	Neg	Neg											Neg						
1256	high-burden	Neg	Neg											Neg						
1257	failure	Neg	Pos	R	A	-	-			L511P				Pos	S	S	S	S	-	S
1258	failure	Neg	Neg											Neg						
1259	unknown	Neg	Neg											Neg						
1260	relapse	1+	Pos	S										Pos	S	S				
1261	unknown	Neg	Neg											Neg						
1262	relapse	Neg	Neg											Pos	R	S				
1263	high-burden	Neg	Neg											Neg						
1264	unknown	Neg	Neg											Neg						
1265	high-burden	Neg	Neg											Neg						
1266	failure	S	Pos	S										Pos	S	S				
1267	high-burden	Neg	Neg											Neg						
1268	unknown	Neg	Neg											Neg						
1269	failure	1+	Neg											NTM						
1270	high-burden	Neg	Neg											Neg						
1271	relapse	4+	Pos	S										Pos	R	S				
1272	relapse	Neg	Neg											Neg						
1273	relapse	Neg	Neg											Neg						
1274	high-burden	Neg	Neg											Pos	S	S				
1275	unknown	1+	Neg											Neg						
1276	relapse	1+	Neg											Neg						
1277	high-burden	Neg	Neg											Neg						
1278	high-burden	Neg	Neg											Neg						
1279	relapse	Neg	Neg											Neg						
1280	failure	1+	Neg											Neg						
1281	failure	S	Pos	S										Neg						

Pt#	Group	AFS	Xpert			DR*plus* (WT absent, MUT binding)		DR*sl* (WT absent, MUT binding)		Sequencing (Discordant)				Convetional results						
			MTBC	RIF	Probe Failure	RIF	INH	FLQ	SLID	RIF	INH	FLQ	SLID	Cul.	INH	RIF	FLQ	KAN	AMK	CAP
1282	relapse	1+	Neg											NTM						
1283	failure	1+	Neg											Neg						
1284	relapse	Neg	Neg											Neg						
1285	relapse	S	Neg											NTM						
1286	failure	4+	Pos	S										Pos	S	S	S	S	-	S
1287	relapse	1+	Pos	S										NTM						
1288	relapse	Neg	Neg											Neg						
1289	failure	Neg	Pos	S										Pos	S	S				
1290	failure	S	Pos	S										Pos	S	S				
1291	relapse	4+	Pos	R	E	WT8,MUT3	*katG* WT,MUT1							Pos	R	R	S	S	S	S
1292	high-burden	1+	Pos	S										Pos	S	S				
1293	relapse	4+	Pos	S										Pos	S	S				
1294	failure	S	Neg											NTM						
1295	relapse	S	Pos	S										NTM						
1296	relapse	Neg	Neg											Neg						
1297	relapse	3+	Neg											Neg						
1298	relapse	1+	Neg											NTM						
1299	relapse	4+	Pos	S										NTM						
1300	failure	3+	Neg											NTM						
1301	high-burden	1+	Pos	S										Pos	S	S				
1302	close contacts	Neg	Neg											Neg						
1303	relapse	3+	Pos	S										Pos	S	S				
1304	relapse	S	Neg											Neg						
1305	failure	1+	Neg											Neg						
1306	failure	3+	Pos	S										Pos	S	S				
1307	relapse	2+	Pos	S										Neg						
1308	failure	1+	Pos	S										Neg						
1309	relapse	1+	Pos	S										Pos	S	S				

Pt#	Group	AFS	Xpert			DR*plus* (WT absent, MUT binding)		DR*sl* (WT absent, MUT binding)		Sequencing (Discordant)				Convetional results						
			MTBC	RIF	Probe Failure	RIF	INH	FLQ	SLID	RIF	INH	FLQ	SLID	Cul.	INH	RIF	FLQ	KAN	AMK	CAP
1310	relapse	2+	Pos	S										Pos	S	S				
1311	high-burden	4+	Pos	S										Pos	S	S				
1312	close contacts	Neg	Neg											Pos	R	R	S	S	S	S
1313	high-burden	1+	Pos	S										Pos	S	S				
1314	high-burden	Neg	Neg											Pos	S	S				
1315	relapse	Neg	Neg											Neg						
1316	relapse	2+	Pos	S										Pos	R	S				
1317	failure	1+	Pos	S										Pos	S	S				
1318	relapse	2+	Neg											NTM						
1319	relapse	4+	Neg											NTM						
1320	high-burden	Neg	Neg											Pos	S	S				
1321	relapse	4+	Pos	S										Pos	S	S				
1322	failure	1+	Neg											NTM						
1323	high-risk	1+	Pos	S										Pos	S	S				
1324	high-burden	Neg	Neg											Pos	S	S				
1325	high-burden	2+	Pos	R	E	WT8,MUT3	*inhA* WT1,MUT1		*rrs* WT1,MUT 1					Pos	R	R	S	R	R	R
1326	high-burden	1+	Pos	S										Pos	S	S				
1327	failure	1+	Pos	R	E	WT8	S		*rrs* WT1,WT2, MUT2					Pos	S	R	-	-	-	-
1328	failure	1+	Neg											NTM						
1329	relapse	1+	Neg											Neg						
1330	high-burden	3+	Pos	S										Pos	S	S				
1331	high-risk	Neg	Neg											Neg						
1332	high-burden	Neg	Pos	S										Neg						
1333	relapse	Neg	Pos	S										Pos	S	S				
1334	high-burden	Neg	Neg											Neg						
1335	high-burden	Neg	Neg											Pos	S	S				

Pt#	Group	AFS	Xpert			DR*plus* (WT absent, MUT binding)		DR*sl* (WT absent, MUT binding)		Sequencing (Discordant)				Convetional results						
			MTBC	RIF	Probe Failure	RIF	INH	FLQ	SLID	RIF	INH	FLQ	SLID	Cul.	INH	RIF	FLQ	KAN	AMK	CAP
1336	high-burden	2+	Pos	R	D	WT7,MUT2B	S							Pos	S	R	S	S	S	S
1337	relapse	Neg	Pos	R	B	WT3,WT4	*katG* WT,MUT1							Pos	R	R	S	S	S	S
1338	relapse	Neg	Neg											Pos	S	R	S	S	-	S
1339	relapse	3+	Pos	S										Pos	S	S				
1340	relapse	Neg	Neg											Neg						
1341	high-burden	Neg	Neg											Pos	S	S				
1342	relapse	1+	Pos	R	E	WT8,MUT3	S							Pos	S	R	S	S	-	S
1343	high-burden	Neg	Neg											Neg						
1344	failure	1+	Pos	S										Neg						
1345	failure	Neg	Pos	S										Pos	S	S				
1346	high-burden	Neg	Neg											Neg						
1347	high-burden	Neg	Pos	S										Neg						
1348	high-burden	Neg	Pos	S										Pos	S	S				
1349	high-burden	Neg	Neg											Neg						
1350	relapse	1+	Pos	R	B	WT3,WT4	S			D516Y				Pos	S	S	S	S	-	S
1351	failure	3+	Pos	S										Pos	S	S				
1352	high-burden	Neg	Neg											Missing						
1353	high-burden	Neg	Neg											Neg						
1354	high-burden	Neg	Neg											Neg						
1355	relapse	2+	Pos	S										Pos	S	S				
1356	failure	2+	Pos	S										Neg						
1357	relapse	Neg	Neg											Neg						
1358	relapse	2+	Neg											Neg						
1359	high-burden	1+	Pos	S										Pos	S	S				
1360	relapse	4+	Pos	S										Pos	S	S				
1361	high-burden	2+	Pos	S										Pos	S	S				
1362	failure	S	Neg											Neg						
1363	high-burden	Neg	Neg											Pos	S	S				

Pt#	Group	AFS	Xpert			DR*plus* (WT absent, MUT binding)		DR*sl* (WT absent, MUT binding)		Sequencing (Discordant)				Convetional results						
			MTBC	RIF	Probe Failure	RIF	INH	FLQ	SLID	RIF	INH	FLQ	SLID	Cul.	INH	RIF	FLQ	KAN	AMK	CAP
1364	high-burden	Neg	Pos	S										Pos	S	S				
1365	relapse	Neg	Neg											Neg						
1366	failure	1+	Neg											Neg						
1367	relapse	1+	Pos	S										Pos	S	S				
1368	relapse	1+	Pos	S										Pos	S	S				
1369	relapse	1+	Pos	S										Pos	R	S				
1370	failure	2+	Pos	S										Pos	R	S	S	S	-	S
1371	failure	S	Pos	S										Pos	S	S				
1372	relapse	2+	Pos	S										NTM						
1373	relapse	2+	Pos	S										Pos	S	S				
1374	relapse	Neg	Neg											Missing						
1375	failure	1+	Pos	S										Neg						
1376	high-risk	Neg	Neg											Neg						
1377	high-burden	Neg	Neg											Neg						
1378	relapse	Neg	Neg											Pos	S	S				
1379	failure	S	Pos	S										Pos	S	S				
1380	relapse	Neg	Neg											Neg						
1381	failure	Neg	Neg											Neg						
1382	relapse	2+	Pos	S										Pos	S	S				
1383	high-burden	Neg	Neg											Neg						
1384	high-burden	Neg	Neg											Neg						
1385	relapse	Neg	Neg											Neg						
1386	high-burden	Neg	Neg											Neg						
1387	failure	S	Neg											Neg						
1388	failure	S	Pos	S										Neg						
1389	relapse	Neg	Pos	S										Neg						
1390	high-burden	2+	Pos	S										Pos	S	S				
1391	relapse	4+	Pos	S										Pos	S	S				

Pt#	Group	AFS	Xpert			DR*plus* (WT absent, MUT binding)		DR*sl* (WT absent, MUT binding)		Sequencing (Discordant)				Convetional results						
			MTBC	RIF	Probe Failure	RIF	INH	FLQ	SLID	RIF	INH	FLQ	SLID	Cul.	INH	RIF	FLQ	KAN	AMK	CAP
1392	failure	1+	Pos	S										Neg						
1393	high-risk	Neg	Pos	S										Pos	S	S				
1394	high-risk	4+	Pos	S										Pos	S	S				
1395	high-burden	Neg	Neg											Neg						
1396	high-burden	Neg	Neg											Pos	S	S				
1397	failure	2+	Pos	S										Pos	S	S				
1398	high-burden	1+	Pos	S										Pos	S	S				
1399	relapse	S	Neg											Pos	S	S				
1400	relapse	1+	Pos	S										Neg						
1401	high-risk	Neg	Neg											Neg						
1402	failure	3+	Pos	S										Pos	S	S				
1403	failure	1+	Pos	S										Pos	S	S				
1404	high-burden	Neg	Neg											Neg						
1405	high-burden	1+	Pos	S										Pos	S	S				
1406	failure	S	Neg											Neg						
1407	high-burden	Neg	Neg											Neg						
1408	high-burden	Neg	Neg											Neg						
1409	high-risk	Neg	Neg											Neg						
1410	relapse	S	Neg											Neg						
1411	relapse	Neg	Neg											Neg						
1412	failure	1+	Pos	S										Neg						
1413	high-risk	Neg	Neg											Pos	S	S				
1414	high-burden	S	Pos	S										Pos	S	S				
1415	failure	4+	Neg											NTM						
1416	high-risk	Neg	Neg											Neg						
1417	relapse	1+	Pos	S										Neg						
1418	high-burden	1+	Pos	S										Pos	S	S				
1419	default	S	Neg											NTM						

Pt#	Group	AFS	Xpert			DR*plus* (WT absent, MUT binding)		DR*sl* (WT absent, MUT binding)		Sequencing (Discordant)				Convetional results						
			MTBC	RIF	Probe Failure	RIF	INH	FLQ	SLID	RIF	INH	FLQ	SLID	Cul.	INH	RIF	FLQ	KAN	AMK	CAP
1420	high-burden	Neg	Neg											Neg						
1421	high-burden	Neg	Neg											Neg						
1422	failure	2+	Neg											Neg						
1423	relapse	S	Pos	S										Pos	R	S				
1424	relapse	S	Neg											NTM						
1425	failure	S	Pos	S										Neg						
1426	failure	1+	Pos	S										Pos	S	S	S	S	-	S
1427	relapse	S	Pos	S										Neg						
1428	high-risk	2+	Pos	S										Pos	S	S				
1429	high-burden	2+	Pos	S										Pos	R	S				
1430	high-burden	1+	Neg											Pos	S	S				
1431	failure	S	Neg											Neg						
1432	high-burden	Neg	Neg											Pos	R	S				
1433	high-burden	Neg	Neg											Neg						
1434	failure	2+	Pos	S										Pos	S	S				
1435	relapse	3+	Pos	S										Pos	S	S				
1436	high-burden	1+	Pos	S										Pos	S	S				
1437	high-burden	4+	Pos	S										Pos	S	S				
1438	high-burden	Neg	Pos	S										Pos	S	S				
1439	failure	3+	Pos	S										Pos	S	S				
1440	relapse	2+	Pos	S										Pos	S	S				
1441	high-risk	1+	Pos	S										Pos	S	S				
1442	failure	1+	Pos	S										Pos	S	S				
1443	failure	1+	Pos	S										Pos	S	S				
1444	relapse	1+	Neg											Neg						
1445	high-burden	Neg	Neg											Neg						
1446	close contacts	Neg	Neg											Neg						
1447	high-burden	1+	Neg											Neg						

Pt#	Group	AFS	Xpert			DR*plus* (WT absent, MUT binding)		DR*sl* (WT absent, MUT binding)		Sequencing (Discordant)				Convetional results						
			MTBC	RIF	Probe Failure	RIF	INH	FLQ	SLID	RIF	INH	FLQ	SLID	Cul.	INH	RIF	FLQ	KAN	AMK	CAP
1448	failure	S	Neg											Neg						
1449	high-burden	Neg	Neg											Neg						
1450	high-burden	S	Neg											Pos	S	S				
1451	relapse	Neg	Neg											Neg						
1452	high-risk	4+	Pos	S										Pos	S	S				
1453	high-risk	Neg	Neg											Neg						
1454	high-risk	Neg	Neg											Neg						
1455	high-burden	S	Pos	S										Pos	S	S				
1456	relapse	Neg	Pos	S										Pos	S	S				
1457	failure	1+	Neg											NTM						
1458	default	S	Pos	S										Pos	S	S				
1459	relapse	4+	Pos	S										Pos	S	S				
1460	high-risk	Neg	Neg											Neg						
1461	high-burden	3+	Pos	S										Pos	S	S				
1462	relapse	Neg	Neg											Neg						
1463	relapse	S	Neg											Neg						
1464	high-risk	1+	Pos	S										Pos	S	S				
1465	relapse	S	Pos	S										Pos	S	S				
1466	failure	2+	Pos	S										NTM						
1467	relapse	S	Neg											NTM						
1468	high-burden	S	Pos	S										Pos	S	S				
1469	high-burden	Neg	Neg											Pos	S	S				
1470	relapse	Neg	Neg											Pos	S	S				
1471	high-risk	4+	Pos	S										Pos	S	S				
1472	relapse	2+	Pos	S										Pos	S	S				
1473	relapse	2+	Neg											Neg						
1474	relapse	4+	Pos	S										Pos	S	S				
1475	relapse	S	Pos	R	A	WT2	*katG* WT,MUT1							Pos	R	R	S	S	S	S

Pt#	Group	AFS	Xpert			DR*plus* (WT absent, MUT binding)		DR*sl* (WT absent, MUT binding)		Sequencing (Discordant)				Convetional results						
			MTBC	RIF	Probe Failure	RIF	INH	FLQ	SLID	RIF	INH	FLQ	SLID	Cul.	INH	RIF	FLQ	KAN	AMK	CAP
1476	relapse	4+	Neg											NTM						
1477	relapse	S	Neg											NTM						
1478	high-burden	Neg	Neg											Neg						
1479	high-burden	Neg	Neg											Neg						
1480	relapse	3+	Neg											Neg						
1481	high-burden	Neg	Neg											Neg						
1482	relapse	Neg	Neg											Neg						
1483	failure	S	Pos	S										Neg						
1484	relapse	1+	Pos	S										Pos	S	S				
1485	relapse	Neg	Pos	S										Pos	S	S				
1486	relapse	1+	Pos	S										Pos	S	S				
1487	failure	1+	Pos	S										Neg						
1488	relapse	1+	Neg											Pos	S	S				
1489	high-burden	Neg	Pos	S										Pos	S	S				
1490	relapse	2+	Pos	S										Pos	-	S				
1491	relapse	1+	Neg											Neg						
1492	default	Neg	Neg											Pos	S	S				
1493	high-burden	Neg	Neg											Neg						
1494	failure	2+	Pos	S										Pos	R	S				
1495	relapse	3+	Pos	S										Pos	S	S				
1496	relapse	1+	Pos	S										Pos	S	S				
1497	high-risk	Neg	Neg											Neg						
1498	high-burden	Neg	Neg											Neg						
1499	relapse	Neg	Neg											NTM						
1500	high-burden	Neg	Neg											Neg						
1501	relapse	Neg	Neg											Neg						
1502	high-burden	1+	Neg											Neg						
1503	relapse	S	Pos	S										Neg						

Pt#	Group	AFS	Xpert			DR*plus* (WT absent, MUT binding)		DR*sl* (WT absent, MUT binding)		Sequencing (Discordant)				Convetional results						
			MTBC	RIF	Probe Failure	RIF	INH	FLQ	SLID	RIF	INH	FLQ	SLID	Cul.	INH	RIF	FLQ	KAN	AMK	CAP
1504	high-risk	Neg	Pos	S										Pos	S	S				
1505	high-burden	Neg	Neg											NTM						
1506	high-burden	2+	Pos	S										Pos	S	S				
1507	relapse	1+	Pos	S										Pos	S	S				
1508	close contacts	S	Neg											Pos	S	S				
1509	high-burden	Neg	Neg											Neg						
1510	relapse	1+	Neg											NTM						
1511	high-burden	Neg	Neg											NTM						
1512	relapse	1+	Neg											NTM						
1513	relapse	Neg	Neg											NTM						
1514	relapse	Neg	Neg											NTM						
1515	high-burden	Neg	Neg											Pos	S	S				
1516	relapse	2+	Neg											Neg						
1517	high-burden	2+	Pos	S										Pos	S	S				
1518	close contacts	Neg	Neg											Neg						
1519	relapse	Neg	Neg											Neg						
1520	high-burden	Neg	Neg											Pos	S	S				
1521	relapse	Neg	Neg											Neg						
1522	relapse	Neg	Neg											Pos	S	S				
1523	high-burden	2+	Pos	S										Pos	S	S				
1524	relapse	4+	Pos	R	E	WT8,MUT3	S				*OxyRahpC* C15T			Pos	R	R	S	S	S	S
1525	high-burden	Neg	Neg											Pos	-	S				
1526	relapse	S	Neg											NTM						
1527	failure	1+	Pos	S										Neg						
1528	high-burden	Neg	Neg											Neg						
1529	default	Neg	Neg											Neg						
1530	high-burden	Neg	Neg											Neg						

Pt#	Group	AFS	Xpert			DR*plus* (WT absent, MUT binding)		DR*sl* (WT absent, MUT binding)		Sequencing (Discordant)				Convetional results						
			MTBC	RIF	Probe Failure	RIF	INH	FLQ	SLID	RIF	INH	FLQ	SLID	Cul.	INH	RIF	FLQ	KAN	AMK	CAP
1531	failure	1+	Pos	S										Pos	-	S				
1532	relapse	Neg	Neg											Neg						
1533	failure	1+	Pos	S										Neg						
1534	failure	1+	Pos	S										Neg						
1535	relapse	1+	Pos	S										Neg						
1536	failure	Neg	Pos	S										Pos	S	S				
1537	high-burden	Neg	Neg											Pos	S	S				
1538	high-burden	3+	Neg											NTM						
1539	high-burden	Neg	Neg											Neg						
1540	high-burden	Neg	Neg											Neg						
1541	failure	1+	Pos	S										Neg						
1542	high-burden	Neg	Pos	S										Pos	S	S				
1543	failure	3+	Pos	S										Pos	S	S				
1544	high-burden	Neg	Neg											Neg						
1545	relapse	Neg	Neg											Neg						
1546	relapse	Neg	Neg											Pos	S	S				
1547	relapse	Neg	Neg											Neg						
1548	high-burden	Neg	Neg											Neg						
1549	relapse	2+	Pos	S										Neg						
1550	relapse	1+	Pos	S										Pos	S	S				
1551	relapse	2+	Pos	S										Pos	S	S				
1552	failure	1+	Neg											NTM						
1553	relapse	1+	Pos	S										Neg						
1554	failure	Neg	Pos	S										Neg						
1555	relapse	Neg	Neg											Neg						
1556	relapse	1+	Neg											Neg						
1557	high-risk	Neg	Neg											Neg						
1558	relapse	3+	Pos	S										Pos	S	S				

Pt#	Group	AFS	Xpert			DR*plus* (WT absent, MUT binding)		DR*sl* (WT absent, MUT binding)		Sequencing (Discordant)				Convetional results						
			MTBC	RIF	Probe Failure	RIF	INH	FLQ	SLID	RIF	INH	FLQ	SLID	Cul.	INH	RIF	FLQ	KAN	AMK	CAP
1559	high-burden	Neg	Neg											Pos	R	S				
1560	relapse	Neg	Neg											Neg						
1561	relapse	Neg	Neg											Neg						
1562	high-burden	Neg	Neg											Pos	S	S	S	S	-	S
1563	high-burden	2+	Pos	S										Pos	S	S				
1564	relapse	S	Neg											NTM						
1565	failure	1+	Pos	S										Neg						
1566	high-burden	Neg	Neg											Pos	S	S				
1567	relapse	1+	Pos	S										Pos	S	S				
1568	failure	Neg	Neg											Neg						
1569	high-burden	1+	Pos	S										Pos	S	S				
1570	high-burden	Neg	Neg											Pos	S	R	S	S	S	S
1571	failure	1+	Pos	S										NTM						
1572	high-burden	Neg	Neg											Neg						
1573	high-burden	Neg	Neg											Neg						
1574	failure	Neg	Pos	S										Neg						
1575	relapse	1+	Pos	S										Pos	S	S				
1576	high-burden	Neg	Pos	S										Pos	S	S				
1577	high-risk	1+	Pos	S										Pos	S	S				
1578	relapse	Neg	Neg											Neg						
1579	relapse	Neg	Neg											Pos	S	S				
1580	high-burden	Neg	Neg											Neg						
1581	default	3+	Neg											NTM						
1582	failure	Neg	Pos	S										Pos	S	S				
1583	high-burden	Neg	Neg											Neg						
1584	high-burden	Neg	Neg											NTM						
1585	high-burden	1+	Pos	S										Pos	S	S	S	S	-	S
1586	relapse	1+	Neg											Neg						

Pt#	Group	AFS	Xpert			DR*plus* (WT absent, MUT binding)		DR*sl* (WT absent, MUT binding)		Sequencing (Discordant)				Convetional results						
			MTBC	RIF	Probe Failure	RIF	INH	FLQ	SLID	RIF	INH	FLQ	SLID	Cul.	INH	RIF	FLQ	KAN	AMK	CAP
1587	relapse	Neg	Neg											Neg						
1588	high-burden	Neg	Neg											Neg						
1589	relapse	Neg	Neg											Neg						
1590	high-burden	Neg	Neg											Neg						
1591	close contacts	S	Pos	I										Pos	R	R	S	S	S	S
1592	high-risk	S	Pos	S										Pos	S	S				
1593	high-burden	Neg	Neg											Pos	S	S				
1594	high-burden	2+	Pos	S										Pos	S	S				
1595	default	Neg	Neg											Neg						
1596	high-burden	Neg	Neg											Neg						
1597	high-burden	Neg	Neg											Pos	S	S				
1598	high-burden	Neg	Neg											Neg						
1599	high-burden	Neg	Neg											Neg						
1600	high-burden	Neg	Neg											Neg						
1601	relapse	1+	Pos	S										Neg						
1602	relapse	1+	Neg											NTM						
1603	high-risk	Neg	Pos	S										Pos	S	S				
1604	relapse	Neg	Neg											Neg						
1605	failure	1+	Pos	S						S531L				Neg						
1606	relapse	1+	Pos	S										Neg						
1607	high-risk	4+	Pos	S										Pos	S	S				
1608	failure	2+	Pos	S										Pos	S	S				
1609	relapse	Neg	Neg											Neg						
1610	high-burden	Neg	Neg											Pos	S	S				
1611	failure	2+	Pos	S										NTM						
1612	relapse	Neg	Neg											Neg						
1613	failure	1+	Neg											Missing						
1614	relapse	4+	Neg											NTM						

Pt#	Group	AFS	Xpert			DR*plus* (WT absent, MUT binding)		DR*sl* (WT absent, MUT binding)		Sequencing (Discordant)				Convetional results						
			MTBC	RIF	Probe Failure	RIF	INH	FLQ	SLID	RIF	INH	FLQ	SLID	Cul.	INH	RIF	FLQ	KAN	AMK	CAP
1615	relapse	Neg	Neg											Neg						
1616	high-burden	Neg	Neg											Neg						
1617	relapse	2+	Pos	S										Neg						
1618	high-risk	Neg	Pos	S										Pos	S	S				
1619	failure	1+	Pos	S										Neg						
1620	failure	1+	Neg											Neg						
1621	default	Neg	Neg											Neg						
1622	relapse	Neg	Neg											Pos	S	S				
1623	failure	S	Pos	S										Neg						
1624	relapse	Neg	Neg											Neg						
1625	failure	S	Neg											Neg						
1626	high-burden	Neg	Neg											Neg						
1627	relapse	Neg	Neg											Neg						
1628	relapse	4+	Pos	S										Pos	S	S				
1629	relapse	Neg	Neg											Neg						
1630	high-burden	2+	Pos	S										Pos	S	S				
1631	high-risk	Neg	Neg											NTM						
1632	relapse	S	Neg											Neg						
1633	default	1+	Pos	S										Neg						
1634	relapse	Neg	Neg											Neg						
1635	high-burden	Neg	Neg											Neg						
1636	high-burden	Neg	Pos	S										Pos	S	S				
1637	high-burden	S	Neg											Neg						
1638	high-burden	Neg	Neg											Neg						
1639	high-burden	Neg	Neg											Neg						
1640	default	Neg	Pos	S										Neg						
1641	default	Neg	Neg											Neg						
1642	failure	Neg	Neg											Neg						

Pt#	Group	AFS	Xpert			DR*plus* (WT absent, MUT binding)		DR*sl* (WT absent, MUT binding)		Sequencing (Discordant)				Convetional results						
			MTBC	RIF	Probe Failure	RIF	INH	FLQ	SLID	RIF	INH	FLQ	SLID	Cul.	INH	RIF	FLQ	KAN	AMK	CAP
1643	relapse	1+	Pos	S										Pos	S	S				
1644	relapse	3+	Pos	R	B	WT3,WT4,MUT1	*katG* WT,MUT1						*rrs* WT<br>*eis* WT	Pos	R	R	S	S	S	R
1645	high-burden	Neg	Neg											Neg						
1646	high-burden	Neg	Neg											Neg						
1647	relapse	Neg	Pos	S										Pos	S	S				
1648	relapse	1+	Neg											Pos	S	S				
1649	default	Neg	Neg											Neg						
1650	failure	1+	Pos	S										Neg						
1651	failure	1+	Pos	S										NTM						
1652	failure	3+	Pos	S										Pos	S	S				
1653	high-burden	Neg	Neg											Neg						
1654	high-burden	Neg	Neg											Neg						
1655	failure	Neg	Pos	S										Neg						
1656	high-burden	Neg	Neg											Neg						
1657	failure	1+	Pos	S										NTM						
1658	failure	Neg	I											Neg						
1659	high-burden	Neg	Neg											Pos	S	S				
1660	high-burden	Neg	Pos	S										Pos	S	S				
1661	high-burden	S	Neg											Pos	S	S				
1662	relapse	S	Pos	S										Neg						
1663	high-burden	Neg	Neg											Pos	S	S				
1664	relapse	3+	Pos	S										Pos	S	S				
1665	high-burden	1+	Pos	S										Pos	R	S				
1666	high-burden	Neg	Neg											Neg						
1667	high-burden	Neg	Neg											Neg						
1668	relapse	3+	Pos	S										Pos	S	S				
1669	high-burden	1+	Pos	S										Pos	S	S				
1670	high-burden	Neg	Neg											Pos	R	S				

Pt#	Group	AFS	Xpert			DR*plus* (WT absent, MUT binding)		DR*sl* (WT absent, MUT binding)		Sequencing (Discordant)				Convetional results						
			MTBC	RIF	Probe Failure	RIF	INH	FLQ	SLID	RIF	INH	FLQ	SLID	Cul.	INH	RIF	FLQ	KAN	AMK	CAP
1671	failure	Neg	Neg											Neg						
1672	high-burden	2+	Pos	S										Pos	S	S				
1673	high-burden	2+	Neg											Neg						
1674	high-risk	Neg	Neg											Neg						
1675	failure	1+	Pos	S										NTM						
1676	failure	1+	Neg											NTM						
1677	high-burden	Neg	Neg											Neg						
1678	failure	1+	Pos	S										Neg						
1679	high-risk	Neg	Pos	S										Pos	S	S				
1680	default	2+	Pos	S										Pos	S	S				
1681	high-risk	Neg	Neg											Neg						
1682	high-burden	3+	Pos	S										Pos	S	S				
1683	high-risk	1+	Neg											Neg						
1684	failure	1+	Neg											Neg						
1685	failure	1+	Neg											Neg						
1686	failure	-	Pos	S										Neg						
1687	relapse	S	Neg											Neg						
1688	relapse	Neg	Pos	S										Pos	S	S				
1689	high-risk	Neg	Pos	S										Pos	S	S				
1690	relapse	Neg	Neg											Neg						
1691	high-burden	Neg	Neg											Neg						
1692	high-burden	Neg	Neg											Pos	S	S				
1693	high-risk	Neg	Neg											Missing						
1694	default	Neg	Neg											Neg						
1695	relapse	Neg	Pos	S										Pos	S	S				
1696	relapse	S	Neg											NTM						
1697	high-risk	1+	Neg											Pos	S	S				
1698	relapse	Neg	Neg											Pos	S	S				

Pt#	Group	AFS	Xpert			DR*plus* (WT absent, MUT binding)		DR*sl* (WT absent, MUT binding)		Sequencing (Discordant)				Convetional results						
			MTBC	RIF	Probe Failure	RIF	INH	FLQ	SLID	RIF	INH	FLQ	SLID	Cul.	INH	RIF	FLQ	KAN	AMK	CAP
1699	high-burden	4+	Pos	S										Pos	S	S	S	S	-	S
1700	high-burden	Neg	Neg											Neg						
1701	relapse	Neg	Neg											Neg						
1702	relapse	1+	Neg											NTM						
1703	relapse	1+	Neg											NTM						
1704	high-burden	4+	Neg											Neg						
1705	failure	2+	Pos	S										Pos	S	S				
1706	relapse	Neg	Neg											NTM						
1707	high-burden	Neg	Neg											Neg						
1708	relapse	Neg	Neg											Pos	S	S				
1709	relapse	Neg	Neg											Neg						
1710	relapse	Neg	Neg											Neg						
1711	failure	Neg	Neg											Neg						
1712	high-risk	Neg	Neg											Neg						
1713	relapse	1+	Pos	S										Neg						
1714	relapse	1+	Pos	S										Neg						
1715	relapse	2+	Pos	S										Pos	S	S				
1716	high-burden	1+	Pos	S										Pos	S	S				
1717	failure	3+	Pos	S										Pos	S	S				
1718	relapse	1+	Pos	S										Neg						
1719	failure	1+	Pos	S										NTM						
1720	high-burden	Neg	Neg											Pos	S	S				
1721	relapse	1+	Neg											NTM						
1722	failure	1+	Pos	S										Pos	R	S				
1723	failure	2+	Pos	S										Pos	S	S				
1724	high-burden	1+	Pos	S										Pos	S	S				
1725	high-risk	1+	Neg											Pos	S	S				
1726	high-burden	4+	Pos	S										Pos	S	S				

Pt#	Group	AFS	Xpert			DR*plus* (WT absent, MUT binding)		DR*sl* (WT absent, MUT binding)		Sequencing (Discordant)				Convetional results						
			MTBC	RIF	Probe Failure	RIF	INH	FLQ	SLID	RIF	INH	FLQ	SLID	Cul.	INH	RIF	FLQ	KAN	AMK	CAP
1727	high-burden	S	Pos	S										Neg						
1728	high-burden	Neg	Pos	S										Neg						
1729	failure	2+	Pos	S										Neg						
1730	failure	1+	Neg											Neg						
1731	relapse	Neg	Pos	S										NTM						
1732	high-burden	Neg	Neg											Pos	S	S				
1733	high-burden	Neg	Neg											Neg						
1734	high-burden	Neg	Neg											Neg						
1735	high-burden	Neg	Neg											Neg						
1736	high-burden	Neg	Neg											Pos	S	S				
1737	relapse	Neg	Neg											Neg						
1738	high-burden	Neg	Neg											Neg						
1739	relapse	2+	Pos	R	E	WT8,MUT3	*inhA* WT2,MUT3A							Pos	R	R	S	S	S	S
1740	relapse	3+	Pos	S										Pos	S	S				
1741	high-burden	4+	Pos	S										Pos	S	S				
1742	relapse	S	Neg											NTM						
1743	failure	1+	Pos	S										Pos	S	S				
1744	failure	2+	Pos	S										Neg						
1745	relapse	1+	Pos	S										Pos	S	S				
1746	failure	Neg	Neg											Neg						
1747	failure	S	Neg											NTM						
1748	relapse	Neg	Neg											Pos	S	S				
1749	relapse	Neg	Neg											Neg						
1750	high-burden	-	Pos	S										Pos	S	S				
1751	high-burden	3+	Pos	S										Pos	S	S				
1752	high-burden	Neg	Neg											Neg						
1753	high-burden	Neg	Neg											Neg						
1754	relapse	Neg	Neg											NTM						

Pt#	Group	AFS	Xpert			DR*plus* (WT absent, MUT binding)		DR*sl* (WT absent, MUT binding)		Sequencing (Discordant)				Convetional results						
			MTBC	RIF	Probe Failure	RIF	INH	FLQ	SLID	RIF	INH	FLQ	SLID	Cul.	INH	RIF	FLQ	KAN	AMK	CAP
1755	high-burden	Neg	Neg											Neg						
1756	high-burden	Neg	Neg											Neg						
1757	high-burden	Neg	Neg											Neg						
1758	failure	1+	Pos	S										Pos	S	S				
1759	high-burden	Neg	Neg											Pos	S	S				
1760	high-burden	Neg	Neg											Pos	S	S				
1761	high-burden	Neg	Neg											Neg						
1762	high-burden	1+	Pos	S										Pos	S	S				
1763	relapse	2+	Neg											Pos	R	S				
1764	high-burden	Neg	Neg											NTM						
1765	high-burden	Neg	Neg											Neg						
1766	relapse	Neg	Neg											Neg						
1767	high-burden	1+	Pos	S										Pos	S	S				
1768	high-risk	1+	Pos	S										Pos	S	S				
1769	relapse	2+	Neg											NTM						
1770	default	1+	Pos	S										Pos	S	S				
1771	relapse	S	Neg											NTM						
1772	failure	1+	Neg											Neg						
1773	high-burden	Neg	Pos	S										Pos	S	S				
1774	high-burden	Neg	Neg											Neg						
1775	high-burden	Neg	Neg											Neg						
1776	high-burden	Neg	Neg											Neg						
1777	relapse	4+	Pos	R	A	WT2	S			L511P				Pos	S	S	S	S	-	S
1778	failure	1+	Neg											Neg						
1779	default	4+	Pos	S										Pos	S	S				
1780	relapse	Neg	Neg											Neg						
1781	relapse	Neg	Neg											Neg						
1782	high-burden	4+	Pos	S										Pos	S	S				

Pt#	Group	AFS	Xpert			DR*plus* (WT absent, MUT binding)		DR*sl* (WT absent, MUT binding)		Sequencing (Discordant)				Convetional results						
			MTBC	RIF	Probe Failure	RIF	INH	FLQ	SLID	RIF	INH	FLQ	SLID	Cul.	INH	RIF	FLQ	KAN	AMK	CAP
1783	close contacts	S	Pos	S										Pos	S	S				
1784	high-burden	Neg	Neg											Neg						
1785	failure	Neg	Neg											Neg						
1786	relapse	4+	Neg											NTM						
1787	high-burden	Neg	Neg											Neg						
1788	relapse	Neg	Neg											Neg						
1789	relapse	4+	Pos	S										Pos	S	S				
1790	relapse	1+	Pos	S										Pos	S	S				
1791	failure	Neg	Neg											Pos	S	S				
1792	relapse	1+	Neg											NTM						
1793	close contacts	1+	Neg											Neg						
1794	high-burden	3+	Neg											NTM						
1795	high-burden	Neg	Neg											Neg						
1796	high-risk	Neg	Neg											Neg						
1797	relapse	1+	Neg											Neg						
1798	high-burden	Neg	Neg											Pos	S	S				
1799	high-burden	Neg	Neg											Neg						
1800	high-burden	1+	Pos	R	D	WT7	*katG* WT,MUT1		*rrs* WT1,WT2, MUT2					Pos	R	R	S	R	R	R
1801	relapse	4+	Pos	S										Pos	R	S				
1802	relapse	3+	Neg											NTM						
1803	relapse	1+	Neg											NTM						
1804	failure	-	Neg											NTM						
1805	high-burden	S	Neg											Neg						
1806	high-burden	Neg	Neg											Neg						
1807	close contacts	S	Pos	S										Pos	S	S				
1808	relapse	1+	Pos	S										Neg						
1809	high-burden	Neg	Neg											Pos	S	S				

Pt#	Group	AFS	Xpert			DR*plus* (WT absent, MUT binding)		DR*sl* (WT absent, MUT binding)		Sequencing (Discordant)				Convetional results						
			MTBC	RIF	Probe Failure	RIF	INH	FLQ	SLID	RIF	INH	FLQ	SLID	Cul.	INH	RIF	FLQ	KAN	AMK	CAP
1810	relapse	-	Pos	S										Neg						
1811	relapse	2+	Pos	R	E	WT8,MUT3	*katG* WT,MUT1							Pos	R	R	S	S	S	S
1812	relapse	Neg	Neg											Pos	S	S				
1813	relapse	1+	Pos	S										Pos	R	S				
1814	relapse	Neg	Neg											Neg						
1815	high-risk	S	Neg											Pos	S	S				
1816	failure	1+	Pos	S										Neg						
1817	relapse	1+	Neg											NTM						
1818	high-risk	4+	Pos	S										Pos	S	S				
1819	high-burden	1+	Pos	S										Pos	S	S				
1820	high-burden	Neg	Neg											Pos	S	S				
1821	default	4+	Pos	S										Pos	S	S				
1822	high-burden	Neg	Neg											Pos	S	S				
1823	high-burden	Neg	Neg											Neg						
1824	relapse	Neg	Neg											Neg						
1825	failure	Neg	Neg											Neg						
1826	relapse	Neg	Neg											Neg						
1827	relapse	2+	Neg											NTM						
1828	high-risk	2+	Pos	S										Pos	S	S				
1829	high-burden	1+	Neg											NTM						
1830	failure	2+	Pos	S										Neg						
1831	high-burden	Neg	Neg											Pos	S	S				
1832	high-burden	Neg	Neg											Neg						
1833	high-burden	Neg	Neg											Neg						
1834	high-burden	Neg	Neg											Neg						
1835	high-burden	Neg	Neg											Neg						
1836	high-burden	-	Pos	I										Pos	S	S				
1837	high-burden	Neg	Neg											Neg						

Pt#	Group	AFS	Xpert			DR*plus* (WT absent, MUT binding)		DR*sl* (WT absent, MUT binding)		Sequencing (Discordant)				Convetional results						
			MTBC	RIF	Probe Failure	RIF	INH	FLQ	SLID	RIF	INH	FLQ	SLID	Cul.	INH	RIF	FLQ	KAN	AMK	CAP
1838	high-burden	3+	Pos	S										Pos	S	S				
1839	high-burden	3+	Neg											Pos	S	S				
1840	close contacts	3+	Pos	S										Pos	S	S	S	S	-	S
1841	relapse	Neg	Neg											Neg						
1842	failure	S	Pos	S										Pos	S	S				
1843	relapse	1+	Pos	S										Neg						
1844	high-burden	Neg	Neg											Neg						
1845	high-burden	Neg	Neg											Neg						
1846	default	Neg	Neg											Neg						
1847	default	Neg	Neg											Neg						
1848	relapse	Neg	Neg											Neg						
1849	relapse	Neg	Neg											Missing						
1850	failure	1+	Pos	S										Pos	S	S				
1851	relapse	Neg	Pos	S										Neg						
1852	failure	1+	Pos	S										Neg						
1853	relapse	-	Neg											NTM						
1854	relapse	1+	Neg											Neg						
1855	relapse	Neg	Neg											Neg						
1856	relapse	2+	Pos	R	E	WT8,MUT3	*katG* WT,MUT1		*rrs* WT1,MUT 1					Pos	R	R	S	R	R	R
1857	relapse	1+	Pos	S										Pos	S	S				
1858	relapse	1+	Neg											NTM						
1859	high-burden	1+	Pos	S										Pos	S	S				
1860	high-burden	Neg	Neg											Neg						
1861	high-burden	Neg	Neg											Neg						
1862	failure	1+	Neg											Pos	S	S				
1863	high-burden	Neg	Neg											Neg						
1864	relapse	-	Neg											Neg						

Pt#	Group	AFS	Xpert			DR*plus* (WT absent, MUT binding)		DR*sl* (WT absent, MUT binding)		Sequencing (Discordant)				Convetional results						
			MTBC	RIF	Probe Failure	RIF	INH	FLQ	SLID	RIF	INH	FLQ	SLID	Cul.	INH	RIF	FLQ	KAN	AMK	CAP
1865	high-burden	Neg	Neg											Pos	S	S				
1866	relapse	1+	Pos	R	E	WT8,MUT3	*inhA* WT1,MUT1							Pos	R	R	S	S	S	S
1867	relapse	1+	Pos	S										Pos	S	S				
1868	relapse	2+	Neg											NTM						
1869	high-risk	Neg	Pos	R	E	WT8,MUT3	*katG* WT,MUT1<br>*inhA* WT1,MUT1							Pos	R	R	S	S	S	S
1870	high-burden	Neg	Neg											Neg						
1871	high-burden	Neg	Neg											Neg						
1872	failure	-	Pos	S										NTM						
1873	high-burden	Neg	Pos	S										Pos	R	S				
1874	failure	Neg	Neg											Neg						
1875	failure	1+	Pos	S										Pos	S	S				
1876	high-burden	Neg	Pos	S										Pos	S	S				
1877	relapse	3+	Pos	S										Pos	S	S				
1878	relapse	S	Neg											NTM						
1879	high-burden	Neg	Pos	S										Pos	S	S				
1880	relapse	Neg	Neg											Neg						
1881	high-burden	Neg	Neg											Neg						
1882	high-burden	Neg	Neg											Neg						
1883	default	3+	Pos	S										Pos	S	S				
1884	failure	Neg	Neg											Neg						
1885	failure	Neg	Neg											Neg						
1886	failure	Neg	Neg											Neg						
1887	high-burden	Neg	Neg											Neg						
1888	failure	1+	Pos	S										NTM						
1889	high-burden	Neg	Neg											Pos	R	S				
1890	relapse	S	Pos	S										Neg						
1891	high-burden	Neg	Neg											Neg						

Pt#	Group	AFS	Xpert			DR*plus* (WT absent, MUT binding)		DR*sl* (WT absent, MUT binding)		Sequencing (Discordant)				Convetional results						
			MTBC	RIF	Probe Failure	RIF	INH	FLQ	SLID	RIF	INH	FLQ	SLID	Cul.	INH	RIF	FLQ	KAN	AMK	CAP
1892	high-burden	Neg	Neg											Pos	R	S				
1893	relapse	1+	Pos	S										Pos	S	S				
1894	high-burden	Neg	Neg											Neg						
1895	relapse	-	Neg											Pos	S	S				
1896	high-burden	Neg	Neg											Pos	S	S				
1897	relapse	Neg	Neg											Neg						
1898	failure	1+	Neg											Neg						
1899	high-burden	Neg	Pos	S										Pos	S	S				
1900	relapse	3+	Pos	S										Pos	S	S				
1901	failure	1+	Pos	S										NTM						
1902	relapse	1+	Pos	S										Pos	S	S				
1903	failure	S	Pos	I										Neg						
1904	relapse	S	Neg											NTM						
1905	relapse	Neg	Neg											Pos	S	S				
1906	high-burden	Neg	Neg											Neg						
1907	relapse	S	Neg											Neg						
1908	high-burden	Neg	Neg											Neg						
1909	high-burden	Neg	Neg											Pos	S	S				
1910	failure	2+	Neg											NTM						
1911	high-burden	Neg	Neg											Neg						
1912	high-burden	Neg	Neg											Neg						
1913	high-burden	2+	Pos	S										Pos	S	S				
1914	relapse	1+	Pos	R	D	WT7	*katG* WT,MUT1							Pos	R	R	S	S	S	S
1915	relapse	Neg	Neg											Neg						
1916	relapse	Neg	Neg											Neg						
1917	relapse	Neg	Neg											Neg						
1918	relapse	Neg	Neg											Neg						
1919	relapse	1+	Neg											NTM						

Pt#	Group	AFS	Xpert			DR*plus* (WT absent, MUT binding)		DR*sl* (WT absent, MUT binding)		Sequencing (Discordant)				Convetional results						
			MTBC	RIF	Probe Failure	RIF	INH	FLQ	SLID	RIF	INH	FLQ	SLID	Cul.	INH	RIF	FLQ	KAN	AMK	CAP
1920	relapse	Neg	Neg											Neg						
1921	default	Neg	Neg											Neg						
1922	failure	Neg	Pos	R	D	WT7,MUT2A	*katG* WT,MUT1							Pos	R	R	S	S	S	S
1923	relapse	Neg	Neg											Neg						
1924	high-burden	3+	Pos	S										Pos	S	S				
1925	relapse	3+	Neg											Neg						
1926	failure	1+	Pos	S										Pos	S	S				
1927	default	Neg	Neg											Neg						
1928	relapse	Neg	Neg											Neg						
1929	high-burden	S	Neg											Neg						
1930	relapse	S	Pos	R	E	WT8	*katG* WT,MUT1			L533P				Pos	R	S	S	S	-	S
1931	high-burden	2+	Pos	S										Pos	S	S				
1932	relapse	4+	Pos	S										Pos	S	S				
1933	high-burden	Neg	Pos	S										Pos	R	S				
1934	relapse	Neg	Neg											Neg						
1935	high-burden	S	Neg											Neg						
1936	relapse	2+	Pos	S										Pos	S	S				
1937	high-risk	-	Neg											Pos	S	S				
1938	high-risk	Neg	Neg											Neg						
1939	high-burden	Neg	Neg											Pos	S	S				
1940	high-burden	Neg	Neg											Pos	S	S				
1941	relapse	-	Neg											NTM						
1942	high-burden	Neg	Neg											Pos	S	S				
1943	relapse	Neg	Pos	S										Pos	S	S				
1944	high-burden	2+	Pos	S										Pos	S	S				
1945	relapse	4+	Pos	S										Pos	S	S				
1946	relapse	-	Neg											NTM						
1947	high-burden	Neg	Neg											Neg						

Pt#	Group	AFS	Xpert			DR*plus* (WT absent, MUT binding)		DR*sl* (WT absent, MUT binding)		Sequencing (Discordant)				Convetional results						
			MTBC	RIF	Probe Failure	RIF	INH	FLQ	SLID	RIF	INH	FLQ	SLID	Cul.	INH	RIF	FLQ	KAN	AMK	CAP
1948	relapse	Neg	Neg											Neg						
1949	relapse	Neg	Neg											Neg						
1950	high-burden	S	Pos	S										Pos	S	S				
1951	high-burden	Neg	Neg											Pos	S	S				
1952	high-burden	Neg	Neg											NTM						
1953	relapse	Neg	Neg											Pos	S	S				
1954	high-burden	Neg	Neg											Neg						
1955	high-burden	Neg	Pos	S										Pos	S	S				
1956	relapse	3+	Neg											NTM						
1957	failure	Neg	Neg											Neg						
1958	failure	Neg	Neg											Neg						
1959	high-burden	Neg	Neg											Neg						
1960	failure	1+	Pos	S										Neg						
1961	failure	1+	Neg											Neg						
1962	high-burden	Neg	Neg											Pos	S	S				
1963	failure	Neg	Neg											Neg						
1964	high-risk	3+	Pos	S										Pos	S	S				
1965	high-burden	2+	Neg											Neg						
1966	failure	Neg	Neg											Neg						
1967	high-burden	Neg	Neg											Neg						
1968	high-burden	4+	Pos	S										Pos	S	S				
1969	relapse	1+	Neg											NTM						
1970	relapse	2+	Pos	S										Pos	S	S				
1971	relapse	3+	Neg											NTM						
1972	high-burden	Neg	Neg											Neg						
1973	high-burden	Neg	Neg											Pos	S	S				
1974	relapse	Neg	Neg											Missing						
1975	high-burden	Neg	Neg											Pos	S	S				

Pt#	Group	AFS	Xpert			DR*plus* (WT absent, MUT binding)		DR*sl* (WT absent, MUT binding)		Sequencing (Discordant)				Convetional results						
			MTBC	RIF	Probe Failure	RIF	INH	FLQ	SLID	RIF	INH	FLQ	SLID	Cul.	INH	RIF	FLQ	KAN	AMK	CAP
1976	high-burden	Neg	Neg											Neg						
1977	high-burden	Neg	Neg											Neg						
1978	relapse	Neg	Pos	S										Pos	S	S				
1979	relapse	1+	Neg											NTM						
1980	relapse	Neg	Neg											Pos	S	S	S	S	-	S
1981	high-burden	4+	Pos	S										Pos	S	S				
1982	high-burden	Neg	Neg											Pos	S	S				
1983	failure	Neg	Neg											Neg						
1984	failure	Neg	Neg											Neg						
1985	failure	Neg	Neg											Neg						
1986	high-burden	Neg	Neg											Neg						
1987	high-burden	Neg	Neg											Neg						
1988	high-risk	3+	Pos	S										Pos	S	S				
1989	relapse	2+	Neg											Neg						
1990	relapse	Neg	Neg											Neg						
1991	high-risk	S	Pos	S										Pos	S	S				
1992	relapse	2+	Pos	R	B	WT3,WT4	S							Pos	S	R	S	S	-	S
1993	relapse	1+	Neg											Neg						
1994	relapse	1+	Neg											NTM						
1995	relapse	2+	Pos	S										Pos	S	S				
1996	default	Neg	Neg											Neg						
1997	default	Neg	Neg											Neg						
1998	relapse	Neg	Neg											Neg						
1999	high-burden	Neg	Neg											Neg						
2000	high-burden	Neg	Neg											Neg						
2001	close contacts	Neg	Neg											Neg						
2002	high-burden	Neg	Neg											Neg						
2003	high-burden	Neg	Neg											Pos	S	S				

Pt#	Group	AFS	Xpert			DR*plus* (WT absent, MUT binding)		DR*sl* (WT absent, MUT binding)		Sequencing (Discordant)				Convetional results						
			MTBC	RIF	Probe Failure	RIF	INH	FLQ	SLID	RIF	INH	FLQ	SLID	Cul.	INH	RIF	FLQ	KAN	AMK	CAP
2004	high-burden	Neg	Neg											Neg						
2005	relapse	1+	Pos	S										Pos	S	S				
2006	failure	3+	Pos	S										Pos	R	S				
2007	relapse	-	Pos	R	D	WT7	S							Pos	S	R	S	S	-	S
2008	relapse	3+	Pos	S										Pos	S	S				
2009	unknown	Neg	Neg											Neg						
2010	relapse	2+	Pos	S										Pos	S	S	S	S	-	S
2011	relapse	Neg	Neg											Neg						
2012	failure	Neg	Neg											Neg						
2013	relapse	S	Neg											Neg						
2014	relapse	3+	Pos	S										Pos	R	S				
2015	relapse	Neg	Neg											Neg						
2016	relapse	Neg	Neg											Neg						
2017	high-burden	Neg	Neg											Pos	S	S				
2018	high-burden	Neg	Neg											Neg						
2019	high-burden	Neg	Neg											Pos	S	S				
2020	high-burden	Neg	Neg											Pos	S	S				
2021	relapse	3+	Pos	S										Pos	S	S				
2022	high-burden	Neg	Pos	S										Pos	S	S				
2023	high-burden	Neg	Neg											Neg						
2024	high-burden	Neg	Neg											Neg						
2025	failure	1+	Neg											Neg						
2026	high-burden	Neg	Neg											Neg						
2027	failure	1+	Neg											NTM						
2028	high-risk	Neg	Pos	S										Pos	S	S				
2029	high-burden	Neg	Neg											Neg						
2030	high-burden	Neg	Neg											Neg						
2031	failure	1+	Pos	S										Pos	S	S				

Pt#	Group	AFS	Xpert			DR*plus* (WT absent, MUT binding)		DR*sl* (WT absent, MUT binding)		Sequencing (Discordant)				Convetional results						
			MTBC	RIF	Probe Failure	RIF	INH	FLQ	SLID	RIF	INH	FLQ	SLID	Cul.	INH	RIF	FLQ	KAN	AMK	CAP
2032	relapse	Neg	Neg											Neg						
2033	relapse	-	Pos	S										Pos	S	S				
2034	relapse	S	Pos	S										Neg						
2035	high-burden	Neg	Neg											Pos	S	S	S	S	-	S
2036	high-risk	1+	Pos	S										Pos	S	S				
2037	failure	1+	Neg											Neg						
2038	failure	3+	Pos	S										Pos	S	S				
2039	relapse	1+	Pos	S										Pos	S	S				
2040	relapse	1+	Neg											NTM						
2041	relapse	1+	Neg											Neg						
2042	failure	3+	Pos	S										Pos	S	S				
2043	relapse	2+	Pos	S										Pos	S	S				
2044	high-burden	Neg	Neg											Neg						
2045	high-burden	Neg	Neg											Neg						
2046	high-burden	Neg	Neg											Neg						
2047	high-risk	Neg	Pos	S										Pos	S	S				
2048	failure	S	Neg											Neg						
2049	unknown	-	Pos	S										Neg						
2050	failure	1+	Pos	S										Pos	S	S	S	S	-	S
2051	unknown	Neg	Neg											Neg						
2052	relapse	1+	Pos	S										Neg						
2053	unknown	2+	Pos	S										Pos	S	S				
2054	relapse	Neg	Neg											Neg						
2055	relapse	Neg	Neg											Neg						
2056	relapse	3+	Pos	S										Pos	S	S				
2057	high-burden	Neg	Neg											Pos	S	S				
2058	relapse	Neg	Neg											Neg						
2059	failure	1+	Pos	S										Neg						

Pt#	Group	AFS	Xpert			DR*plus* (WT absent, MUT binding)		DR*sl* (WT absent, MUT binding)		Sequencing (Discordant)				Convetional results						
			MTBC	RIF	Probe Failure	RIF	INH	FLQ	SLID	RIF	INH	FLQ	SLID	Cul.	INH	RIF	FLQ	KAN	AMK	CAP
2060	failure	1+	Neg											NTM						
2061	unknown	1+	Neg											Neg						
2062	relapse	Neg	Neg											Missing						
2063	relapse	Neg	Neg											Neg						
2064	relapse	3+	Pos	S										Pos	R	S				
2065	high-burden	3+	Pos	S										Pos	S	S				
2066	high-burden	Neg	Neg											Neg						
2067	high-burden	Neg	Neg											Neg						
2068	unknown	-	Neg											Pos	S	S				
2069	high-burden	-	Neg											NTM						
2070	high-burden	Neg	Neg											Neg						
2071	failure	Neg	Pos	S										Pos	S	S				
2072	relapse	S	Pos	S										Neg						
2073	failure	1+	Pos	S										Neg						
2074	unknown	Neg	Neg											Pos	S	S				
2075	high-burden	Neg	Neg											Pos	S	S				
2076	relapse	1+	Pos	R	E	WT8,MUT3	*katG* WT,MUT1							Pos	R	R	S	S	S	S
2077	high-burden	1+	Neg											NTM						
2078	relapse	1+	Neg											Neg						
2079	high-burden	2+	Neg											NTM						
2080	high-burden	Neg	Neg											Pos	S	S				
2081	relapse	3+	Pos	S										NTM						
2082	relapse	2+	Neg											NTM						
2083	failure	Neg	Neg											Pos	R	S	S	S	S	S
2084	high-burden	Neg	Neg											Neg						
2085	high-burden	Neg	Neg											Neg						
2086	high-burden	-	Neg											NTM						
2087	high-burden	2+	Pos	S										Pos	S	S				

Pt#	Group	AFS	Xpert			DR*plus* (WT absent, MUT binding)		DR*sl* (WT absent, MUT binding)		Sequencing (Discordant)				Convetional results						
			MTBC	RIF	Probe Failure	RIF	INH	FLQ	SLID	RIF	INH	FLQ	SLID	Cul.	INH	RIF	FLQ	KAN	AMK	CAP
2088	high-burden	Neg	Neg											Neg						
2089	relapse	2+	Neg											Neg						
2090	relapse	1+	Neg											NTM						
2091	unknown	-	Neg											Neg						
2092	high-burden	Neg	Neg											Neg						
2093	high-burden	1+	Pos	S										Pos	S	S				
2094	relapse	Neg	Neg											Neg						
2095	high-burden	Neg	Neg											Pos	S	S				
2096	relapse	1+	Neg											NTM						
2097	unknown	1+	Pos	S										Pos	S	S				
2098	unknown	1+	Neg											Neg						
2099	unknown	Neg	Neg											Pos	S	S				
2100	high-burden	4+	Pos	S										Pos	S	S				
2101	high-burden	Neg	Neg											Pos	R	R	S	S	S	S
2102	relapse	Neg	Neg											Pos	S	S				
2103	high-burden	Neg	Neg											Neg						
2104	unknown	Neg	Neg											Neg						
2105	high-burden	Neg	Neg											Pos	S	S				
2106	unknown	Neg	Neg											Neg						
2107	high-risk	Neg	Neg											Neg						
2108	high-burden	2+	Pos	S										Pos	S	S				
2109	high-burden	Neg	Neg											Neg						
2110	high-burden	Neg	Neg											Pos	S	S				
2111	relapse	1+	Pos	S										Pos	S	S				
2112	failure	1+	Pos	S										Pos	R	S				
2113	high-burden	Neg	Neg											Pos	S	S				
2114	high-burden	3+	Pos	S										Pos	S	S				
2115	relapse	1+	Pos	S										Pos	S	S				

Pt#	Group	AFS	Xpert			DR*plus* (WT absent, MUT binding)		DR*sl* (WT absent, MUT binding)		Sequencing (Discordant)				Convetional results						
			MTBC	RIF	Probe Failure	RIF	INH	FLQ	SLID	RIF	INH	FLQ	SLID	Cul.	INH	RIF	FLQ	KAN	AMK	CAP
2116	relapse	1+	Neg											NTM						
2117	high-burden	Neg	Neg											Pos	S	S				
2118	high-risk	2+	Pos	S										Pos	S	S				
2119	high-burden	Neg	Neg											Neg						
2120	high-burden	1+	Pos	S										Pos	R	S				
2121	failure	1+	Pos	S										Neg						
2122	high-burden	Neg	Neg											Pos	S	S				
2123	high-burden	Neg	Neg											Pos	S	S				
2124	high-burden	Neg	Neg											Neg						
2125	relapse	4+	Pos	S										Pos	S	S				
2126	relapse	Neg	Pos	S										Pos	R	S				
2127	high-burden	Neg	I											Pos	S	S				
2128	relapse	Neg	Neg											Neg						
2129	unknown	Neg	Neg											Neg						
2130	high-burden	Neg	Neg											Pos	S	S				
2131	unknown	Neg	Neg											Neg						
2132	high-burden	1+	Pos	S										Pos	S	S				
2133	failure	1+	Neg											Neg						
2134	relapse	Neg	Neg											Neg						
2135	unknown	Neg	Neg											Neg						
2136	high-burden	Neg	Neg											Pos	S	S				
2137	high-burden	3+	Pos	S										Pos	R	S				
2138	high-burden	Neg	Neg											Neg						
2139	relapse	2+	Neg											NTM						
2140	relapse	2+	Pos	S										Pos	S	S				
2141	high-burden	2+	Pos	S										Pos	S	S				
2142	high-burden	Neg	Neg											Neg						
2143	high-burden	Neg	Neg											NTM						

Pt#	Group	AFS	Xpert			DR*plus* (WT absent, MUT binding)		DR*sl* (WT absent, MUT binding)		Sequencing (Discordant)				Convetional results						
			MTBC	RIF	Probe Failure	RIF	INH	FLQ	SLID	RIF	INH	FLQ	SLID	Cul.	INH	RIF	FLQ	KAN	AMK	CAP
2144	relapse	Neg	Pos	S										Pos	S	S				
2145	relapse	Neg	Neg											Neg						
2146	high-burden	Neg	Neg											Neg						
2147	high-burden	Neg	Neg											Neg						
2148	unknown	2+	Pos	S										Pos	R	S				
2149	high-burden	Neg	Neg											Neg						
2150	high-burden	Neg	Neg											Neg						
2151	unknown	Neg	Neg											Neg						
2152	high-burden	Neg	Neg											Neg						
2153	high-burden	Neg	Neg											Neg						
2154	high-burden	Neg	Neg											Pos	S	S				
2155	relapse	2+	Neg											NTM						
2156	failure	1+	Neg											Neg						
2157	relapse	1+	Neg											Neg						
2158	relapse	Neg	Neg											Neg						
2159	relapse	1+	Neg											Neg						
2160	unknown	Neg	Neg											Neg						
2161	high-burden	Neg	Neg											Neg						
2162	high-burden	Neg	Neg											Pos	S	S				
2163	relapse	2+	Pos	S										Pos	S	S				
2164	high-burden	Neg	Neg											Neg						
2165	relapse	1+	Pos	S										Pos	S	S				
2166	relapse	4+	Neg											Neg						
2167	high-burden	1+	Pos	S										Pos	S	S				
2168	high-burden	Neg	Neg											Neg						
2169	unknown	2+	Pos	S										Pos	S	S				
2170	unknown	Neg	Neg											Pos	S	S				
2171	high-burden	Neg	Neg											Neg						

Pt#	Group	AFS	Xpert			DR*plus* (WT absent, MUT binding)		DR*sl* (WT absent, MUT binding)		Sequencing (Discordant)				Convetional results						
			MTBC	RIF	Probe Failure	RIF	INH	FLQ	SLID	RIF	INH	FLQ	SLID	Cul.	INH	RIF	FLQ	KAN	AMK	CAP
2172	failure	1+	Neg											NTM						
2173	high-burden	Neg	Neg											Neg						
2174	high-burden	4+	Pos	S										Pos	S	S				
2175	high-burden	Neg	Neg											Neg						
2176	high-burden	3+	Pos	S										Pos	S	S				
2177	high-burden	Neg	Neg											Neg						
2178	close contacts	1+	Neg											NTM						
2179	high-burden	Neg	Neg											Pos	S	S				
2180	high-burden	Neg	Neg											Neg						
2181	high-burden	Neg	Neg											Neg						
2182	high-burden	S	Neg											Neg						
2183	failure	2+	Pos	S										Neg						
2184	unknown	Neg	Neg											Neg						
2185	relapse	1+	Pos	S										Pos	S	S				
2186	unknown	1+	Neg											NTM						
2187	relapse	Neg	Neg											Neg						
2188	relapse	Neg	Neg											Pos	S	S				
2189	relapse	-	Pos	S										Pos	S	S				
2190	relapse	-	Neg											Pos	S	S				
2191	unknown	S	Pos	S										Pos	S	S				
2192	relapse	1+	Pos	S										Pos	S	S				
2193	relapse	Neg	Neg											Neg						
2194	relapse	Neg	Neg											Pos	S	S	S	S	-	S
2195	high-burden	Neg	Neg											Neg						
2196	high-burden	Neg	Neg											Neg						
2197	high-burden	Neg	Neg											Neg						
2198	high-burden	Neg	Neg											Pos	S	S				
2199	high-burden	Neg	Pos	S										Neg						

Pt#	Group	AFS	Xpert			DR*plus* (WT absent, MUT binding)		DR*sl* (WT absent, MUT binding)		Sequencing (Discordant)				Convetional results						
			MTBC	RIF	Probe Failure	RIF	INH	FLQ	SLID	RIF	INH	FLQ	SLID	Cul.	INH	RIF	FLQ	KAN	AMK	CAP
2200	failure	1+	Pos	S										Neg						
2201	high-burden	1+	Pos	S										Pos	S	S				
2202	high-burden	1+	Pos	S										Pos	S	S				
2203	high-burden	Neg	Neg											Pos	S	S				
2204	high-burden	Neg	Neg											Neg						
2205	unknown	Neg	Neg											Neg						
2206	relapse	1+	Pos	S										Pos	S	S				
2207	high-burden	Neg	Neg											Pos	S	S				
2208	relapse	3+	Pos	S										Pos	S	S				
2209	high-burden	Neg	Neg											Neg						
2210	high-burden	Neg	Neg											Pos	S	S	S	S	-	S
2211	high-risk	Neg	Neg											Neg						
2212	relapse	Neg	Neg											Neg						
2213	default	4+	Pos	S										Pos	R	S				
2214	relapse	2+	Neg											NTM						
2215	relapse	1+	Pos	S										Pos	S	S				
2216	relapse	2+	Pos	S										NTM						
2217	high-risk	Neg	Neg											Neg						
2218	unknown	Neg	Neg											Neg						
2219	failure	Neg	Neg											Neg						
2220	relapse	Neg	Neg											Neg						
2221	failure	Neg	Pos	S										Neg						
2222	relapse	-	Neg											NTM						
2223	relapse	4+	Neg											NTM						
2224	failure	1+	Pos	S										Pos	S	S				
2225	high-burden	Neg	Neg											Neg						
2226	relapse	Neg	Neg											Neg						
2227	high-burden	Neg	Neg											NTM						

Pt#	Group	AFS	Xpert			DR*plus* (WT absent, MUT binding)		DR*sl* (WT absent, MUT binding)		Sequencing (Discordant)				Convetional results						
			MTBC	RIF	Probe Failure	RIF	INH	FLQ	SLID	RIF	INH	FLQ	SLID	Cul.	INH	RIF	FLQ	KAN	AMK	CAP
2228	relapse	S	Neg											Neg						
2229	failure	1+	Pos	S										Neg						
2230	high-burden	4+	Pos	S										Pos	S	S				
2231	high-burden	1+	Pos	S										Pos	S	S				
2232	failure	-	Pos	S										NTM						
2233	high-burden	-	Neg											Pos	S	S				
2234	relapse	-	Pos	S										Pos	S	S				
2235	relapse	4+	Pos	S										Pos	S	S				
2236	unknown	Neg	Neg											Neg						
2237	relapse	Neg	Neg											Neg						
2238	relapse	Neg	Neg											NTM						
2239	high-burden	Neg	Pos	S										Pos	S	S				
2240	high-burden	S	Neg											Neg						
2241	relapse	2+	Neg											NTM						
2242	high-risk	Neg	Neg											NTM						
2243	high-risk	Neg	Neg											NTM						
2244	relapse	Neg	Neg											Neg						
2245	relapse	Neg	Neg											Neg						
2246	high-risk	Neg	Pos	I										Pos	S	S				
2247	high-burden	Neg	Neg											NTM						
2248	failure	2+	Pos	S										Pos	S	S				
2249	high-burden	Neg	Neg											Neg						
2250	failure	1+	Neg											Neg						
2251	unknown	1+	Pos	S										Neg						
2252	high-burden	Neg	Neg											Neg						
2253	high-burden	Neg	Neg											Neg						
2254	high-burden	Neg	Neg											Neg						
2255	relapse	1+	Pos	S										Pos	S	S	S	S	-	S

Pt#	Group	AFS	Xpert			DR*plus* (WT absent, MUT binding)		DR*sl* (WT absent, MUT binding)		Sequencing (Discordant)				Convetional results						
			MTBC	RIF	Probe Failure	RIF	INH	FLQ	SLID	RIF	INH	FLQ	SLID	Cul.	INH	RIF	FLQ	KAN	AMK	CAP
2256	relapse	1+	Pos	S										Pos	S	S				
2257	failure	S	Pos	S										Neg						
2258	failure	4+	Pos	S										Pos	S	S				
2259	failure	S	Neg											NTM						
2260	high-burden	Neg	Neg											Neg						
2261	relapse	Neg	Neg											Neg						
2262	relapse	Neg	Neg											Pos	S	S				
2263	high-burden	Neg	Neg											Neg						
2264	close contacts	2+	Pos	R	B	WT3,WT4	*katG* WT,MUT1							Pos	R	R	S	S	S	S
2265	relapse	4+	Pos	S										Pos	S	S				
2266	failure	1+	Pos	S										Neg						
2267	failure	1+	Pos	S										Neg						
2268	unknown	2+	Pos	S										Pos	R	S				
2269	unknown	-	Neg											Neg						
2270	relapse	Neg	Neg											Pos	S	S				
2271	failure	1+	Pos	S										Neg						
2272	high-burden	Neg	Neg											Neg						
2273	high-burden	Neg	Neg											Neg						
2274	failure	Neg	Neg											Neg						
2275	close contacts	Neg	Neg											Neg						
2276	relapse	Neg	Neg											NTM						
2277	failure	Neg	Neg											Neg						
2278	failure	Neg	Pos	S										Pos	S	S				
2279	high-burden	Neg	Neg											Neg						
2280	high-burden	Neg	Neg											Neg						
2281	unknown	Neg	Neg											Neg						
2282	failure	Neg	Neg											Neg						

Pt#	Group	AFS	Xpert			DR*plus* (WT absent, MUT binding)		DR*sl* (WT absent, MUT binding)		Sequencing (Discordant)				Convetional results						
			MTBC	RIF	Probe Failure	RIF	INH	FLQ	SLID	RIF	INH	FLQ	SLID	Cul.	INH	RIF	FLQ	KAN	AMK	CAP
2283	relapse	3+	Neg											NTM						
2284	relapse	1+	Neg											NTM						
2285	high-burden	Neg	Neg											Pos	S	S				
2286	failure	3+	Pos	S										Pos	S	S				
2287	relapse	S	Neg											NTM						
2288	relapse	Neg	Neg											Neg						
2289	relapse	Neg	Neg											Neg						
2290	failure	1+	Pos	S										Pos	S	S				
2291	failure	-	Pos	S										Neg						
2292	relapse	-	Neg											NTM						
2293	failure	2+	Pos	S										Pos	S	S				
2294	relapse	Neg	Neg											Neg						
2295	relapse	3+	Pos	S										Pos	S	S				
2296	high-burden	Neg	Pos	S										Pos	S	S				
2297	relapse	1+	Pos	S										Pos	S	S				
2298	failure	2+	Pos	S										Pos	R	S				
2299	high-burden	Neg	Neg											Pos	R	S				
2300	failure	1+	Pos	S										NTM						
2301	failure	1+	Pos	S										Neg						
2302	high-burden	1+	Pos	S										Pos	S	S				
2303	unknown	Neg	Neg											Neg						
2304	relapse	2+	Pos	S										Neg						
2305	high-risk	1+	Pos	S										Pos	R	S				
2306	relapse	Neg	Neg											Neg						
2307	high-burden	Neg	Neg											Neg						
2308	high-burden	Neg	Neg											Neg						
2309	unknown	Neg	Neg											Neg						
2310	unknown	Neg	Neg											Neg						

Pt#	Group	AFS	Xpert			DR*plus* (WT absent, MUT binding)		DR*sl* (WT absent, MUT binding)		Sequencing (Discordant)				Convetional results						
			MTBC	RIF	Probe Failure	RIF	INH	FLQ	SLID	RIF	INH	FLQ	SLID	Cul.	INH	RIF	FLQ	KAN	AMK	CAP
2311	failure	2+	Pos	S										Pos	R	S				
2312	relapse	1+	Pos	S										Pos	S	S				
2313	failure	1+	Neg											Neg						
2314	high-risk	3+	Pos	S										Pos	S	S				
2315	high-burden	Neg	Neg											Neg						
2316	high-risk	1+	Pos	S										Pos	S	S				
2317	failure	1+	Pos	S										Pos	S	S				
2318	failure	1+	Neg											NTM						
2319	relapse	Neg	Neg											Neg						
2320	failure	Neg	Neg											Pos	R	S	S	S	-	S
2321	relapse	1+	Neg											Neg						
2322	relapse	Neg	Neg											Neg						
2323	high-burden	Neg	Neg											Neg						
2324	high-burden	Neg	Neg											Neg						
2325	unknown	Neg	Neg											Neg						
2326	failure	Neg	Neg											Neg						
2327	high-burden	Neg	Neg											Neg						
2328	high-burden	Neg	Neg											Neg						
2329	high-burden	1+	Pos	S										Pos	S	S	S	S	-	S
2330	high-burden	Neg	Neg											Pos	S	S				
2331	relapse	Neg	Neg											Neg						
2332	unknown	1+	Pos	S										Pos	S	S				
2333	relapse	3+	Neg											Neg						
2334	relapse	Neg	Neg											Pos	R	S				
2335	high-burden	2+	Pos	S										Pos	S	S				
2336	unknown	Neg	Neg											Neg						
2337	failure	Neg	Pos	I										Pos	S	S				
2338	relapse	Neg	Neg											Neg						

Pt#	Group	AFS	Xpert			DR*plus* (WT absent, MUT binding)		DR*sl* (WT absent, MUT binding)		Sequencing (Discordant)				Convetional results						
			MTBC	RIF	Probe Failure	RIF	INH	FLQ	SLID	RIF	INH	FLQ	SLID	Cul.	INH	RIF	FLQ	KAN	AMK	CAP
2339	unknown	Neg	Neg											Neg						
2340	high-burden	1+	Pos	R	B	WT3	*katG* WT,MUT1 *inhA* WT1,MUT1	*gyrA* WT2,MUT1	*rrs* WT1,MUT1					Pos	R	R	R	R	R	R
2341	high-burden	Neg	Neg											Neg						
2342	relapse	Neg	Neg											Neg						
2343	relapse	Neg	Neg											Neg						
2344	relapse	2+	Pos	S										Pos	S	S				
2345	failure	Neg	Neg											Neg						
2346	relapse	4+	Neg											NTM						
2347	high-burden	Neg	Neg											Pos	S	S				
2348	high-burden	Neg	Neg											Neg						
2349	high-burden	Neg	Neg											Pos	S	S				
2350	high-burden	Neg	Neg											Pos	S	S				
2351	unknown	Neg	Neg											Neg						
2352	high-burden	Neg	Neg											Neg						
2353	high-burden	3+	Pos	S										Pos	S	S				
2354	high-burden	Neg	Pos	S										Pos	S	S				
2355	failure	2+	Pos	S										NTM						
2356	relapse	1+	Pos	S										Neg						
2357	relapse	Neg	Neg											Neg						
2358	high-burden	-	Pos	S										Pos	-	-				
2359	high-burden	Neg	Neg											Pos	S	S				
2360	relapse	3+	Neg											NTM						
2361	relapse	Neg	Neg											Neg						
2362	relapse	1+	Pos	S										Pos	R	S				
2363	relapse	Neg	Neg											Neg						
2364	relapse	Neg	Pos	S										Pos	S	S				
2365	relapse	3+	Pos	S										Pos	S	S				

Pt#	Group	AFS	Xpert			DR*plus* (WT absent, MUT binding)		DR*sl* (WT absent, MUT binding)		Sequencing (Discordant)				Convetional results						
			MTBC	RIF	Probe Failure	RIF	INH	FLQ	SLID	RIF	INH	FLQ	SLID	Cul.	INH	RIF	FLQ	KAN	AMK	CAP
2366	relapse	Neg	Neg											Pos	S	S				
2367	relapse	S	Neg											Neg						
2368	relapse	Neg	Neg											Neg						
2369	high-burden	-	Pos	S										Pos	S	S				
2370	high-burden	2+	Pos	S										Pos	S	S				
2371	high-burden	Neg	Neg											Neg						
2372	failure	S	Pos	S										Neg						
2373	high-burden	Neg	Pos	S										Pos	S	S	S	S	-	S
2374	failure	1+	Pos	S										NTM						
2375	relapse	-	Pos	S										Neg						
2376	failure	-	Pos	S										Neg						
2377	high-burden	Neg	Neg											Neg						
2378	high-burden	Neg	Neg											Pos	S	S				
2379	high-burden	Neg	Neg											Neg						
2380	relapse	1+	Pos	S										Pos	S	S				
2381	relapse	Neg	Pos	S										Neg						
2382	relapse	1+	Pos	S										Pos	S	S				
2383	unknown	-	Neg											Pos	S	S				
2384	relapse	-	Neg											NTM						
2385	high-burden	S	Pos	S										Pos	S	S				
2386	high-burden	Neg	Neg											Neg						
2387	high-burden	Neg	Neg											Neg						
2388	high-burden	Neg	Pos	S										Pos	S	S				
2389	failure	1+	Neg											Neg						
2390	high-burden	Neg	Neg											Neg						
2391	unknown	2+	Pos	S										Pos	S	S	S	S	-	S
2392	relapse	Neg	Neg											Neg						
2393	failure	3+	Pos	S										Neg						

Pt#	Group	AFS	Xpert			DR*plus* (WT absent, MUT binding)		DR*sl* (WT absent, MUT binding)		Sequencing (Discordant)				Convetional results						
			MTBC	RIF	Probe Failure	RIF	INH	FLQ	SLID	RIF	INH	FLQ	SLID	Cul.	INH	RIF	FLQ	KAN	AMK	CAP
2394	high-burden	Neg	Neg											Pos	S	S				
2395	relapse	Neg	Neg											Neg						
2396	high-burden	Neg	Neg											Neg						
2397	failure	1+	Pos	R	D	WT7	*inhA* WT1,MUT1		*rrs* WT1,MUT 1					Pos	R	R	S	R	R	S
2398	relapse	2+	Neg											NTM						
2399	high-burden	Neg	Pos	S										Pos	R	S				
2400	high-risk	Neg	Neg											Neg						
2401	relapse	Neg	Neg											Neg						
2402	failure	-	Pos	S										Neg						
2403	relapse	Neg	Neg											Neg						
2404	relapse	Neg	Neg											Pos	R	R	S	S	S	S
2405	relapse	Neg	Neg											Pos	S	S				
2406	high-burden	Neg	Neg											Neg						
2407	failure	1+	Pos	S										Neg						
2408	unknown	Neg	Neg											Neg						
2409	failure	1+	Pos	S										Neg						
2410	relapse	2+	Pos	S										Pos	R	S				
2411	high-burden	Neg	Neg											Neg						
2412	high-burden	1+	Neg											NTM						
2413	relapse	-	Neg											Neg						
2414	relapse	1+	Pos	S										Neg						
2415	unknown	-	Neg											Neg						
2416	failure	1+	Pos	S										Neg						
2417	relapse	3+	Pos	S										Pos	S	S	S	S	-	S
2418	unknown	Neg	Neg											Neg						
2419	high-burden	Neg	Pos	S										Pos	S	S				
2420	relapse	Neg	Neg											Neg						

Pt#	Group	AFS	Xpert			DR*plus* (WT absent, MUT binding)		DR*sl* (WT absent, MUT binding)		Sequencing (Discordant)				Convetional results						
			MTBC	RIF	Probe Failure	RIF	INH	FLQ	SLID	RIF	INH	FLQ	SLID	Cul.	INH	RIF	FLQ	KAN	AMK	CAP
2421	relapse	Neg	Pos	S										Neg						
2422	high-burden	Neg	Neg											Neg						
2423	high-burden	S	Pos	S										Pos	S	S				
2424	relapse	4+	Pos	S										Pos	S	S				
2425	relapse	Neg	Neg											Neg						
2426	relapse	Neg	Neg											Pos	S	S				
2427	relapse	Neg	Neg											Neg						
2428	relapse	3+	Pos	S										Pos	S	S				
2429	high-burden	3+	Pos	S										Pos	R	S				
2430	high-risk	Neg	Neg											Pos	S	S				
2431	high-burden	Neg	Neg											Neg						
2432	relapse	4+	Pos	R	B	WT3	S							Pos	S	R	S	S	-	S
2433	relapse	Neg	Neg											Pos	S	S				
2434	failure	1+	Neg											NTM						
2435	relapse	1+	Pos	S										NTM						
2436	high-risk	1+	Pos	S										Pos	R	S				
2437	relapse	S	Pos	S										Pos	S	S				
2438	relapse	Neg	Neg											Neg						
2439	high-burden	1+	Pos	S										Pos	S	S				
2440	close contacts	Neg	Pos	S										Pos	S	S				
2441	relapse	1+	Neg											NTM						
2442	relapse	Neg	Pos	S										Pos	S	S				
2443	relapse	1+	Neg											Neg						
2444	close contacts	3+	Pos	S										Pos	S	S				
2445	relapse	Neg	Pos	S										Pos	S	S				
2446	high-risk	2+	Pos	S										Pos	S	S				
2447	relapse	Neg	Neg											Neg						
2448	high-burden	Neg	Neg											Neg						

Pt#	Group	AFS	Xpert			DR*plus* (WT absent, MUT binding)		DR*sl* (WT absent, MUT binding)		Sequencing (Discordant)				Convetional results						
			MTBC	RIF	Probe Failure	RIF	INH	FLQ	SLID	RIF	INH	FLQ	SLID	Cul.	INH	RIF	FLQ	KAN	AMK	CAP
2449	high-burden	Neg	Neg											Pos	S	S				
2450	high-risk	Neg	Neg											Neg						
2451	relapse	Neg	Neg											Neg						
2452	high-risk	Neg	Neg											Neg						
2453	high-risk	Neg	Neg											Neg						
2454	high-risk	Neg	Neg											Neg						
2455	high-risk	2+	Neg											Pos	S	S				
2456	high-risk	Neg	Neg											Neg						
2457	high-risk	S	Pos	S										Pos	S	S				
2458	high-risk	1+	Pos	S										Pos	S	S				
2459	high-risk	Neg	Neg											Neg						
2460	failure	3+	Pos	R	E	WT8,MUT3	*katG* WT,MUT1							Pos	R	R	S	S	S	S
2461	relapse	Neg	Neg											Neg						
2462	failure	2+	Neg											Missing						
2463	high-burden	Neg	Neg											Pos	S	S				
2464	relapse	Neg	Neg											Neg						
2465	high-risk	S	Pos	S										Pos	S	S				
2466	relapse	1+	Pos	S										Pos	S	S				
2467	high-burden	2+	Pos	S										Pos	S	S				
2468	high-burden	1+	Pos	S										Neg						
2469	high-burden	Neg	Neg											Neg						
2470	high-risk	Neg	Neg											Neg						
2471	close contacts	Neg	Neg											Neg						
2472	failure	1+	Pos	R	E	WT8,MUT3	*katG* WT,MUT1		*rrs* MUT2				*rrs* WT eisWT	Pos	R	R	S	S	-	S
2473	relapse	Neg	Pos	I										NTM						
2474	relapse	1+	Pos	S										Pos	S	S				
2475	relapse	1+	Neg											NTM						

Pt#	Group	AFS	Xpert			DR*plus* (WT absent, MUT binding)		DR*sl* (WT absent, MUT binding)		Sequencing (Discordant)				Convetional results						
			MTBC	RIF	Probe Failure	RIF	INH	FLQ	SLID	RIF	INH	FLQ	SLID	Cul.	INH	RIF	FLQ	KAN	AMK	CAP
2476	high-burden	Neg	Pos	S										Pos	R	S				
2477	close contacts	Neg	Neg											Pos	S	S				
2478	high-burden	Neg	Neg											Neg						
2479	relapse	1+	Neg											NTM						
2480	relapse	1+	Neg											Neg						
2481	relapse	2+	Pos	S										Pos	S	S				
2482	high-burden	Neg	Neg											Neg						
2483	failure	S	Pos	S										Pos	S	S	R	S	-	S
2484	failure	1+	Pos	S										Pos	S	S	S	S	-	S
2485	relapse	S	Neg											Neg						
2486	relapse	S	Pos	S										NTM						
2487	high-risk	4+	Pos	S										Pos	R	S				
2488	failure	Neg	Pos	S										Missing						
2489	high-risk	3+	Pos	S										Pos	S	S				
2490	high-risk	1+	Pos	S										Pos	S	S				
2491	high-burden	Neg	Neg											Neg						
2492	relapse	Neg	Neg											Pos	S	S				
2493	high-burden	1+	Pos	S										Pos	S	S				
2494	default	Neg	Pos	S										Pos	S	S				
2495	relapse	Neg	Neg											Neg						
2496	failure	S	Neg											NTM						
2497	high-burden	Neg	Neg											Neg						
2498	high-burden	Neg	Neg											Neg						
2499	high-risk	Neg	Neg											Neg						
2500	high-risk	Neg	Neg											Neg						
2501	high-burden	1+	Pos	S										Missing						
2502	relapse	Neg	Neg											Neg						
2503	high-burden	Neg	Neg											NTM						

Pt#	Group	AFS	Xpert			DR*plus* (WT absent, MUT binding)		DR*sl* (WT absent, MUT binding)		Sequencing (Discordant)				Convetional results						
			MTBC	RIF	Probe Failure	RIF	INH	FLQ	SLID	RIF	INH	FLQ	SLID	Cul.	INH	RIF	FLQ	KAN	AMK	CAP
2504	relapse	3+	Pos	S										Pos	S	S				
2505	high-risk	Neg	Neg											Neg						
2506	high-burden	Neg	Pos	S										Pos	S	S				
2507	relapse	1+	Neg											NTM						
2508	relapse	Neg	Neg											Neg						
2509	high-burden	Neg	Neg											Neg						
2510	high-burden	Neg	Neg											Neg						
2511	high-burden	Neg	Neg											Neg						
2512	relapse	2+	Pos	S										Pos	S	S				
2513	high-burden	Neg	Neg											Pos	S	S				
2514	high-burden	Neg	Neg											Pos	S	S				
2515	high-burden	2+	Neg											Pos	S	S				
2516	relapse	Neg	Pos	S										Pos	S	S				
2517	high-risk	Neg	Neg											Neg						
2518	high-risk	4+	Pos	S										Pos	S	S				
2519	failure	1+	Neg											Neg						
2520	high-risk	Neg	Neg											Neg						
2521	high-risk	3+	Neg											NTM						
2522	relapse	3+	Neg											Neg						
2523	high-burden	Neg	Neg											Neg						
2524	relapse	Neg	Neg											Neg						
2525	relapse	2+	Pos	S										Pos	S	S				
2526	high-burden	S	Neg											Neg						
2527	high-risk	1+	Pos	S										Pos	S	S				
2528	high-risk	4+	Pos	S										Pos	S	S				
2529	relapse	Neg	Neg											Neg						
2530	high-burden	Neg	Neg											Pos	R	S				
2531	high-burden	Neg	Neg											Pos	S	S				

Pt#	Group	AFS	Xpert			DR*plus* (WT absent, MUT binding)		DR*sl* (WT absent, MUT binding)		Sequencing (Discordant)				Convetional results						
			MTBC	RIF	Probe Failure	RIF	INH	FLQ	SLID	RIF	INH	FLQ	SLID	Cul.	INH	RIF	FLQ	KAN	AMK	CAP
2532	relapse	Neg	Neg											NTM						
2533	high-risk	3+	Neg											NTM						
2534	relapse	1+	Neg											NTM						
2535	high-risk	-	Pos	S										Pos	S	S				
2536	high-burden	Neg	Neg											Pos	S	S	S	S	-	S
2537	high-burden	Neg	Neg											Neg						
2538	relapse	3+	Pos	S										Pos	S	S				
2539	relapse	Neg	Neg											NTM						
2540	high-burden	1+	Pos	S										Pos	S	S				
2541	high-risk	Neg	Neg											Neg						
2542	high-risk	1+	Neg											Neg						
2543	relapse	1+	Neg											Neg						
2544	high-burden	S	Pos	S										Neg						
2545	high-burden	Neg	Neg											Pos	S	S				
2546	high-risk	-	Pos	S										Pos	R	S				
2547	relapse	3+	Neg											NTM						
2548	high-risk	Neg	Neg											Neg						
2549	failure	3+	Pos	S										Neg						
2550	high-risk	-	Neg											Pos	S	S				
2551	relapse	1+	Pos	S										Pos	S	S				
2552	high-burden	Neg	Neg											Pos	S	S				
2553	high-risk	Neg	Neg											Neg						
2554	failure	S	Pos	S										Neg						
2555	high-risk	1+	Pos	S										Pos	S	S				
2556	high-burden	4+	Pos	S										Pos	S	S				
2557	high-burden	Neg	Neg											Neg						
2558	high-risk	-	Pos	S										Pos	S	S				
2559	relapse	S	Pos	S										Pos	S	S				

Pt#	Group	AFS	Xpert			DR*plus* (WT absent, MUT binding)		DR*sl* (WT absent, MUT binding)		Sequencing (Discordant)				Convetional results						
			MTBC	RIF	Probe Failure	RIF	INH	FLQ	SLID	RIF	INH	FLQ	SLID	Cul.	INH	RIF	FLQ	KAN	AMK	CAP
2560	high-burden	-	Neg											Pos	S	S				
2561	high-burden	Neg	Neg											Pos	S	S				
2562	high-risk	3+	Pos	S										Pos	S	S				
2563	high-burden	Neg	Neg											Pos	S	S	S	S	-	S
2564	high-burden	1+	Pos	S										Pos	S	S	S	S	-	S
2565	failure	1+	Pos	S										Neg						
2566	high-burden	Neg	Neg											Neg						
2567	relapse	1+	Pos	S										Pos	S	S				
2568	default	Neg	Neg											Neg						
2569	high-risk	Neg	Neg											NTM						
2570	relapse	Neg	Neg											Neg						
2571	failure	1+	Pos	S										Neg						
2572	failure	2+	Neg											Neg						
2573	relapse	Neg	Neg											Neg						
2574	high-burden	Neg	Neg											Pos	S	S				
2575	high-risk	Neg	Neg											Neg						
2576	high-burden	-	Pos	S										Pos	S	S				
2577	high-risk	Neg	Neg											NTM						
2578	relapse	Neg	Neg											Neg						
2579	default	Neg	Neg											Neg						
2580	high-risk	Neg	Neg											NTM						
2581	high-burden	Neg	Neg											Neg						
2582	failure	-	Pos	S										Pos	S	S				
2583	relapse	3+	Pos	S										Pos	S	S				
2584	failure	Neg	Pos	S										Pos	S	S				
2585	high-burden	Neg	Neg											Pos	S	S				
2586	relapse	1+	Neg											Neg						
2587	high-risk	-	Pos	S										Pos	R	S				

Pt#	Group	AFS	Xpert			DR*plus* (WT absent, MUT binding)		DR*sl* (WT absent, MUT binding)		Sequencing (Discordant)				Convetional results						
			MTBC	RIF	Probe Failure	RIF	INH	FLQ	SLID	RIF	INH	FLQ	SLID	Cul.	INH	RIF	FLQ	KAN	AMK	CAP
2588	relapse	3+	Pos	S										Pos	S	S				
2589	unknown	Neg	Neg											NTM						
2590	unknown	1+	Pos	S										Pos	S	S	S	S	-	S
2591	high-risk	Neg	Neg											Neg						
2592	high-risk	Neg	Neg											Neg						
2593	high-burden	Neg	Neg											Pos	S	S				
2594	relapse	-	Neg											NTM						
2595	relapse	3+	Pos	S										Pos	S	S				
2596	failure	1+	Pos	S										Neg						
2597	unknown	-	Pos	S										Neg						
2598	relapse	1+	Pos	S										Pos	S	S				
2599	high-risk	Neg	Pos	S										Pos	S	S				
2600	relapse	-	Neg											Neg						
2601	high-burden	Neg	Neg											Pos	S	S				
2602	relapse	4+	Neg											Neg						
2603	high-burden	Neg	Neg											Neg						
2604	high-risk	-	Pos	S										Pos	S	S				
2605	high-risk	Neg	Neg											Neg						
2606	high-risk	Neg	Neg											Neg						
2607	high-risk	4+	Pos	S										Pos	S	S				
2608	default	-	Pos	S										Pos	S	S				
2609	high-burden	Neg	Neg											Neg						
2610	relapse	S	Neg											Neg						
2611	high-burden	Neg	Neg											Neg						
2612	high-burden	2+	Pos	S										Pos	S	S				
2613	high-risk	Neg	Neg											Neg						
2614	failure	1+	Pos	S										NTM						
2615	high-burden	1+	Neg											Pos	S	S				

Pt#	Group	AFS	Xpert			DR*plus* (WT absent, MUT binding)		DR*sl* (WT absent, MUT binding)		Sequencing (Discordant)				Convetional results						
			MTBC	RIF	Probe Failure	RIF	INH	FLQ	SLID	RIF	INH	FLQ	SLID	Cul.	INH	RIF	FLQ	KAN	AMK	CAP
2616	high-burden	Neg	Neg											Neg						
2617	high-burden	Neg	Neg											Neg						
2618	relapse	Neg	Neg											NTM						
2619	failure	1+	Pos	S										Neg						
2620	relapse	1+	Pos	S										Pos	S	S				
2621	relapse	-	Pos	S										Pos	R	S				
2622	high-burden	Neg	Neg											Neg						
2623	unknown	Neg	Neg											Neg						
2624	relapse	Neg	Pos	S										Pos	R	S				
2625	high-burden	3+	Pos	S										Pos	S	S				
2626	high-burden	Neg	Neg											Pos	S	S				
2627	high-burden	Neg	Neg											Neg						
2628	high-burden	Neg	Neg											Neg						
2629	high-burden	Neg	Neg											Neg						
2630	high-burden	Neg	Neg											Neg						
2631	failure	1+	Pos	S										Neg						
2632	high-risk	1+	Pos	S										Pos	S	S				
2633	high-risk	Neg	Neg											Neg						
2634	relapse	1+	Neg											Neg						
2635	relapse	Neg	Neg											Neg						
2636	high-burden	Neg	Neg											Neg						
2637	relapse	Neg	Neg											Neg						
2638	relapse	4+	Pos	S										Pos	S	S				
2639	high-burden	Neg	Neg											Neg						
2640	high-burden	Neg	Pos	S										Pos	S	S				
2641	failure	3+	Pos	S										Pos	S	S				
2642	relapse	Neg	Pos	S										Neg						
2643	high-burden	Neg	Neg											Neg						

Pt#	Group	AFS	Xpert			DR*plus* (WT absent, MUT binding)		DR*sl* (WT absent, MUT binding)		Sequencing (Discordant)				Convetional results						
			MTBC	RIF	Probe Failure	RIF	INH	FLQ	SLID	RIF	INH	FLQ	SLID	Cul.	INH	RIF	FLQ	KAN	AMK	CAP
2644	relapse	1+	Pos	S										Pos	S	S				
2645	close contacts	Neg	Neg											Neg						
2646	high-burden	Neg	Neg											Neg						
2647	high-risk	Neg	Neg											Neg						
2648	relapse	2+	Pos	S										Pos	S	S				
2649	relapse	-	Neg											Pos	S	S				
2650	high-risk	Neg	Neg											Neg						
2651	high-burden	S	Neg											Neg						
2652	relapse	Neg	Neg											Neg						
2653	high-burden	Neg	Neg											Neg						
2654	unknown	Neg	Neg											Neg						
2655	default	4+	Pos	S										Pos	S	S				
2656	high-burden	Neg	Pos	S										Neg						
2657	relapse	S	Neg											Neg						
2658	high-burden	Neg	Neg											Neg						
2659	failure	1+	Neg											Missing						
2660	high-burden	Neg	Neg											Pos	S	S				
2661	failure	1+	Neg											Missing						
2662	high-burden	1+	Pos	S										Pos	S	S				
2663	relapse	Neg	Neg											NTM						
2664	relapse	2+	Neg											NTM						
2665	high-burden	Neg	Neg											Pos	S	S				
2666	high-burden	2+	Pos	S										Pos	R	S				
2667	failure	1+	Pos	S										Pos	S	S				
2668	failure	1+	Neg											Missing						
2669	relapse	S	Pos	S										NTM						
2670	relapse	Neg	Neg											Pos	S	S				
2671	failure	1+	Pos	S										Missing						

Pt#	Group	AFS	Xpert			DR*plus* (WT absent, MUT binding)		DR*sl* (WT absent, MUT binding)		Sequencing (Discordant)				Convetional results						
			MTBC	RIF	Probe Failure	RIF	INH	FLQ	SLID	RIF	INH	FLQ	SLID	Cul.	INH	RIF	FLQ	KAN	AMK	CAP
2672	failure	Neg	Neg											Missing						
2673	high-burden	1+	Pos	S										Pos	S	S				
2674	failure	2+	Pos	S										NTM						
2675	relapse	Neg	Neg											Missing						
2676	failure	-	Pos	S										Missing						
2677	failure	1+	Pos	S										Missing						
2678	relapse	2+	Pos	S										Missing						
2679	close contacts	Neg	Neg											Pos	R	S				
2680	relapse	1+	Pos	S										Missing						
2681	high-risk	3+	Pos	S										Pos	S	S				
2682	failure	1+	Pos	S										Pos	S	S				
2683	failure	Neg	Neg											Missing						
2684	relapse	S	Neg											NTM						
2685	relapse	1+	Neg											NTM						
2686	high-burden	Neg	Neg											Missing						
2687	relapse	Neg	Neg											Missing						
2688	high-burden	Neg	Neg											Neg						
2689	relapse	Neg	Neg											Missing						
2690	failure	S	Pos	S										Missing						
2691	high-burden	Neg	Neg											Missing						
2692	relapse	3+	Pos	S										Pos	S	S				
2693	relapse	1+	Neg											Missing						
2694	failure	2+	Pos	S										NTM						
2695	relapse	1+	Neg											Missing						
2696	high-burden	Neg	Neg											Missing						
2697	high-burden	Neg	Neg											Missing						
2698	high-burden	Neg	Neg											Neg						
2699	high-burden	Neg	Neg											Missing						

Pt#	Group	AFS	Xpert			DR*plus* (WT absent, MUT binding)		DR*sl* (WT absent, MUT binding)		Sequencing (Discordant)				Convetional results						
			MTBC	RIF	Probe Failure	RIF	INH	FLQ	SLID	RIF	INH	FLQ	SLID	Cul.	INH	RIF	FLQ	KAN	AMK	CAP
2700	high-burden	Neg	Neg											Pos	S	S				
2701	relapse	1+	Pos	S										Missing						
2702	failure	1+	Neg											Missing						
2703	default	1+	Pos	S										Missing						
2704	relapse	Neg	Neg											Missing						
2705	relapse	1+	Pos	S										Missing						
2706	relapse	1+	Neg											Neg						
2707	high-burden	Neg	Neg											Pos	S	S				
2708	high-burden	1+	Pos	S										Neg						
2709	relapse	Neg	Neg											Neg						
2710	high-burden	1+	Neg											Missing						
2711	high-burden	Neg	Neg											Neg						
2712	high-burden	Neg	Pos	S										Pos	S	S				
2713	failure	2+	Pos	S										NTM						
2714	failure	1+	Neg											Missing						
2715	failure	1+	Neg											Missing						
2716	relapse	1+	Neg											Missing						
2717	relapse	Neg	Neg											Missing						
2718	high-burden	Neg	Pos	S										Pos	R	S				
2719	high-burden	Neg	Neg											Missing						
2720	high-risk	4+	Pos	S										Pos	S	S				
2721	failure	1+	Pos	S										Pos	S	S				
2722	failure	1+	Neg											NTM						
2723	high-risk	Neg	Neg											Missing						
2724	high-burden	Neg	Neg											Missing						
2725	relapse	4+	Neg											Missing						
2726	failure	1+	Neg											Missing						
2727	high-burden	1+	Neg											Missing						

Pt#	Group	AFS	Xpert			DR*plus* (WT absent, MUT binding)		DR*sl* (WT absent, MUT binding)		Sequencing (Discordant)				Convetional results						
			MTBC	RIF	Probe Failure	RIF	INH	FLQ	SLID	RIF	INH	FLQ	SLID	Cul.	INH	RIF	FLQ	KAN	AMK	CAP
2728	high-risk	1+	Pos	S										Pos	S	S				
2729	high-burden	Neg	Neg											Missing						
2730	high-burden	Neg	Neg											NTM						
2731	relapse	4+	Neg											Missing						
2732	high-burden	1+	Pos	S										Pos	S	S				
2733	high-burden	Neg	Neg											Missing						
2734	failure	3+	Pos	S										Missing						
2735	failure	1+	Pos	S										Missing						
2736	relapse	Neg	Neg											Missing						
2737	failure	S	Pos	S										NTM						
2738	high-burden	Neg	Neg											Missing						
2739	high-burden	Neg	Neg											Missing						
2740	relapse	Neg	Neg											Missing						
2741	close contacts	4+	Pos	S										Missing						
2742	high-burden	Neg	Neg											Missing						
2743	high-burden	S	Pos	S										Pos	S	S				
2744	failure	1+	Pos	S										NTM						
2745	high-burden	Neg	Neg											Missing						
2746	high-risk	Neg	Neg											Missing						
2747	relapse	Neg	Pos	S										Pos	S	S				
2748	high-burden	Neg	Neg											Missing						
2749	high-burden	2+	Pos	S										Pos	S	S				
2750	failure	1+	Pos	S										Missing						
2751	failure	S	Pos	S										NTM						
2752	high-risk	Neg	Neg											Missing						
2753	failure	1+	Neg											NTM						
2754	high-burden	Neg	Neg											Missing						
2755	high-burden	Neg	Neg											Missing						

Pt#	Group	AFS	Xpert			DR*plus* (WT absent, MUT binding)		DR*sl* (WT absent, MUT binding)		Sequencing (Discordant)				Convetional results						
			MTBC	RIF	Probe Failure	RIF	INH	FLQ	SLID	RIF	INH	FLQ	SLID	Cul.	INH	RIF	FLQ	KAN	AMK	CAP
2756	high-burden	Neg	Neg											Missing						
2757	high-burden	Neg	Neg											Missing						
2758	failure	1+	Neg											Missing						
2759	relapse	Neg	Neg											Neg						
2760	relapse	1+	Neg											Missing						
2761	default	S	Neg											Pos	S	S				
2762	failure	S	Neg											Missing						
2763	high-burden	Neg	Neg											Missing						
2764	high-burden	Neg	Neg											Pos	S	S				
2765	high-risk	Neg	Neg											Missing						
2766	relapse	S	Pos	R	A	-	-							Missing						
2767	relapse	-	Neg											Missing						
2768	failure	S	Neg											Missing						
2769	relapse	4+	Neg											Missing						
2770	high-burden	2+	Pos	S										Pos	S	S				
2771	high-risk	1+	Pos	S										Missing						
2772	relapse	Neg	Neg											Missing						
2773	relapse	Neg	Neg											Missing						
2774	high-burden	1+	Neg											Missing						
2775	failure	1+	Pos	S										Missing						
2776	high-burden	Neg	Neg											Missing						
2777	failure	3+	Pos	S										Pos	S	S				
2778	high-risk	Neg	Neg											Missing						
2779	high-risk	2+	Pos	S										Pos	S	S				
2780	high-burden	Neg	Neg											Missing						
2781	high-burden	Neg	Neg											Missing						
2782	high-risk	4+	Pos	S										Pos	S	S				
2783	relapse	Neg	Neg											Missing						

Pt#	Group	AFS	Xpert			DR*plus* (WT absent, MUT binding)		DR*sl* (WT absent, MUT binding)		Sequencing (Discordant)				Convetional results						
			MTBC	RIF	Probe Failure	RIF	INH	FLQ	SLID	RIF	INH	FLQ	SLID	Cul.	INH	RIF	FLQ	KAN	AMK	CAP
2784	high-burden	Neg	Neg											Missing						
2785	failure	1+	Neg											NTM						
2786	relapse	4+	Pos	S										Pos	S	S				
2787	high-burden	Neg	Neg											Missing						
2788	high-burden	Neg	Pos	I										Missing						
2789	high-burden	Neg	Neg											Missing						
2790	relapse	4+	Pos	S										Pos	S	S				
2791	relapse	Neg	Neg											Missing						
2792	high-burden	Neg	Neg											Missing						
2793	relapse	Neg	Neg											Missing						
2794	high-burden	Neg	Neg											Missing						
2795	high-burden	Neg	Neg											Missing						
2796	high-burden	1+	Neg											Missing						
2797	high-burden	Neg	Neg											Missing						
2798	high-burden	Neg	Neg											Missing						
2799	high-burden	Neg	Neg											Missing						
2800	default	-	Neg											Missing						
2801	failure	1+	Pos	S										NTM						
2802	high-burden	Neg	Neg											Missing						
2803	high-burden	Neg	Neg											Missing						
2804	high-risk	1+	Pos	S										Pos	S	S				
2805	high-burden	Neg	Neg											Missing						
2806	high-burden	2+	Pos	S										Pos	S	S				
2807	high-risk	2+	Pos	S										Pos	S	S				
2808	high-burden	-	Neg											Missing						
2809	failure	1+	Neg											Missing						
2810	high-burden	Neg	Neg											Missing						
2811	high-burden	Neg	Neg											Missing						

Pt#	Group	AFS	Xpert			DR*plus* (WT absent, MUT binding)		DR*sl* (WT absent, MUT binding)		Sequencing (Discordant)				Convetional results						
			MTBC	RIF	Probe Failure	RIF	INH	FLQ	SLID	RIF	INH	FLQ	SLID	Cul.	INH	RIF	FLQ	KAN	AMK	CAP
2812	failure	1+	Neg											NTM						
2813	failure	Neg	Pos	S										Missing						
2814	high-burden	Neg	Neg											Missing						
2815	high-burden	1+	Pos	S										Pos	R	S				
2816	relapse	Neg	Neg											Missing						
2817	high-risk	-	Neg											Missing						
2818	high-burden	-	Neg											Missing						
2819	failure	1+	Neg											Missing						
2820	high-burden	Neg	Neg											Missing						
2821	high-burden	Neg	Neg											Missing						
2822	failure	3+	Neg											Missing						
2823	high-burden	Neg	Neg											Missing						
2824	relapse	Neg	Neg											Missing						
2825	high-burden	Neg	Neg											Missing						
2826	relapse	1+	Pos	S										Missing						
2827	high-burden	Neg	Neg											Missing						
2828	relapse	1+	Pos	S										Missing						
2829	high-burden	Neg	Neg											Missing						
2830	unknown	Neg	Neg											Neg						
2831	relapse	Neg	Neg											Missing						
2832	relapse	1+	Neg											Missing						
2833	high-risk	-	Neg											Missing						
2834	high-risk	-	Neg											Pos	S	S				
2835	high-risk	Neg	Pos	S										Pos	S	S				
2836	high-burden	1+	Pos	S										Pos	S	S				
2837	high-burden	3+	Pos	S										Pos	S	S				
2838	high-burden	Neg	Neg											Missing						
2839	high-burden	Neg	Neg											Missing						

Pt#	Group	AFS	Xpert			DR*plus* (WT absent, MUT binding)		DR*sl* (WT absent, MUT binding)		Sequencing (Discordant)				Convetional results						
			MTBC	RIF	Probe Failure	RIF	INH	FLQ	SLID	RIF	INH	FLQ	SLID	Cul.	INH	RIF	FLQ	KAN	AMK	CAP
2840	relapse	1+	Pos	R	A	WT2	*inhA* WT1,MUT1			L511P				Pos	R	S	S	S	S	S
2841	high-risk	-	Neg											Missing						
2842	high-burden	-	Neg											Missing						
2843	relapse	-	Neg											Pos	R	S				
2844	relapse	-	Pos	S										Pos	S	S				
2845	high-burden	Neg	Neg											Neg						
2846	relapse	Neg	Neg											Pos	S	S				
2847	failure	1+	Pos	S										Pos	S	S				
2848	high-burden	Neg	Neg											Neg						
2849	high-burden	Neg	Neg											Missing						
2850	failure	1+	Pos	S										Missing						
2851	failure	1+	Pos	S										Missing						
2852	relapse	Neg	Pos	S										Missing						
2853	high-burden	Neg	Neg											Missing						
2854	high-burden	Neg	Neg											Missing						
2855	high-burden	Neg	Neg											Missing						
2856	relapse	-	Neg											Missing						
2857	failure	Neg	Neg											Missing						
2858	high-burden	-	Neg											Missing						
2859	relapse	Neg	Neg											NTM						
2860	high-burden	Neg	Neg											Missing						
2861	high-risk	-	Neg											Neg						
2862	high-burden	Neg	Neg											Missing						
2863	high-burden	Neg	Neg											Missing						
2864	high-burden	1+	Neg											Missing						
2865	high-burden	S	Pos	S										Pos	S	S	S	S	-	S
2866	relapse	-	Neg											Missing						
2867	high-burden	1+	Pos	S										Pos	S	S	S	S	-	S

Pt#	Group	AFS	Xpert			DR*plus* (WT absent, MUT binding)		DR*sl* (WT absent, MUT binding)		Sequencing (Discordant)				Convetional results						
			MTBC	RIF	Probe Failure	RIF	INH	FLQ	SLID	RIF	INH	FLQ	SLID	Cul.	INH	RIF	FLQ	KAN	AMK	CAP
2868	default	Neg	Neg											Missing						
2869	relapse	Neg	Neg											NTM						
2870	high-burden	Neg	Neg											Missing						
2871	high-burden	1+	Neg											Missing						
2872	unknown	1+	Pos	S										Missing						
2873	high-burden	Neg	Neg											Missing						
2874	high-burden	Neg	Neg											Missing						
2875	high-burden	Neg	Neg											Missing						
2876	high-burden	1+	Pos	S										Pos	S	S				
2877	failure	1+	Neg											Missing						
2878	failure	1+	Neg											Missing						
2879	high-burden	Neg	Neg											Missing						
2880	high-burden	-	Neg											Missing						
2881	high-burden	Neg	Neg											Pos	S	S				
2882	high-burden	-	Neg											Missing						
2883	high-burden	Neg	Pos	S										Missing						
2884	high-burden	Neg	Neg											Missing						
2885	high-risk	-	Neg											Missing						
2886	high-risk	-	Neg											Missing						
2887	high-burden	Neg	Neg											Missing						
2888	high-burden	Neg	Neg											Missing						
2889	close contacts	Neg	Neg											Missing						
2890	high-burden	Neg	Neg											Missing						
2891	high-burden	-	Neg											Missing						
2892	high-burden	-	Neg											Missing						
2893	high-burden	Neg	Neg											Missing						
2894	high-burden	Neg	Neg											Missing						
2895	high-burden	Neg	Neg											Missing						

Pt#	Group	AFS	Xpert			DR*plus* (WT absent, MUT binding)		DR*sl* (WT absent, MUT binding)		Sequencing (Discordant)				Convetional results						
			MTBC	RIF	Probe Failure	RIF	INH	FLQ	SLID	RIF	INH	FLQ	SLID	Cul.	INH	RIF	FLQ	KAN	AMK	CAP
2896	unknown	1+	Pos	S										Pos	S	S				
2897	high-burden	-	Neg											Missing						
2898	high-burden	Neg	Neg											Missing						
2899	high-burden	Neg	Neg											Missing						
2900	high-burden	Neg	Neg											Missing						
2901	high-burden	Neg	Neg											Missing						
2902	high-burden	-	Neg											Missing						
2903	failure	Neg	Pos	S										Pos	S	S				
2904	high-burden	1+	Neg											Pos	S	S				
2905	failure	2+	Pos	S										Missing						
2906	high-burden	Neg	Neg											Missing						
2907	high-burden	-	Neg											Missing						
2908	relapse	Neg	Neg											Missing						
2909	high-burden	Neg	Neg											Neg						
2910	high-risk	S	Neg											Pos	S	S				
2911	unknown	-	Pos	S										Pos	S	S	S	S	-	S
2912	high-burden	Neg	Neg											Missing						
2913	high-burden	-	Neg											Missing						
2914	failure	4+	Neg											Missing						
2915	high-burden	Neg	Neg											Missing						
2916	unknown	Neg	Neg											Missing						
2917	high-burden	-	Neg											Missing						
2918	high-burden	Neg	Neg											Missing						
2919	high-burden	Neg	Neg											Missing						
2920	failure	1+	Neg											NTM						
2921	close contacts	Neg	Neg											Missing						
2922	high-burden	Neg	Neg											Missing						
2923	unknown	-	Neg											Missing						

Pt#	Group	AFS	Xpert			DR*plus* (WT absent, MUT binding)		DR*sl* (WT absent, MUT binding)		Sequencing (Discordant)				Convetional results						
			MTBC	RIF	Probe Failure	RIF	INH	FLQ	SLID	RIF	INH	FLQ	SLID	Cul.	INH	RIF	FLQ	KAN	AMK	CAP
2924	high-burden	1+	Neg											Missing						
2925	high-burden	Neg	Neg											Missing						
2926	high-risk	Neg	Neg											Missing						
2927	relapse	S	Pos	S										NTM						
2928	high-burden	Neg	Neg											Missing						
2929	default	Neg	Neg											Missing						
2930	high-burden	Neg	Neg											Missing						
2931	high-risk	1+	Pos	S										Pos	S	S				
2932	high-burden	-	Neg											Neg						
2933	high-burden	1+	Pos	S										Pos	S	S				
2934	failure	1+	Pos	S										Missing						
2935	relapse	1+	Pos	S										Pos	S	S				
2936	high-risk	Neg	Neg											Pos	S	S				
2937	high-burden	-	Neg											Missing						
2938	unknown	-	Neg											Missing						
2939	unknown	Neg	Neg											NTM						
2940	high-burden	-	Neg											Missing						
2941	high-burden	Neg	Neg											Missing						
2942	failure	1+	Pos	S										Missing						
2943	high-burden	Neg	Neg											Missing						
2944	unknown	Neg	Neg											Missing						
2945	relapse	2+	Neg											Missing						
2946	high-burden	Neg	Neg											Missing						
2947	high-burden	Neg	Neg											Missing						
2948	high-burden	Neg	Neg											Missing						
2949	high-burden	Neg	Neg											Missing						
2950	failure	S	Pos	S										Missing						
2951	unknown	Neg	Neg											Missing						

Pt#	Group	AFS	Xpert			DR*plus* (WT absent, MUT binding)		DR*sl* (WT absent, MUT binding)		Sequencing (Discordant)				Convetional results						
			MTBC	RIF	Probe Failure	RIF	INH	FLQ	SLID	RIF	INH	FLQ	SLID	Cul.	INH	RIF	FLQ	KAN	AMK	CAP
2952	unknown	Neg	Neg											Missing						
2953	relapse	Neg	Pos	S										NTM						
2954	relapse	Neg	Neg											Missing						
2955	high-burden	Neg	Neg											Missing						
2956	high-burden	Neg	Neg											Missing						
2957	high-burden	Neg	Neg											Missing						
